# Continuous Synthesis of Carbamates from CO_2_ and Amines

**DOI:** 10.1021/acsomega.3c08248

**Published:** 2023-12-05

**Authors:** Kristof Stagel, Laura Ielo, Katharina Bica-Schröder

**Affiliations:** † Institute of Applied Synthetic Chemistry, TU Wien, Getreidemarkt 9/163, Vienna 1060, Austria; ‡ Department of Chemistry, University of Turin, Via P. Giuria 7, Torino 10125, Italy

## Abstract

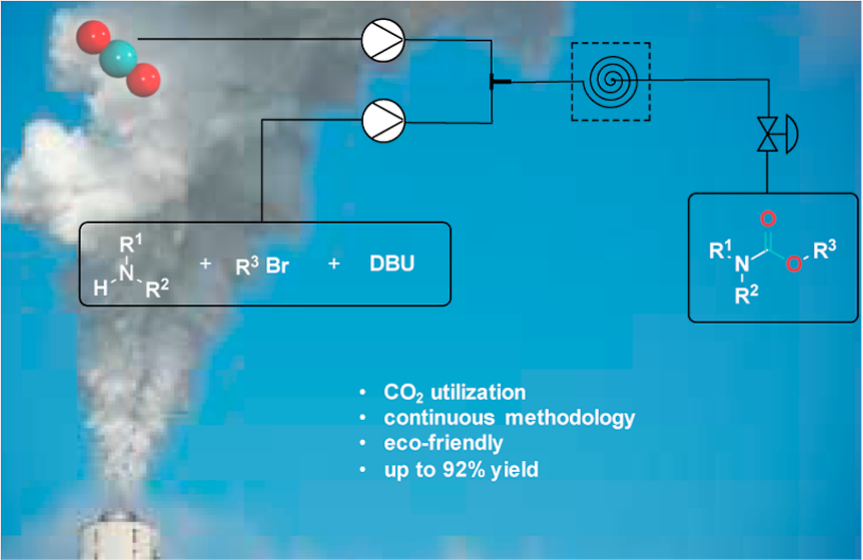

We present a novel approach for the continuous preparation of carbamates. The simple yet fast synthetic route relies on directly utilizing carbon dioxide and, in contrast with the literature-known methods, only employs 1,8-diazabicyclo[5.4.0]undec-7-ene as an additive. The applicable amines’ diversity offers considerable flexibility to the synthetic protocol. Additionally, the continuous method’s applicability significantly decreases the reaction time typically required for CO_2_-based carbamate synthesis and allows for straightforward and precise gas introduction. The mild reaction conditions and omission of the need for column chromatography render the process less time-demanding and environmentally more benign, providing the desired compounds in yields of 45 to 92%. Moreover, the modified procedure can potentially be applied in the selective synthesis of oxazolidinones from aziridines.

## Introduction

Since carbon dioxide is considered the primary contributor to global warming, its valorization has become the most severe task the chemical society faces in the 21st century.^1,2^ The annual CO_2_ emission reached approximately 36.6 gigatonnes, demanding urgent actions to avoid an irreversible disaster.^3^ So far, chemists have developed suitable approaches for utilizing carbon dioxide, among which its use as a C1 building block provides an attractive strategy.^4−7^ Using CO_2_ in chemical transformations has received significant scientific interest in the past decade since it is a direct way to harvest nature’s carbon resources and simultaneously utilize nontoxic starting materials.^8−10^ Carbon dioxide has been successfully valorized in synthesizing chemically invaluable species, such as alcohols,^11,12^ carboxylic acids,^13^ carbonates,^14^ or carbamates.^15^

Urethanes are considered essential structural moieties of a plethora of bioactive compounds, such as agricultural chemicals or therapeutic agents.^16,17^ Moreover, organic carbamates are utilized in the synthesis of polyurethanes.^18^ Carbamates have found widespread application in synthetic chemistry; for example, many of the most popular protecting groups in peptide synthesis are introduced as carbamate moieties.^19,20^

The industrial synthesis of organic carbamates relied on reacting alkyl isocyanates with alcohols.^21^ Isocyanates are considered highly toxic reagents, as evidenced by the disastrous chemical accidents in the past;^22^ thus, they are often formed in situ to avoid safety hazards associated with handling large amounts of isocyanates. Other strategies react alkyl chloroformates with amines, thus generating at least stoichiometric amounts of HCl as a waste byproduct. Additionally, the chloroformate-based synthetic route is also hampered by long reaction times and a large excess of base is required to acquire an acceptable conversion.^17^ Methods relying on the direct fixation of CO_2_ have become more relevant in the past decade and provide an attractive alternative synthetic route that circumvents toxic alkyl isocyanates or costly metal catalysts while simultaneously utilizing an abundant C1 source and building block.

The first method employing carbon dioxide, amines, and alkyl halides in the presence of a base was reported by McGhee and co-workers.^15^ The group investigated the influence of different bases on the reaction selectivity and yield, and they also conducted mechanistic studies. Based on their research, the reaction proceeds through the formation of an ionic intermediate, and it can be accelerated by adding strong non-nucleophilic bases.

Dindarloo and co-workers utilized deep eutectic solvents (DESs) to synthesize carbamates.^23^ The choline chloride-based DES was found to be suitable for the process as less reactive alkyl chlorides were successfully utilized, and the desired carbamates were isolated in good yields.

Yoshida et al. investigated different quaternary onium salts for synthesizing carbamates using supercritical carbon dioxide as solvent and reagent.^24^ Employing tetraalkylammonium halides, in particular, tetrabutylammonium bromide, had a positive influence on the reaction yield.

In 2011, a PEG-promoted method for carbamate synthesis was reported by Kong.^25^ The approach effectively suppressed the formation of the undesired byproducts and provided the desired products under mild conditions.

As the application of zeolites in chemical transformations has emerged, a zeolite-assisted synthetic method for producing carbamates was reported in the early 2000s. Srivastava employed metal complex-containing zeolite catalyst frameworks, and the optimal conditions provided the desired products with good selectivity.^26^

In recent years, various zinc-based catalyst systems have been described. Biswas et al. established a method relying on a polymer-bound zinc(II) complex to synthesize benzimidazole derivatives and carbamates.^27^ The recyclable heterogeneous catalyst system proved to be effective and enabled the reaction to proceed under environmentally benign conditions, such as low pressure and temperature. The same research group reported a graphene oxide-based zinc composite as an efficient catalyst for CO_2_ fixation through the synthesis of carbamates.^28^ The group also described a method relying on titanium phosphate to convert epoxides and amines to carbonates and carbamates, respectively.^29^

Evidently, various methods for the fixation of CO_2_ through carbamate synthesis are available. However, they have certain limitations: some require elevated pressures or temperatures, whereas others are hampered by long reaction times or low reactivity or reproducibility. Almost all the developed methods require additional catalysts, some of which are noncommercial and require a multistep synthetic pathway. Moreover, all the reported strategies have been developed for batch reactions, and none of the above-mentioned approaches were investigated in the continuous mode.

Here, we report for the first time a continuous approach that employs no additional catalyst, performs under mild reaction conditions, and provides the desired carbamates in just 50 min. Additionally, the synthesized carbamates are, with few exceptions, analytically pure after an acidic workup, and no further purification was needed, which renders the approach more environmentally benign.

## Results and Discussion

**Scheme 1:**
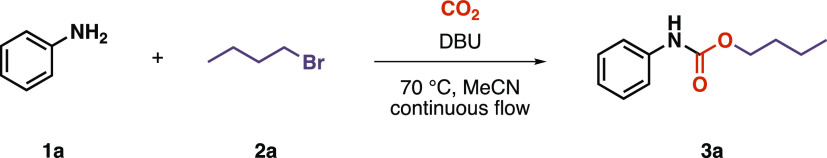
Continuous Synthesis of *N*-Phenyl Butylcarbamate

Based on the results of McGhee in the CO_2_-based synthesis of carbamates,^15^ we initially investigated the synthesis of *N*-phenyl butylcarbamate, starting from aniline and butyl bromide, employing 1,8-diazabicyclo[5.4.0]undec-7-ene (DBU) as a base (Scheme 1). DBU’s liquid nature is beneficial for the continuous process; hence, its employment provides a homogeneous mixture. Economically more viable alternatives, such as triethylamine and *N*,*N*-diisopropylethylamine, were investigated as well, but no conversion was observed when these bases were employed.

Initially, an excess of 2 equiv of alkyl halide and DBU were employed. All of the reactants were dissolved in acetonitrile. Carbon dioxide was introduced directly from a gas bottle using a mass flow controller. The bottle was connected to the flow chemistry device with metal tubing; the V3 pumps efficiently mixed CO_2_ with the reaction mixture in a 10 mL coil reactor.

It is worth mentioning that there was no need to employ a gas–liquid tube-in-tube reactor; hence, dynamic mixing can be achieved in the standard coil reactor if enough residence time is allowed for the reaction to be depleted. Therefore, the flow rate of the reaction mixture was set to 250 μL/min. The reactions were carried out at 70 °C, and the back-pressure regulator was set to 3 bar.

**Scheme 2:**
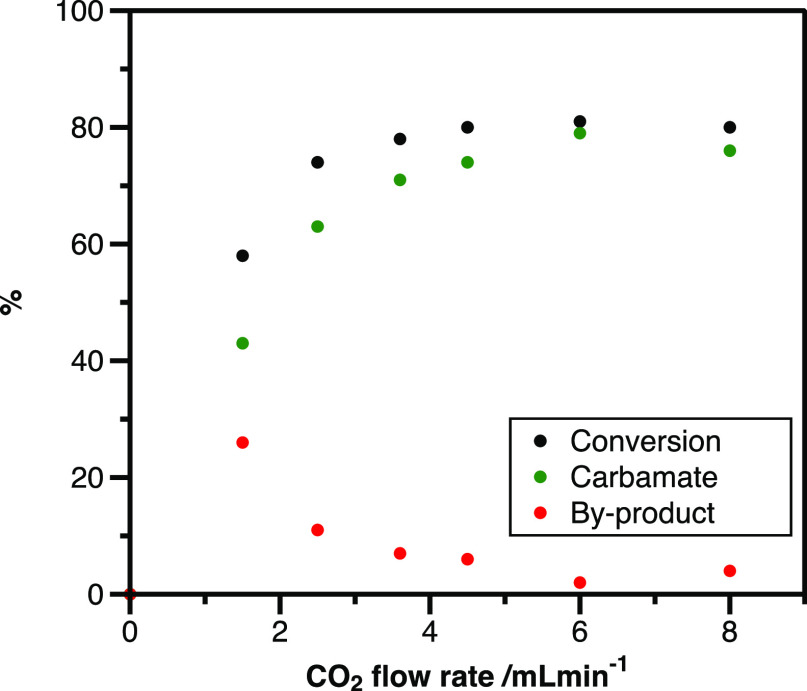
Influence of the CO_2_ Flow Rate on Conversion and Byproduct Formation

First, we sought to investigate the influence of the CO_2_ flow rate on the conversion (Scheme 2); the samples were analyzed by GC–MS.

A CO_2_ flow rate of 1.5 mL/min yielded 58% conversion, which reached 78% with an increase in the flow rate to 3.6 mL/min, but any further rise did not have a significant influence. However, an increased flow rate of 6.0 mL/min yielded a favorable outcome, as the formed byproduct (*N*-butylaniline) amount decreased significantly. We assume that the sizable volumetric excess of carbon dioxide accelerates the formation of the desired carbamate instead of the *N*-alkylated byproduct. Therefore, a CO_2_ flow rate of 6.0 mL/min was chosen for the following experiments.

Subsequently, the reaction mixture flow rate was varied. Neither its decrease nor its increase had any influence on the reaction. In fact, a flow rate of 125 μL/min led to a minor increase in the byproduct amount. Similarly, varying the concentration of the substrate did not affect the conversion.

Further parameters, such as the temperature and pressure, were evaluated after determining the ideal volumetric ratio of the reaction mixture and carbon dioxide (Table 1).

**Table 1:** Effect of Temperature and Pressure on the Conversion

entry^a^	temperature /°C	pressure/bar	conversion /%^b^	carbamate /%^b^	byproduct /%^b^
1	60	3	70	67	3
2	70	3	83	81	2
3	80	3	88	79	9
4	70	1	56	51	5
5	70	5	98	91	7
6	70	7	96	83	13

a Performed with 4.3 mmol (1.0 equiv) aniline, 8.6 mmol (2.0 equiv) DBU, and 8.6 mmol (2.0 equiv) butyl bromide in 5 mL MeCN in a 10 mL coil reactor. Reaction mixture flow rate: 250 μL/min, CO_2_ flow rate: 6.0 mL/min. The product was collected for 50 min.

b Determined by GC–MS analysis.

As can be seen, lowering the temperature to 60 °C had an undesirable outcome, as the conversion decreased by almost 15% (entry 1). On the other hand, an elevated temperature of 80 °C favored the *N*-alkylated byproduct formation (entry 3). Similarly, when maintaining the temperature at 70 °C, a decrease in pressure led to a drop in the conversion by 27% (entry 4), whereas its rise to 5 or 7 bar yielded 7% (entry 5) and 13% (entry 6) byproduct, respectively. Presumably, in the first step of the reaction, CO_2_ is attacked by the amine nucleophile, leading to the formation of a carbamate anion, which attacks the alkyl halide to form the alkyl carbamate.^15,26^

**Scheme 3:**
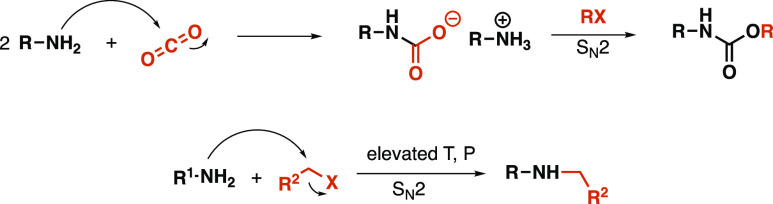
Proposed Mechanism of Carbamate and Byproduct Formation

Strong organic bases are known to stabilize the carbamate intermediate.^15,24^ Supposedly, if the reactor operates at harsher conditions when the reaction mixture enters, the *N*-alkylated byproduct formation is significantly faster than the formation of the carbamate anion. Acetonitrile’s polar and aprotic nature might also favor the S_N_2 substitution of the halide, leading to the formation of *N*-butylaniline (Scheme 3).

Based on these results, 70 °C and 3 bar provide good conversion with only negligible formation of the corresponding byproduct.

After identifying the optimal temperature and pressure, we investigated the influence of the alkyl halide and the DBU on the reaction (Table 2). First, the DBU amount was varied while the halide amount was maintained constant (entries 1–5), and an excess of 2.0 equiv yielded the best conversion (entry 3). Any further increase did not positively affect the reaction. After that, we examined the alkyl halide’s influence on the reaction (entries 6–9). Using 2.5 equiv yielded 91% conversion (entry 8); however, an excess above 2.0 equiv can significantly increase the byproduct amount, as we discovered by testing other nucleophiles.

**Table 2:** Influence of the Alkyl Halide on the Reaction

entry^a^	BuBr eq	DBU eq	conversion /%^b^	carbamate /%^b^
1	2.0	1.0	62	55
2	2.0	1.5	76	69
3	2.0	2.0	81	79
4	2.0	2.5	81	76
5	2.0	3.0	77	73
6	1.0	2.0	59	57
7	1.5	2.0	71	66
8	2.5	2.0	91	87
9	3.0	2.0	87	84

a Performed with 4.3 mmol (1.0 equiv) aniline and 8.6 mmol (2.0 equiv) DBU in 5 mL MeCN in a 10 mL coil reactor. Reaction mixture flow rate: 250 μL/min, CO_2_ flow rate: 6.0 mL/min. The product was collected for 50 min.

b Determined by GC–MS analysis.

Having these optimal conditions at hand, we sought to screen different alkylating agents and additional alkyl bromides. The results are presented in Table 3.

**Table 3:**
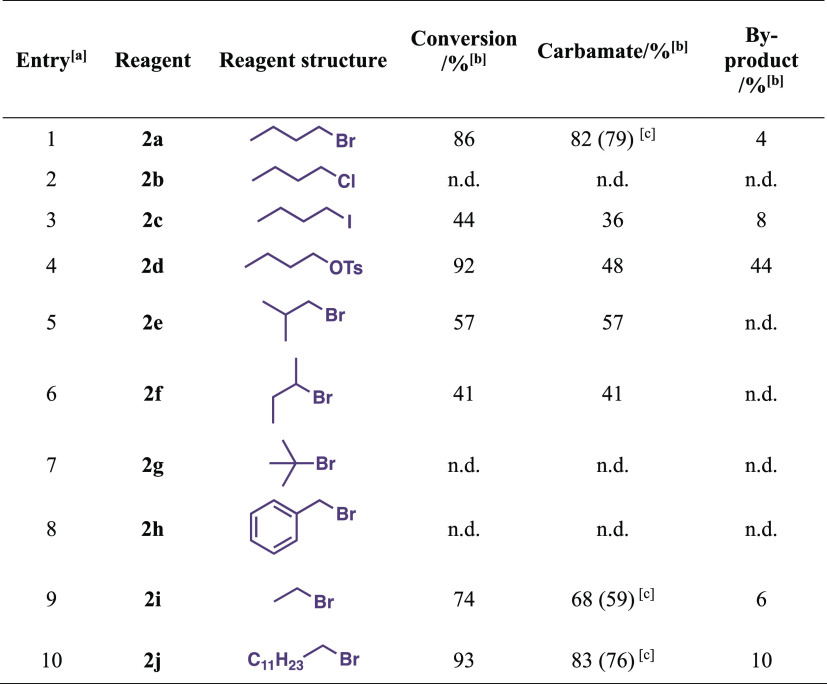
Screening Results for Alkylating Agents

a Performed with 4.3 mmol (1.0 equiv) aniline, 8.6 mmol (2.0 equiv) alkylating agent, and 8.6 mmol (2.0 equiv) DBU in 5 mL MeCN in a 10 mL coil reactor. Reaction mixture flow rate: 250 μL/min, CO_2_ flow rate: 6.0 mL/min. The product was collected for 50 min.

b Determined by GC–MS analysis.

c Isolated yields.

Surprisingly, the employment of 1-iodobutane (entry 3) did not provide sufficient conversion and demonstrated an overall lower reactivity compared to 1-bromobutane (entry 1). 1-Chlorobutane (entry 2) as the alkylating agent proved completely inactive under the developed conditions. When the product was worked with butyl tosylate (entry 4), 48% of the carbamate product was obtained. However, the byproduct amount almost reached the same value (44%). Isobutyl bromide (entry 5) and *sec*-butyl bromide (entry 6) yielded low conversions, 57 and 41%, respectively. Theoretically, these primary and secondary bromides should undergo S_N_2 substitution under these reaction conditions, but the steric hindrance of these halides complicates the reaction. The unsuccessful substitution of *tert*-butyl bromide can be explained by its tertiary nature, which would favor protic polar solvents to undergo S_N_1 substitution. Its inactivity can also be explained by steric hindrance. By adding benzyl bromide (entry 8) to the vial containing the substrate and DBU in acetonitrile, we observed an immediate increase in temperature, indicating that instant *N*-alkylation probably occurred. Ethyl bromide (entry 9) provided a slightly worse conversion, whereas dodecyl bromide (entry 10) proved almost as efficient as butyl bromide.

**Scheme 4:**
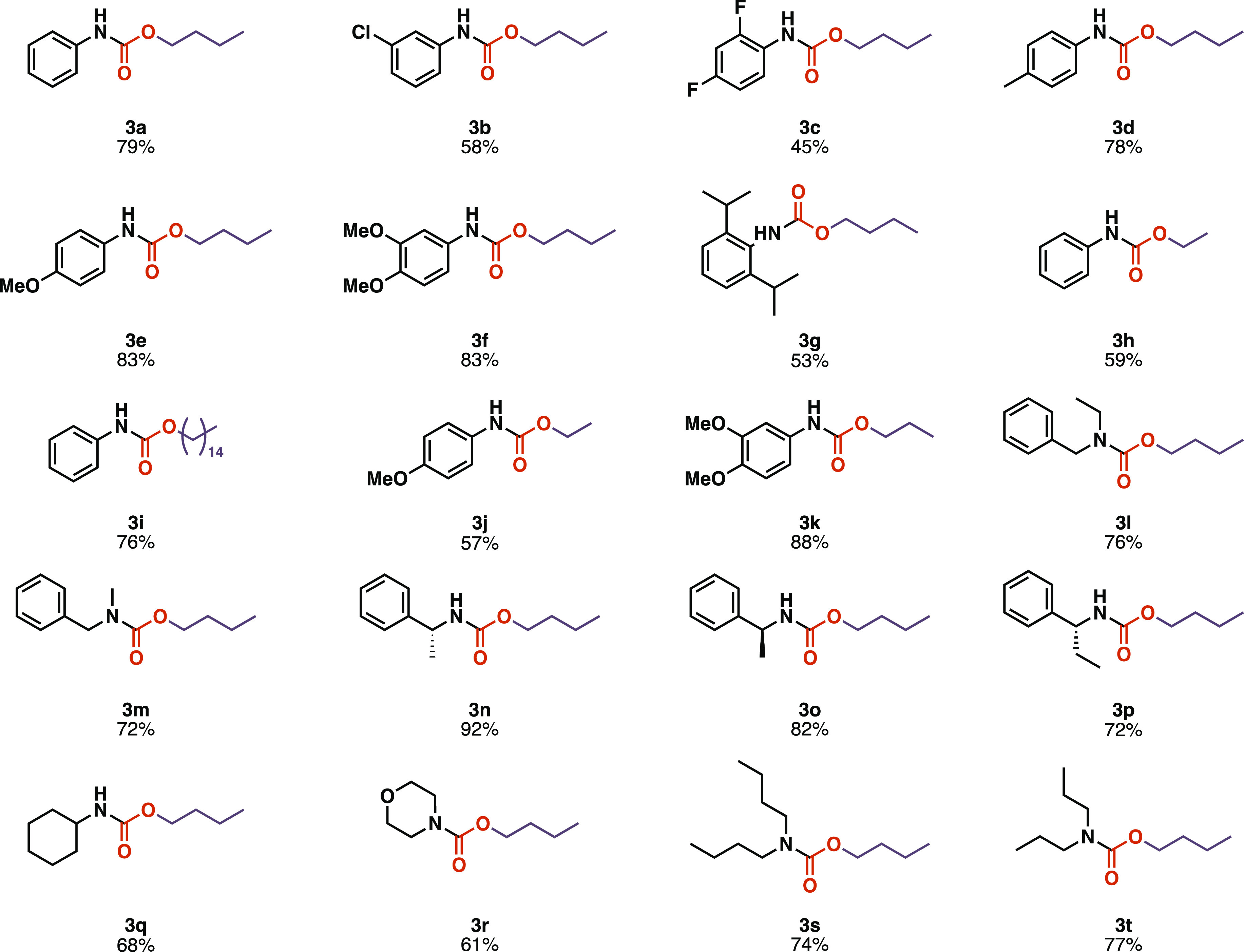
Scope of the Continuous Carbamate Synthesis

After testing a series of alkylating agents, we investigated various amines to extend the scope of our developed method; the results are presented below (Scheme 4).

Aniline derivatives bearing electron-withdrawing groups, such as chloro and fluoro substituents, as well as sterically hindered derivatives, proved less reactive, as the corresponding carbamates (**3b**, **3c**, and **3g**) were isolated with lower yields. Anilines substituted by electron-donating groups provided similar reactivities to the unsubstituted aniline, as the corresponding carbamates (**3d**, **3e**, **3f**, and **3k**) were isolated with good yields. The cyclic secondary amine derivative **3r** was obtained in a slightly lower yield than other noncyclic secondary amine derivatives (**3s** and **3t**). To exclude the possibility of racemization, chiral HPLC analysis was performed in the case of compounds **3n** and **3o** (Supporting Information, Figures S57–S59), which confirmed that the stereocenter remained intact during the reaction.

**Scheme 5:**
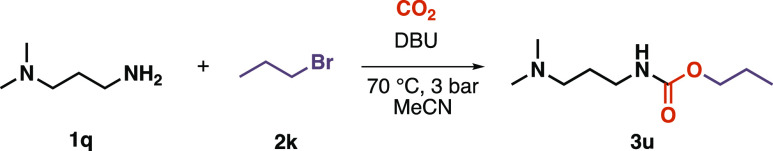
Synthesis of Propamocarb with Our Developed Approach

After successfully expanding the scope of our reaction, we employed our newly developed approach for practical application. The synthesis of a pesticide commercially known as propamocarb (**3u**) was investigated (Scheme 5).

**Scheme 6:**
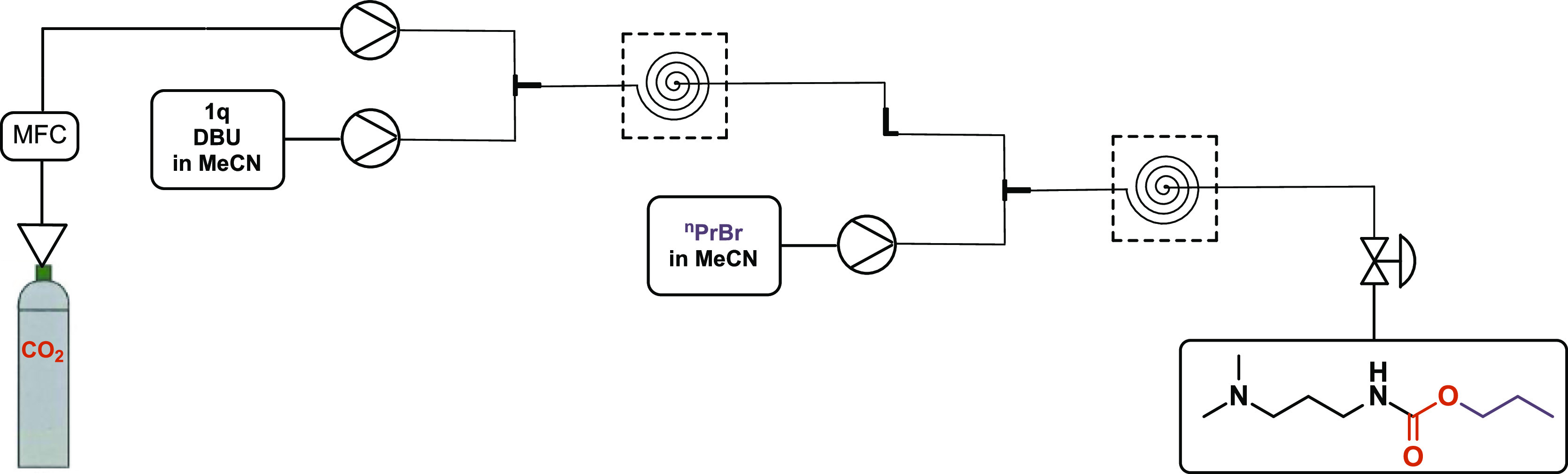
System Setup for Continuous Synthesis of Propamocarb

We initially observed the formation of *N*-alkylated carbamate as the main product while applying the previously developed conditions. Hence, a second alkylation after forming the desired compound **3u** is unlikely; we suppose that the substrate initially reacted with the alkylating agent forming a secondary amine, which further reacted with CO_2_ to form the undesired byproduct. Therefore, we employed two reactors; in the first one, the reaction between the substrate and CO_2_ took place to form the ionic intermediate and this was further reacted in the second coil reactor with a solution of 1-bromopropane in acetonitrile to form the desired product (Scheme 6).

GC–MS analysis of the crude sample indicated complete conversion of propamocarb; however, it should be noted that the separation of the remaining byproducts was not successful with conventional chromatographic methods (Figures S46–S47, Supporting Information).

Finally, we expanded the developed method for the synthesis of oxazolidinones from aziridines. After testing several catalysts reported in the literature (Table 4),^30^ we identified the Lewis acidic tetrabromoferrate ionic liquid formed from tetrabutylammonium bromide (TBAB) and FeBr_3_ as the most suitable catalyst for continuous-flow formation of oxazolidinones (entry 8).^31^

**Table 4:** Toward the Continuous Synthesis of Oxazolidinones

entry	catalyst/conditions	CO_2_ flow rate/mL min–^1^	conversion /%^a^	oxazolidinone /%^a^	byproduct/%^a^
1	10% l-threonine, 110 °C, 0.86 M	6	n.d	n.d	n.d
2	10% TPPH_2_Cl_2_, 70 °C, 0.86 M	6	n.d	n.d	n.d
3	10% TBAB, 70 °C, 0.86 M	6	98	3	95
4	10% [TBA][FeBr_4_], 70 °C, 0.86 M	6	1	1	n.d
5	10% [TBA][FeBr_4_], 50 °C, 0.43 M	8	78	36	42
6	10% [TBA][FeBr_4_], 30 °C, 0.15 M	8	96	63	33
7	10% [TBA][FeBr_4_], 25 °C, 0.15 M	8	88	62	26
8	20% [TBA][FeBr_4_], 25 °C, 0.15 M	8	>99	95	5

a Determined by GC–MS analysis.

**Scheme 7:**
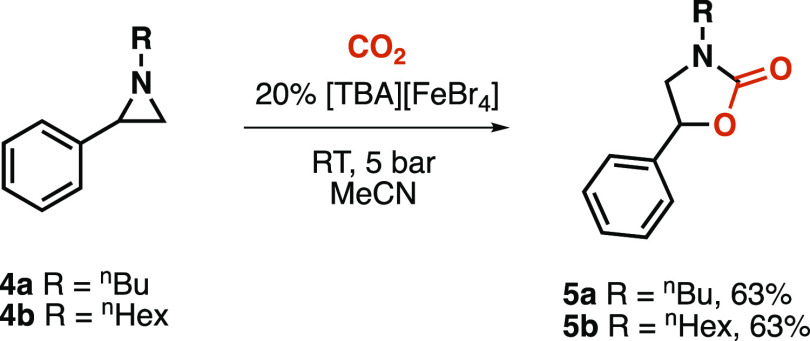
Continuous Synthesis of Oxazolidinones from Aziridines

The reaction was carried out at room temperature, and the back-pressure regulator was set to 5 bar. Under these conditions, we successfully isolated the desired compounds in acceptable yields (63%, Scheme 7).

It is worth mentioning that this method demonstrated high selectivity, as we only observed marginal amounts of the corresponding piperazine dimer byproducts. The isolated oxazolidinones were found analytically pure, and the phenyl group position was confirmed by HSQC NMR analysis (Figure S54, Supporting Information).

## Conclusions

Herein, we report an approach for continuously utilizing CO_2_ in the synthesis of carbamates without employing any catalyst or additive. To the best of our knowledge, this is the first continuous methodology that employs amines and alkyl halides in the presence of DBU and CO_2_ to form urethanes. The process provides a faster and safer alternative for synthesizing carbamates from both primary and secondary amines. The desired compounds were obtained in just 50 min, with good to excellent yields, rendering our method a faster alternative for synthesizing urethanes from CO_2_. Column chromatography could be avoided in many cases since an acidic treatment proved sufficient to obtain the products in high purities. We successfully demonstrated the method’s applicability in synthesizing a commercial pesticide, propamocarb, providing excellent conversion. Moreover, the modified method was tested in the aziridine-based synthesis of oxazolidinones and demonstrated high selectivity toward the desired compounds.

## Experimental Section

### General Procedure for the Continuous-Flow Synthesis of Carbamates

The continuous-flow experiments were performed with the aid of a Vapourtec E-series flow chemistry device in a 10 mL coil reactor. A 30 mL vial with septum was charged with the corresponding amine (1.0 equiv, 4.29 mmol), the corresponding alkyl bromide (2.0 equiv, 8.58 mmol), and DBU (2.0 equiv, 8.58 mmol). The reactants were dissolved in 5 mL acetonitrile. The solvent bottle was charged with MeCN. The reactor was heated to the desired temperature (70 °C). Pump A was used as a back-pressure regulator (BPR = 3 bar). Pump B was connected to the vial with the reaction mixture; pump C was connected to the gas tube, where the CO_2_ was introduced. Carbon dioxide was supplied by a cylinder. The gas flow rate was adjusted with a mass flow controller (6.0 mL/min). The tubes were primed with the reagent mixture and acetonitrile, respectively. The reactor (10 mL coil reactor) was initially rinsed by a CO_2_/MeCN flow for several minutes. Then, the reaction mixture was supplied to the reactor (pump B, 0.25 mL/min; pump C, 6.0 mL/min). After the whole volume of the reaction mixture was pumped through the reactor, the vial was rinsed with pure MeCN, and the residue was pumped through the reactor. The product was collected for 50 min. Rotary evaporation of the solvent gave the crude product, which was bound to silica and subjected to column chromatography. Alternatively, the products could be purified via acidic treatment: the crude residue was taken up in dichloromethane, washed thrice with 1.5 M HCl solution, dried over anhydrous Na_2_SO_4_, filtered, and concentrated.

## Supporting Information Available

Detailed characterization of the synthesized carbamates via ^1^H-, ^13^C-, and ^19^F NMR spectroscopy, infrared spectroscopy, and high-resolution mass spectrometry (PDF)
