# Supplementary material for: Continuous Synthesis of Carbamates from CO2 and Amines
Source: ACS Omega. 2023 Dec 5;8(50):48444–50. doi: 10.1021/acsomega.3c08248 (PMC10734002; doi:10.1021/acsomega.3c08248)
Supplement: Supplementary file 1 — ao3c08248_si_001.pdf [file ao3c08248_si_001.pdf]

Supporting Information

Continuous Synthesis of Carbamates from CO_2 and Amines

Kristof Stagel,[a] *Laura Ielo,*[a,b] *and Katharina Bica-Schröder*[a,*]

[a]Institute of Applied Synthetic Chemistry, TU Wien, Getreidemarkt 9/163, Vienna, 1060, Austria

[b]Department of Chemistry, University of Turin, Via P. Giuria 7, Torino, 10125, Italy

*Corresponding author: Katharina Bica-Schröder. E-mail: katharina.schroeder@tuwien.ac.at, Tel.: +43 1 58801 163601

Table of Contents

1. **General remarks** **2**
2. **Set-up of the continuous-flow experiments** **3**
3. **General procedure for the continuous synthesis of carbamates** **4**
4. **General procedure for the synthesis of aziridines** **10**
5. **General procedure for the continuous synthesis of oxazolidinones** **12**
6. **NMR spectra of 3a-5b** **14**
7. **Chiral HPLC traces** **40**
8. **References** **43**

1. General remarks

Unless otherwise noted, all chemicals purchased from commercial suppliers were used without further purification. Petroleum ether is 40-60 b.p. unless stated otherwise.

Column chromatography was performed on standard glass columns using either Merck (40-60 µm) silica gel or Thermo Scientific (58 Å) neutral aluminum oxide with pre-distilled solvents. Analytical thin-layer chromatography (TLC) was performed on pre-coated, aluminum-backed plates (Merck, silica gel 60 F_{254} or Merck, aluminum oxide 60 F_{254} neutral). All compounds were visualized at 254 nm unless otherwise mentioned.

^{1}H-, ^{13}C- and ^{19}F spectra were recorded from $CDCl_3$ solutions on a Bruker Avance UltraShield 400 MHz (^{1}H: 400 MHz, ^{13}C: 101 MHz, ^{19}F: 376 MHz) NMR instrument. Chemical shifts are reported in parts per million (ppm) from Me_4Si and were calibrated to the residual solvent signal (e.g., $CDCl_3$, ^{1}H: 7.26 ppm, ^{13}C: 77.0 ppm). Coupling constants are reported in hertz (Hz). The assignments are based on comparison with reported spectra.

High-resolution mass spectrometry (HRMS) was carried out using an Agilent 1100/1200 HPLC with a 6230 AJS ESI-TOF MS. GC-MS was carried out using a Thermo Scientific DSQ II with a BGB5 column.

Infrared (IR) spectra were recorded with the aid of a Perkin-Elmer Spectrum65 FT IS spectrometer with absorption maxima (ν_{max}) quoted in wavenumbers (cm^{-1}).

Continuous-flow experiments were performed using a Vapourtec® E-Series flow chemistry device using a standard 10-mL coil reactor.

2. Set-up of the continuous-flow experiments

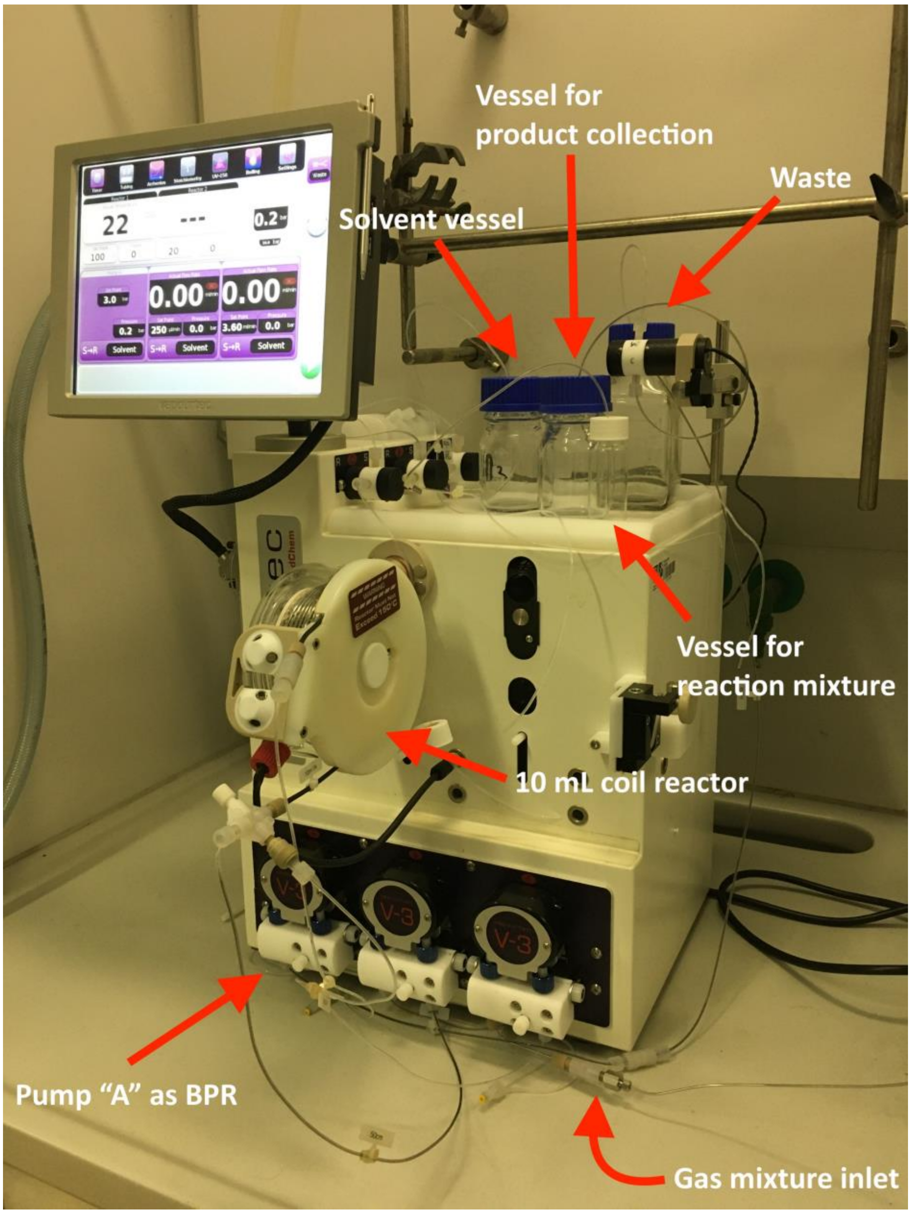

Figure S1. Set-up of the device in continuous-mode operation

3. General procedure for the continuous synthesis of carbamates

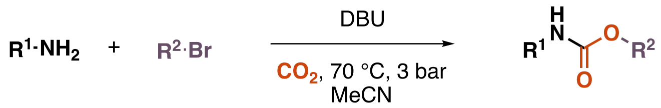

1a R^1 = Ph
1b R^1 = 3-Cl-Ph
1c R^1 = 2,4-difluoro-Ph
1d R^1 = 4-Me-Ph
1e R^1 = 4-OMe-Ph
1f R^1 = 3,4-dimethoxy-Ph
1g R^1 = 2,6-diisopropyl-Ph
1h R^1NH_2 = *N*-benzylethylamine
1i R^1NH_2 = *N*-benzylmethylamine
1j R^1NH_2 = (*R*)-phenylethan-1-amine
1k R^1NH_2 = (*S*)-phenylethan-1-amine
1l R^1NH_2 = (*R*)-phenylpropan-1-amine
1m R^1 = ^{c}Hex
1n R^1NH_2 = morpholine
1o R^1NH_2 = dipropylamine
1p R^1NH_2 = dibutylamine
1q R^1 = *N*,*N*-dimethylaminopropyl

2a R^2 = ^{n}Bu
2i R^2 = Et
2j R^2 = Dod
2k R^2 = ^{n}Pr

3a R^1 = Ph, R^2 = ^{n}Bu
3b R^1 = 3-Cl-Ph, R^2 = ^{n}Bu
3c R^1 = 2,4-difluoro-Ph, R^2 = ^{n}Bu
3d R^1 = 4-Me-Ph, R^2 = ^{n}Bu
3e R^1 = 4-OMe-Ph, R^2 = ^{n}Bu
3f R^1 = 3,4-dimethoxy-Ph, R^2 = ^{n}Bu
3g R^1 = 2,6-diisopropyl-Ph, R^2 = ^{n}Bu
3h R^1 = Ph, R^2 = Et
3i R^1 = Ph, R^2 = ^{n}Dod
3j R^1 = 4-MeO-Ph, R^2 = Et
3k R^1 = 3,4-dimethoxy-Ph, R^2 = ^{n}Pr
3l R^1NH = *N*-benzylethylamine, R^2 = ^{n}Bu
3m R^1NH = *N*-benzylmethylamine, R^2 = ^{n}Bu
3n R^1NH = (*R*)-phenylethan-1-amine, R^2 = ^{n}Bu
3o R^1NH = (*S*)-phenylethan-1-amine, R^2 = ^{n}Bu
3p R^1NH = (*R*)-phenylpropan-1-amine, R^2 = ^{n}Bu
3q R^1 = ^{c}Hex, R^2 = ^{n}Bu
3r R^1NH = morpholine, R^2 = ^{n}Bu
3s R^1NH = dipropylamine, R^2 = ^{n}Bu
3t R^1NH = dibutylamine, R^2 = ^{n}Bu
3u R^1= *N*,*N*-dimethylaminopropyl, R^2 = ^{n}Pr

Figure S2. Reaction for the continuous synthesis of carbamates with various starting materials

A 30-mL vial with septum was charged with the corresponding amine (1.0 eq., 4.29 mmol), the corresponding alkyl bromide (2.0 eq., 8.58 mmol), and DBU (2.0 eq., 8.58 mmol). The reactants were dissolved in 5 mL acetonitrile. The solvent bottle was charged with MeCN. The reactor was heated up to the desired temperature (70 °C). Pump A was used as a back-pressure regulator (BPR, 3 bar). Pump B was connected to the vial with the reaction mixture; pump C was connected to the gas tube, where the CO_2 was introduced. Carbon dioxide was supplied from a gas cylinder. The gas flow rate was set with a mass flow controller (6 mL/min). The tubes were primed with the reagent mixture and acetonitrile, respectively. The reactor (10-mL coil reactor) was initially rinsed by a CO_2/MeCN flow for several minutes. Then, the reaction mixture was supplied to the reactor (pump B: 0.25 mL/min; pump C: 6 mL/min). After the entire volume of the reaction mixture was pumped through the reactor, the vial was rinsed with pure MeCN, and the residue was pumped through the reactor. The product was collected for 50 minutes. Following rotary evaporation of the solvent the crude product was recovered, which was bound to silica and, subsequently, subjected to column chromatography on silica gel. Alternatively, the products could be purified *via* acidic treatment: the crude residue was taken up in dichloromethane, washed thrice with 1.5 M HCl solution, dried over anhydrous Na_2SO_4, filtered, and concentrated.

Butyl phenylcarbamate (3a)[1]

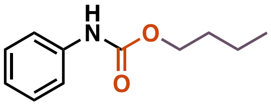

Acidic treatment afforded the product as slightly yellowish white solid (657 mg, 3.4 mmol, 79%). **^{1}H NMR** (400 MHz, $CDCl_3$) δ 7.38 (d, J = 8.0 Hz, 2H), 7.34 – 7.27 (m, 2H), 7.12 – 7.00 (m, 1H), 6.63 (s, 1H), 4.17 (t, J = 6.7 Hz, 2H), 1.76 – 1.56 (m, 2H), 1.54 – 1.32 (m, 2H), 0.96 (t, J = 7.4 Hz, 3H). **^{13}C NMR** (101 MHz, $CDCl_3$) δ 153.87, 138.12, 129.17, 123.46, 118.76, 65.26, 31.11, 19.21, 13.86.

Butyl (3-chlorophenyl)carbamate (3b)[2]

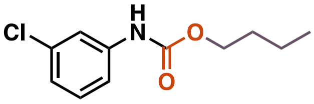

Acidic treatment afforded the product as slightly orange oil (564 mg, 2.5 mmol, 58%). **^{1}H NMR** (400 MHz, $CDCl_3$) δ 7.59 – 7.43 (m, 1H), 7.24 – 7.14 (m, 2H), 7.10 – 6.98 (m, 1H), 6.60 (s, 1H), 4.15 (dt, J = 17.3, 6.7 Hz, 2H), 1.70 – 1.61 (m, 2H), 1.48 – 1.36 (m, 2H), 0.96 (t, J = 7.4 Hz, 3H). **^{13}C NMR** (101 MHz, $CDCl_3$) δ 153.53, 139.34, 134.92, 130.15, 123.50, 118.77, 116.65, 65.53, 31.05, 19.20, 13.85.

Butyl (2,4-difluorophenyl)carbamate (3c)

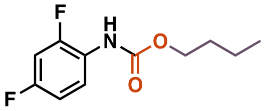

Flash column chromatography (petroleum ether/Et_2O 10:1, $\boldsymbol{R}_\mathbf{f}$ = 0.44) afforded the product as colourless oil (440 mg, 1.9 mmol, 45%). **^{1}H NMR** (400 MHz, $CDCl_3$) δ 8.02 (s, 1H), 6.85 (qt, J = 8.8, 2.8 Hz, 2H), 6.68 (s, 1H), 4.18 (t, J = 6.7 Hz, 2H), 1.66 (ddt, J = 8.8, 8.0, 6.5 Hz, 2H), 1.51 – 1.32 (m, 2H), 0.96 (t, J = 7.4 Hz, 3H). **^{13}C NMR** (101 MHz, $CDCl_3$) δ 153.53, 139.34, 134.92, 130.15, 123.50, 118.77, 116.65, 65.53, 31.05, 19.20, 13.85. **^{19}F NMR** (376 MHz, $CDCl_3$) δ -116.99, -127.95. **HRMS (ESI):** calcd. for $C_{11}H_{14}F_2NO_2$ $[M+H]^+$: 230.987; found: 230.0989. **IR** ν_{max}/ cm^{-1}: 2961, 1717, 1612, 1529, 1432, 1287, 1222, 1194, 1141, 1099, 1068, 962, 846, 727.

Butyl *p*-tolylcarbamate (3d)[3]

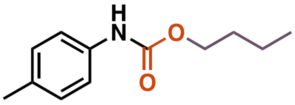

Acidic treatment afforded the product as yellowish brown oil (689 mg, 3.3 mmol, 78%). **^{1}H NMR** (400 MHz, CDCl3) δ 7.26 (d, J = 8.2 Hz, 2H), 7.10 (d, J = 8.2 Hz, 2H), 6.53 (s, 1H), 4.16 (t, J = 6.7 Hz, 2H), 2.30 (s, 3H), 1.65 (ddt, J = 8.8, 7.8, 6.5 Hz, 2H), 1.48 – 1.32 (m, 2H), 0.95 (t, J = 7.4 Hz, 3H). **^{13}C NMR** (101 MHz, CDCl3) δ 153.98, 135.51, 133.03, 129.66, 118.66, 65.18, 31.13, 20.87, 19.23, 13.87.

Butyl (4-methoxyphenyl)carbamate (3e)[4]

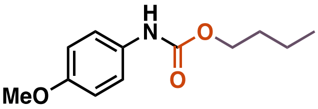

Acidic treatment afforded the product as black oil (797 mg, 3.6 mmol, 83%). **^{1}H NMR** (400 MHz, $CDCl_3$) δ 7.28 (d, J = 8.3 Hz, 2H), 6.90 – 6.79 (m, 2H), 6.49 (s, 1H), 4.15 (t, J = 6.7 Hz, 2H), 3.78 (s, 3H), 1.64 (ddt, J = 8.9, 8.0, 6.5 Hz, 2H), 1.50 – 1.34 (m, 2H), 0.95 (t, J = 7.4 Hz, 3H). **^{13}C**

NMR (101 MHz, $CDCl_3$) δ 156.05, 154.26, 131.18, 120.75, 114.37, 65.17, 55.64, 31.14, 19.22, 13.87.

Butyl (3,4-dimethoxyphenyl)carbamate (3f)

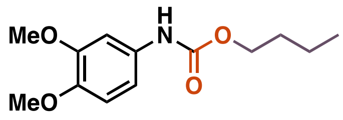

Acidic treatment afforded the product as black solid (897 mg, 3.5 mmol, 83%). **M.p.** 56-58 °C. **^{1}H NMR** (400 MHz, $CDCl_3$) δ 7.19 (s, 1H), 6.83 – 6.70 (m, 2H), 6.50 (s, 1H), 4.15 (t, *J* = 6.7 Hz, 2H), 3.86 (dd, *J* = 11.4, 0.9 Hz, 6H), 1.65 (dtd, *J* = 8.3, 6.9, 5.9 Hz, 2H), 1.47 – 1.32 (m, 2H), 0.99 – 0.88 (m, 3H). **^{13}C NMR** (101 MHz, $CDCl_3$) δ 154.14, 149.37, 145.43, 131.72, 111.67, 65.20, 56.31, 56.00, 31.13, 19.23, 13.86. **HRMS (ESI):** calcd. for $C_{13}H_{20}NO_4$ $[M+H]^+$: 254.1387; found: 254.1388. **IR** ν_{max}/cm^{-1}: 2959, 1689, 1604,1534, 1455, 1419, 1290, 1267, 1230, 1171, 1138, 1075, 1023, 956, 848, 805, 756, 737.

Butyl (2,6-diisopropylphenyl)carbamate (3g)

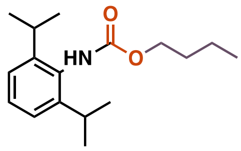

Flash column chromatography (petroleum ether/Et_2O 10:1, ***R*f** = 0.28) afforded the product as white solid (636 mg, 2.3 mmol, 53%). **M.p.** 76-79 °C. **^{1}H NMR** (400 MHz, $CDCl_3$) δ 7.33 – 7.24 (m, 1H), 7.16 (d, *J* = 7.7 Hz, 2H), 5.93 (s, 1H), 4.12 (dt, *J* = 40.5, 6.8 Hz, 2H), 3.19 (dt, *J* = 14.6, 7.9 Hz, 2H), 1.67 (q, *J* = 7.5 Hz, 2H), 1.45 (dt, *J* = 15.3, 7.6 Hz, 2H), 1.21 (d, *J* = 7.4 Hz, 12H), 1.04 – 0.90 (m, 2H), 0.83 (q, *J* = 6.2, 5.5 Hz, 1H). **^{13}C NMR** (101 MHz, $CDCl_3$) δ 155.74, 146.96, 128.42, 123.59, 65.25, 31.30, 28.74, 24.11, 23.77, 23.34, 19.17, 13.91. **HRMS (ESI):** calcd. for $C_{17}H_{28}NO_2$ $[M+H]^+$: 278.2115; found: 278.2119. **IR** ν_{max}/cm^{-1}: 2959, 1695, 1509, 1474, 1245, 1204, 1074, 1052, 799.

Ethyl phenylcarbamate (3h)[5]

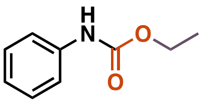

Acidic treatment afforded the product as orange oil (420 mg, 2.5 mmol, 59%). **^{1}H NMR** (400 MHz, $CDCl_3$) δ 7.43 – 7.34 (m, 2H), 7.34 – 7.27 (m, 2H), 7.09 – 7.02 (m, 1H), 6.63 (s, 1H), 4.23 (q, *J* = 7.1 Hz, 2H), 1.31 (t, *J* = 7.1 Hz, 3H). **^{13}C NMR** (101 MHz, $CDCl_3$) δ 153.74, 138.09, 129.17, 123.48, 118.79, 61.34, 14.68.

Tetradecyl phenylcarbamate (3i)[6]

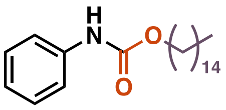

Flash column chromatography (petroleum ether/Et_2O 15:1, ***R*f** = 0.45, *p*-anisaldehyde stain) afforded the product as white solid (997 mg, 3.3 mmol, 76%). **^{1}H NMR** (400 MHz, $CDCl_3$) δ 7.46 – 7.23 (m, 4H), 7.12 – 6.96 (m, 1H), 6.58 (s, 1H), 4.16 (t, *J* = 6.7 Hz, 2H), 1.75 – 1.60 (m, 2H), 1.45 – 1.18 (m, 18H), 0.88 (t, *J* = 6.8 Hz, 3H). **^{13}C NMR** (101 MHz, $CDCl_3$) δ 153.84, 138.12, 129.18, 123.47, 118.75, 65.59, 32.06, 29.79, 29.77, 29.72, 29.69, 29.49, 29.43, 29.09, 26.02, 22.83, 14.26.

Ethyl (4-methoxyphenyl)carbamate (3j)[7]

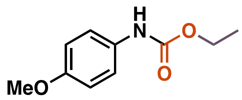

Acidic treatment afforded the product as black solid (475 mg, 2.4 mmol, 57%). **^{1}H NMR** (400 MHz, $CDCl_3$) δ 7.28 (d, *J* = 8.5 Hz, 2H), 6.91 – 6.79 (m, 2H), 6.56 (s, 1H), 4.26 – 4.15 (m, 2H), 3.78 (d, *J* = 1.1 Hz, 3H), 1.29 (tt, *J* = 7.1, 1.2 Hz, 3H). **^{13}C NMR** (101 MHz, $CDCl_3$) δ 156.05, 154.17, 131.16, 120.82, 114.36, 61.23, 55.62, 14.71.

Propyl (3,4-dimethoxyphenyl)carbamate (3k)

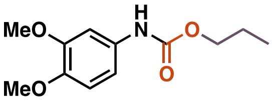

Acidic treatment afforded the product as black solid (900 mg, 3.8 mmol, 88%). **M.p.** 55-58 °C. **^{1}H NMR** (400 MHz, $CDCl_3$) δ 7.19 (s, 1H), 6.75 (dd, *J* = 10.9, 8.5 Hz, 2H), 6.54 (s, 1H), 4.11 (t, *J* = 6.7 Hz, 2H), 3.87 (t, J = 0.8 Hz, 3H), 3.84 (d, *J* = 0.7 Hz, 3H), 1.76 – 1.62 (m, 2H), 0.97 (t, *J* = 7.4 Hz, 3H). **^{13}C NMR** (101 MHz, $CDCl_3$) δ 154.14, 149.35, 145.41, 131.72, 111.66, 66.92, 56.30, 55.99, 22.43, 10.48. **HRMS (ESI):** calcd. for $C_{12}H_{18}NO_4$ $[M+H]^+$: 240.1230; found: 240.1231. **IR** ν_{max}/cm^{-1}: 2966, 1689, 1604, 1516, 1447, 1326, 1291, 1223, 1169, 1137, 1070, 1022, 957, 847, 804, 755.

Butyl benzyl(ethyl)carbamate (3l)

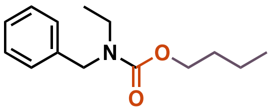

Acidic treatment afforded the product as yellow oil (768 mg, 3.3 mmol, 76%). **^{1}H NMR** (400 MHz, $CDCl_3$) δ 7.40 – 7.02 (m, 5H), 4.47 (s, 2H), 4.13 (t, *J* = 6.5 Hz, 2H), 3.41 – 3.11 (m, 2H), 1.64 (dt, *J* = 16.9, 11.3 Hz, 2H), 1.45 – 1.25 (m, 2H), 1.07 (s, 3H), 1.01 – 0.84 (m, 3H). **^{13}C NMR** (101 MHz, $CDCl_3$) δ 138.42, 128.61, 127.33, 65.38, 31.29, 19.33, 13.90. **HRMS (ESI):** calcd. for $C_{14}H_{22}NO_2$ $[M+H]^+$: 236.1645; found: 236.1647. **IR** ν_{max}/cm^{-1}: 2959, 1694, 1470, 1422, 1256, 1142, 1081, 980, 733.

Butyl benzyl(methyl)carbamate (3m)

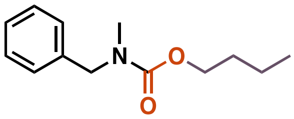

Acidic treatment afforded the product as yellow oil (681 mg, 3.08 mmol, 72%). **^{1}H NMR** (400 MHz, $CDCl_3$) δ 7.42 – 7.15 (m, 5H), 4.47 (s, 2H), 4.13 (t, *J* = 6.6 Hz, 2H), 2.85 (s, 3H), 1.72 – 1.59 (m, 2H), 1.50 – 1.28 (m, 2H), 0.99 – 0.82 (m, 3H). **^{13}C NMR** (101 MHz, $CDCl_3$) δ 137.79, 128.69, 127.44, 65.56, 52.48, 31.28, 19.30, 13.89. **HRMS (ESI):** calcd. for $C_{13}H_{19}NO_2$ $[M+H]^+$: 222.1489; found: 222.1491. **IR** ν_{max}/cm^{-1}: 2959, 1692, 1647, 1620, 1456, 1325, 747.

Butyl (*R*)-(1-phenylethyl)carbamate (3n)[8]

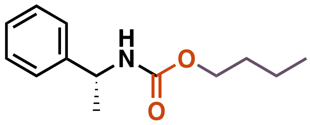

Flash column chromatography (petroleum ether/Et_2O 5:1, $\boldsymbol{R_f}$ = 0.20) afforded the product as slightly yellowish oil (875 mg, 4.0 mmol, 92%). **^{1}H NMR** (400 MHz, $CDCl_3$) δ 7.42 – 7.20 (m, 5H), 4.87 (d, *J* = 22.9 Hz, 2H), 4.04 (td, *J* = 6.7, 4.7 Hz, 2H), 1.56 (q, *J* = 7.4, 6.8 Hz, 2H), 1.48 (d, *J* = 6.7 Hz, 3H), 1.30 (td, *J* = 20.6, 19.9, 6.2 Hz, 2H), 0.92 (dt, *J* = 11.2, 7.4 Hz, 3H). **^{13}C NMR**

(101 MHz, $CDCl_3$) δ 156.11, 128.76, 127.41, 126.06, 64.92, 50.69, 31.19, 22.65, 19.19, 13.87. **Chiral HPLC:** Chiralpak® AS-H column, *n*-hexane: isopropanol 90:10, 1 mL/min, 25 °C, UV 220 nm, t_R = 8.3 min.

Butyl (*S*)-(1-phenylethyl)carbamate (3o)[8]

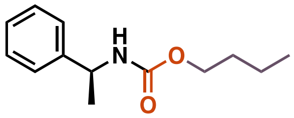

Flash column chromatography (petroleum ether/Et_2O 5:1, R_f = 0.20) afforded the product as slightly yellowish oil (774 mg, 3.5 mmol, 82%). **^{1}H NMR** (400 MHz, $CDCl_3$) δ 7.40 – 7.18 (m, 5H), 4.87 (d, *J* = 22.9 Hz, 2H), 4.04 (td, *J* = 6.7, 4.7 Hz, 2H), 1.56 (q, *J* = 7.4, 6.8 Hz, 2H), 1.48 (d, J = 6.7 Hz, 3H), 1.32 (dq, *J* = 19.2, 12.9, 10.3 Hz, 2H), 0.92 (q, *J* = 7.3, 5.6 Hz, 3H). **^{13}C NMR** (101 MHz, $CDCl_3$) δ 156.11, 128.76, 127.41, 126.06, 64.92, 50.71, 31.19, 22.65, 19.19, 13.87. **Chiral HPLC:** Chiralpak® AS-H column, *n*-hexane: isopropanol 90:10, 1 mL/min, 25 °C, UV 220 nm, t_R = 6.2 min.

Butyl (*R*)-(1-phenylpropyl)carbamate (3p)

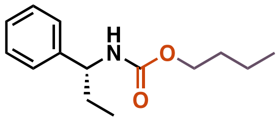

Flash column chromatography (petroleum ether/Et_2O 5:1, R_f = 0.23, $KMnO_4$ stain) afforded the product as slightly yellowish oil (726 mg, 3.09 mmol, 72%). **^{1}H NMR** (400 MHz, $CDCl_3$) δ 7.40 – 7.17 (m, 5H), 4.91 (s, 1H), 4.58 (s, 1H), 4.12 – 3.93 (m, 2H), 1.83 – 1.75 (m, 2H), 1.56 (s, 2H), 1.35 (s, 2H), 0.90 (t, *J* = 7.4 Hz, 6H). **^{13}C NMR** (101 MHz, $CDCl_3$) δ 128.69, 127.37, 126.53, 64.92, 56.88, 31.19, 29.84, 19.19, 13.87, 10.82. **HRMS (ESI):** calcd. for $C_{14}H_{22}NO_2$ $[M+H]^+$: 236.1645; found: 236.1645. **IR** ν_{max}/cm^{-1}: 2961, 1688, 1529, 1455, 1232, 1084, 1041, 756.

Butyl cyclohexylcarbamate (3q)[9]

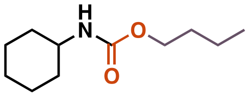

Flash column chromatography (petroleum ether/Et_2O 5:1, R_f = 0.32, $KMnO_4$ stain) afforded the product as white solid (578 mg, 2.9 mmol, 68%). **^{1}H NMR** (400 MHz, $CDCl_3$) δ 4.51 (s, 1H), 4.04 (t, *J* = 6.6 Hz, 2H), 3.46 (s, 1H), 1.93 (dt, *J* = 12.4, 4.2 Hz, 2H), 1.69 (dq, J = 12.0, 3.9 Hz, 2H), 1.64 – 1.48 (m, 3H), 1.45 – 1.25 (m, 4H), 1.21 – 1.03 (m, 3H), 0.92 (t, *J* = 7.4 Hz, 3H). **^{13}C NMR** (101 MHz, $CDCl_3$) δ 156.23, 64.71, 49.94, 33.61, 31.26, 25.64, 24.93, 19.25, 13.90.

Butyl morpholine-4-carboxylate (3r)[10]

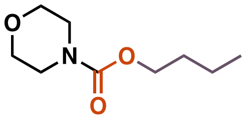

Acidic treatment afforded the product as yellow oil (492 mg, 2.6 mmol, 61%). **^{1}H NMR** (400 MHz, $CDCl_3$) δ 4.09 (t, *J* = 6.6 Hz, 2H), 3.64 (t, *J* = 4.8 Hz, 4H), 3.52 – 3.32 (m, 4H), 1.61 (dq, *J* = 8.5, 6.7 Hz, 2H), 1.45 – 1.29 (m, 2H), 0.93 (t, *J* = 7.4 Hz, 3H). **^{13}C NMR** (101 MHz, $CDCl_3$) δ 155.78, 66.75, 65.57, 44.14, 31.17, 19.30, 13.88.

Butyl dibutylcarbamate (3s)[11]

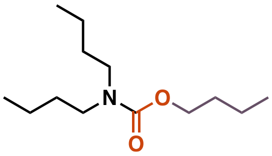

Acidic treatment afforded the product as slightly yellowish oil (725 mg, 3.2 mmol, 74%). **^{1}H NMR** (400 MHz, $CDCl_3$) δ 4.05 (t, *J* = 6.6 Hz, 2H), 3.18 (s, 4H), 1.60 (ddt, *J* = 8.7, 7.9, 6.4 Hz, 2H), 1.49 (p, *J* = 7.5 Hz, 4H), 1.43 – 1.36 (m, 2H), 1.28 (dt, *J* = 14.7, 7.4 Hz, 4H), 0.92 (q, *J* = 7.2 Hz, 9H). **^{13}C NMR** (101 MHz, $CDCl_3$) δ 156.71, 64.95, 46.99, 31.31, 30.71, 20.17, 19.38, 14.00.

Butyl dipropylcarbamate (3t)

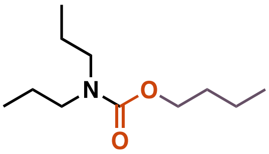

Acidic treatment afforded the product as yellow oil (665 mg, 3.3 mmol, 77%). **^{1}H NMR** (400 MHz, $CDCl_3$) δ 4.05 (t, *J* = 6.6 Hz, 2H), 3.19 – 3.11 (m, 4H), 1.65 – 1.46 (m, 6H), 1.43 – 1.32 (m, 2H), 0.93 (t, *J* = 7.4 Hz, 3H), 0.86 (t, *J* = 7.4 Hz, 6H). **^{13}C NMR** (101 MHz, $CDCl_3$) δ 156.78, 64.96, 48.99, 31.31, 21.74, 19.37, 13.90, 11.37. **HRMS (ESI):** calcd. for $C_{11}H_{24}NO_2$ $[M+H]^+$: 202.1802; found: 202.1803. **IR** ν_{max}/cm^{-1}: 2959, 1695, 1509, 1474, 1245, 1204, 1074, 1052, 799.

4. General procedure for the synthesis of aziridines

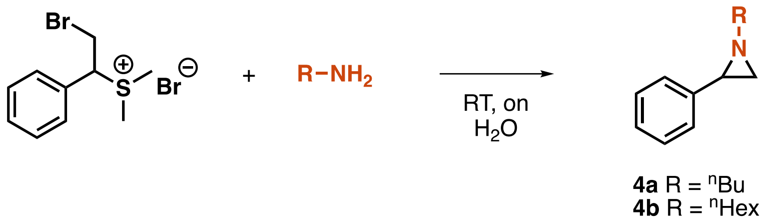

Figure S3. Synthesis of aziridines

(2-bromo-1-phenylethyl)dimethylsulfonium bromide was prepared according to the standard literature procedure.[12] Aziridines were prepared according to the literature procedure;[13] the alkylsulfonium bromide (1.0 eq., 10 mmol) was dissolved in 20 mL distilled water. To this, the solution of the corresponding amine (3.0 eq., 30 mmol) in 10 mL water was added dropwise. The reaction mixture was stirred overnight at room temperature. After completion, the mixture was quenched with 20 mL brine, extracted with diethyl ether (3 x 20 mL), dried over anhydrous Na_2SO_4, and concentrated *in vacuo*. The crude products were subjected to column chromatography on neutral aluminum oxide (Brockmann Grade IV).

1-butyl-2-phenylaziridine (4a)[14]

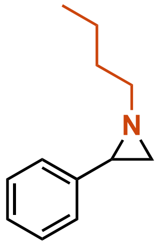

Column chromatography (petroleum ether/Et_2O 20:1, $\boldsymbol{R}_\mathbf{f}$ = 0.61) afforded the product as colorless liquid (1.56 g, 8.9 mmol, 89%). **^{1}H NMR** (400 MHz, $CDCl_3$) δ 7.35 – 7.18 (m, 5H), 2.51 (dt, J = 11.5, 7.3 Hz, 1H), 2.38 – 2.27 (m, 2H), 1.89 (dd, J = 3.4, 0.8 Hz, 1H), 1.68 – 1.64 (m, 1H), 1.64 – 1.55 (m, 2H), 1.47 – 1.33 (m, 2H), 0.93 (t, J = 7.3 Hz, 3H). **^{13}C NMR** (101 MHz, $CDCl_3$) δ 140.70, 128.38, 126.87, 126.31, 61.71, 41.39, 37.92, 32.13, 20.74, 14.25.

1-hexyl-2-phenylaziridine (4b)[15]

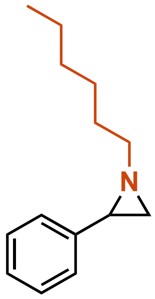

Column chromatography (petroleum ether/Et_2O 20:1, $\boldsymbol{R}_\mathbf{f}$ = 0.69) afforded the product as colorless liquid (1.97 g, 9.7 mmol, 97%). **^{1}HNMR** (400 MHz, $CDCl_3$) δ 7.48 – 7.16 (m, 5H), 2.49 (dt, J = 11.5, 7.4 Hz, 1H), 2.38 – 2.27 (m, 2H), 1.90 (dd, J = 3.3, 0.7 Hz, 1H), 1.69 – 1.55 (m, 3H), 1.44 – 1.22 (m, 6H), 0.97 – 0.81 (m, 3H). **^{13}C NMR** (101 MHz, $CDCl_3$) δ 140.69, 128.38, 126.87, 126.32, 62.05, 41.39, 37.91, 31.96, 29.93, 27.27, 22.73, 14.18.

5. General procedure for the continuous synthesis of oxazolidinones

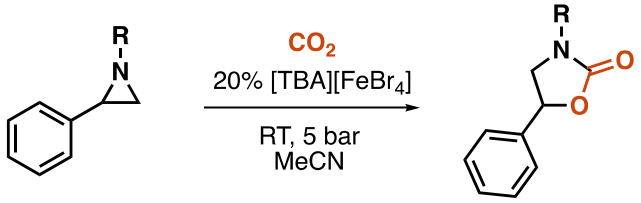

4a R = ^{n}Bu
4b R = ^{n}Hex

5a R = ^{n}Bu
5b R = ^{n}Hex

Figure S4. Synthesis of compounds **5a-b**

A 30-mL vial with septum was charged with the corresponding aziridine (1.0 eq., 0.73 mmol), $FeBr_3$ (20 mol%, 0.146 mmol), and TBAB (20 mol%, 0.146 mmol). The reactants were dissolved in 5 mL acetonitrile. The solvent bottle was charged with MeCN. Pump A was used as a back-pressure regulator (BPR, 5 bar). Pump B was connected to the vial with the reaction mixture; pump C was connected to the gas tube, where the CO_2 was introduced. Carbon dioxide was supplied from a gas cylinder. The gas flow rate was set with a mass flow controller (8 mL/min). The tubes were primed with the reagent mixture and acetonitrile, respectively. The reactor (10-mL coil reactor) was initially rinsed by a CO_2/MeCN flow for several minutes. Then, the reaction mixture was supplied to the reactor (pump B: 0.25 mL/min; pump C: 8 mL/min). After the whole volume of the reaction mixture was pumped through the reactor, the vial was rinsed with pure MeCN, and the residue was pumped through the reactor. The product was collected for 50 minutes. Following rotary evaporation of the solvent the crude product was recovered, which was bound to silica gel and subjected to column chromatography on silica gel.

3-butyl-5-phenyloxazolidin-2-one (5a)[14]

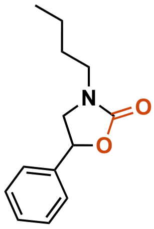

Column chromatography (petroleum ether/Et_2O 2:1, $\boldsymbol{R_f}$ = 0.55) afforded the product as colorless liquid (101 mg, 4.6 mmol, 63%). **^{1}H NMR** (400 MHz, $CDCl_3$) δ 7.46 – 7.29 (m, 5H), 5.48 (t, *J* = 8.1 Hz, 1H), 3.91 (t, *J* = 8.7 Hz, 1H), 3.42 (ddd, *J* = 8.2, 7.4, 0.7 Hz, 1H), 3.39 – 3.21 (m, 2H), 1.60 – 1.47 (m, 2H), 1.43 – 1.29 (m, 2H), 0.94 (t, *J* = 7.3 Hz, 3H). **^{13}C NMR** (101 MHz, $CDCl_3$) δ 158.06, 139.05, 129.04, 128.90, 125.63, 74.45, 52.32, 44.07, 29.56, 19.98, 13.83.

3-hexyl-5-phenyloxazolidin-2-one (5b)[16]

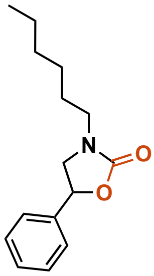

Column chromatography (petroleum ether/Et_2O 2:1, $\boldsymbol{R_f}$ = 0.61) afforded the product as colorless liquid (114 mg, 4.6 mmol, 63%). **^{1}H NMR** (400 MHz, $CDCl_3$) δ 7.49 – 7.28 (m, 5H), 5.48 (dd, *J* = 8.8, 7.4 Hz, 1H), 3.91 (t, *J* = 8.7 Hz, 1H), 3.42 (dd, *J* = 8.6, 7.4 Hz, 1H), 3.30 (ddp, *J* = 21.1, 14.0, 7.1 Hz, 2H), 1.55 (p, *J* = 7.4 Hz, 2H), 1.39 – 1.15 (m, 6H), 0.88 (q, *J* = 4.0, 3.2 Hz, 3H). **^{13}C NMR** (101 MHz, $CDCl_3$) δ 158.03, 139.08, 129.02, 128.89, 125.62, 74.42, 52.31, 44.35, 31.53, 27.46, 26.41, 22.65, 14.11.

6. NMR spectra of 3a-5b

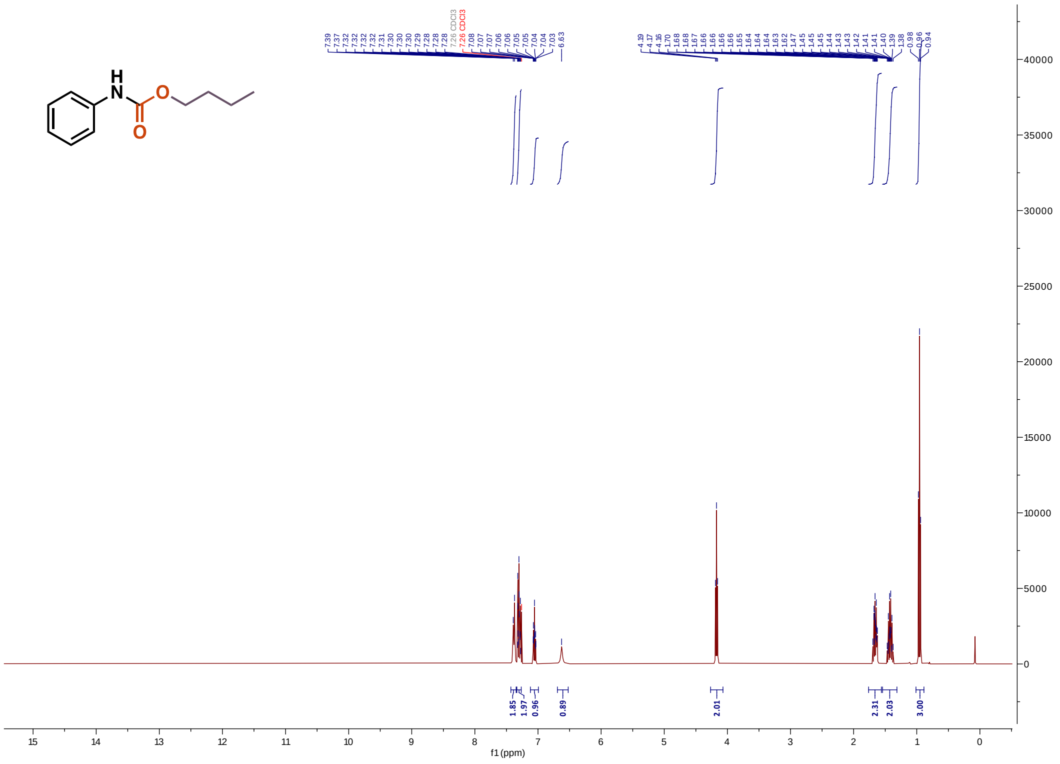

Figure S5. ^{1}H NMR of **3a**

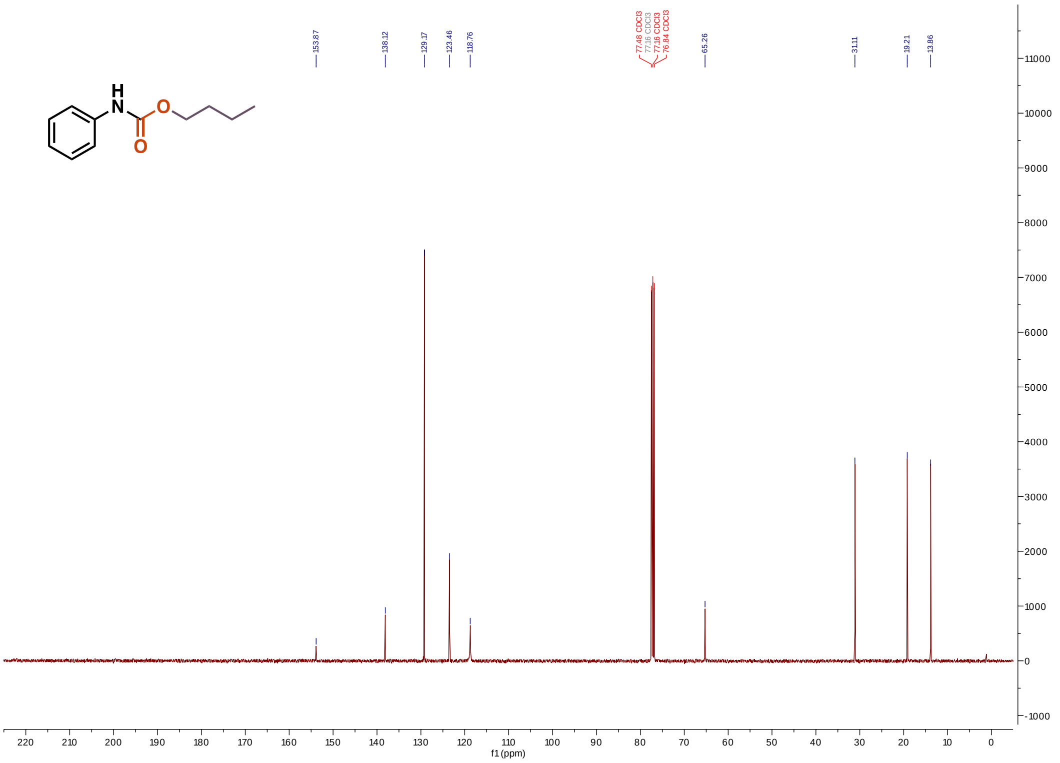

Figure S6. ^{13}C NMR of **3a**

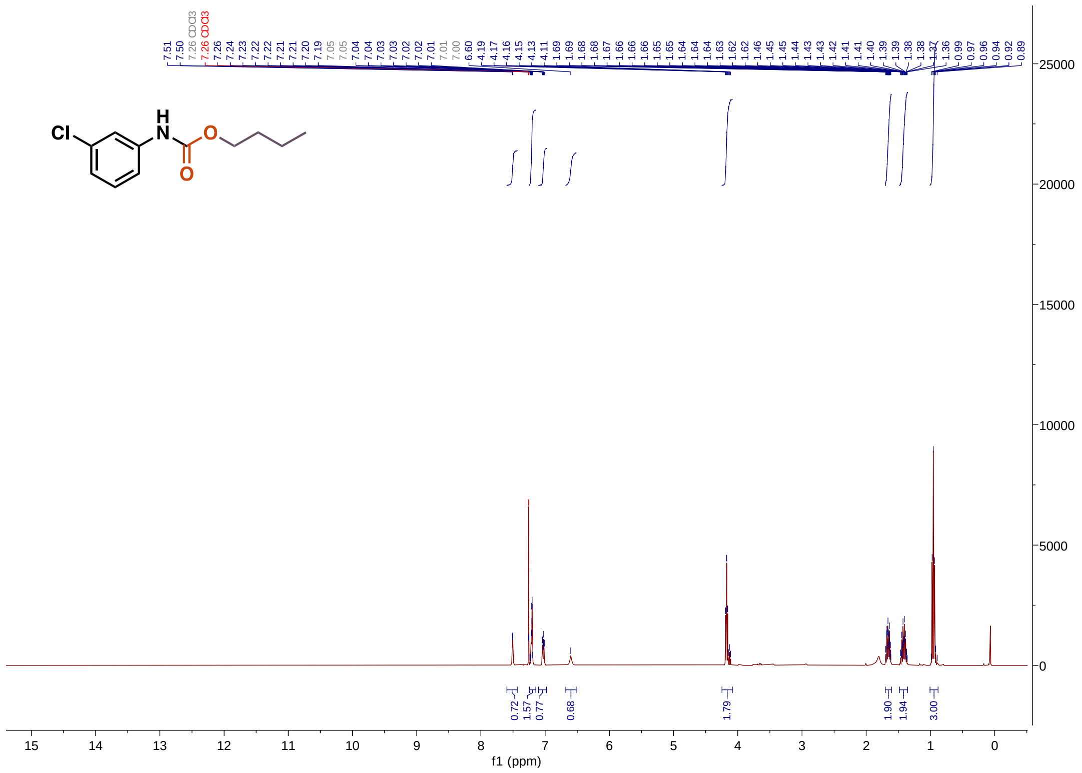

Figure S7. ^{1}H NMR of **3b**

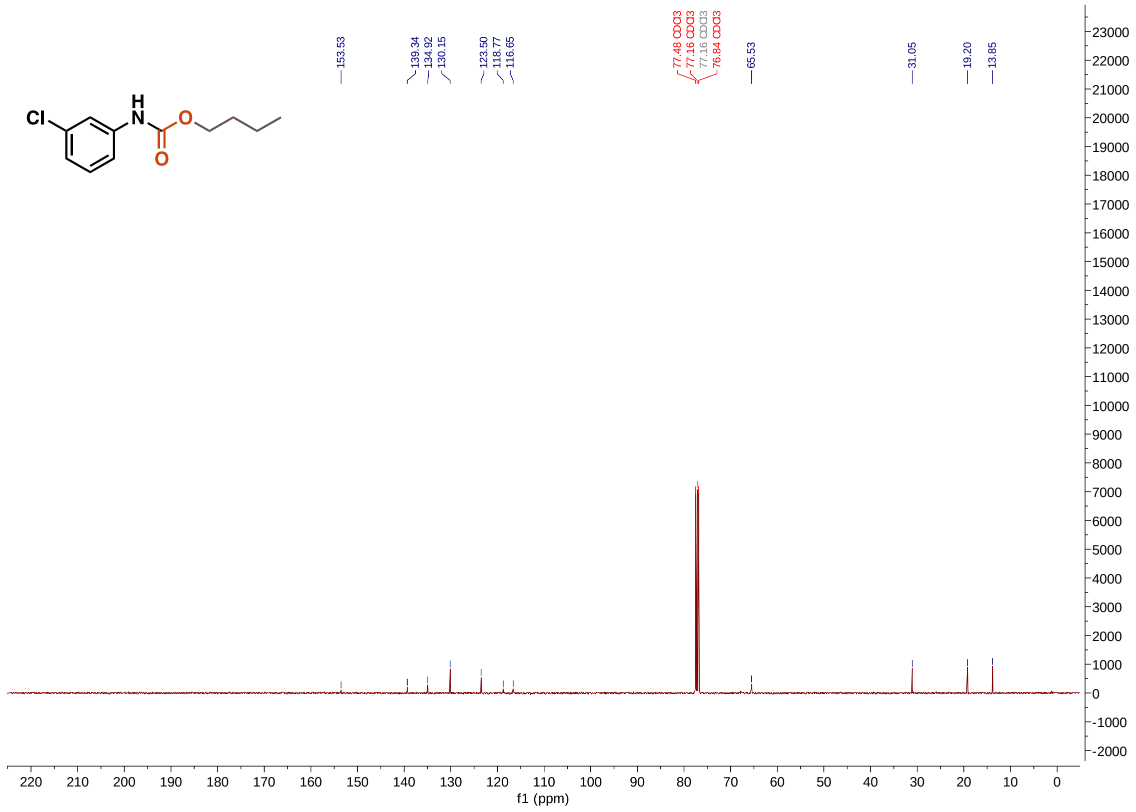

Figure S8. ^{13}C NMR of **3b**

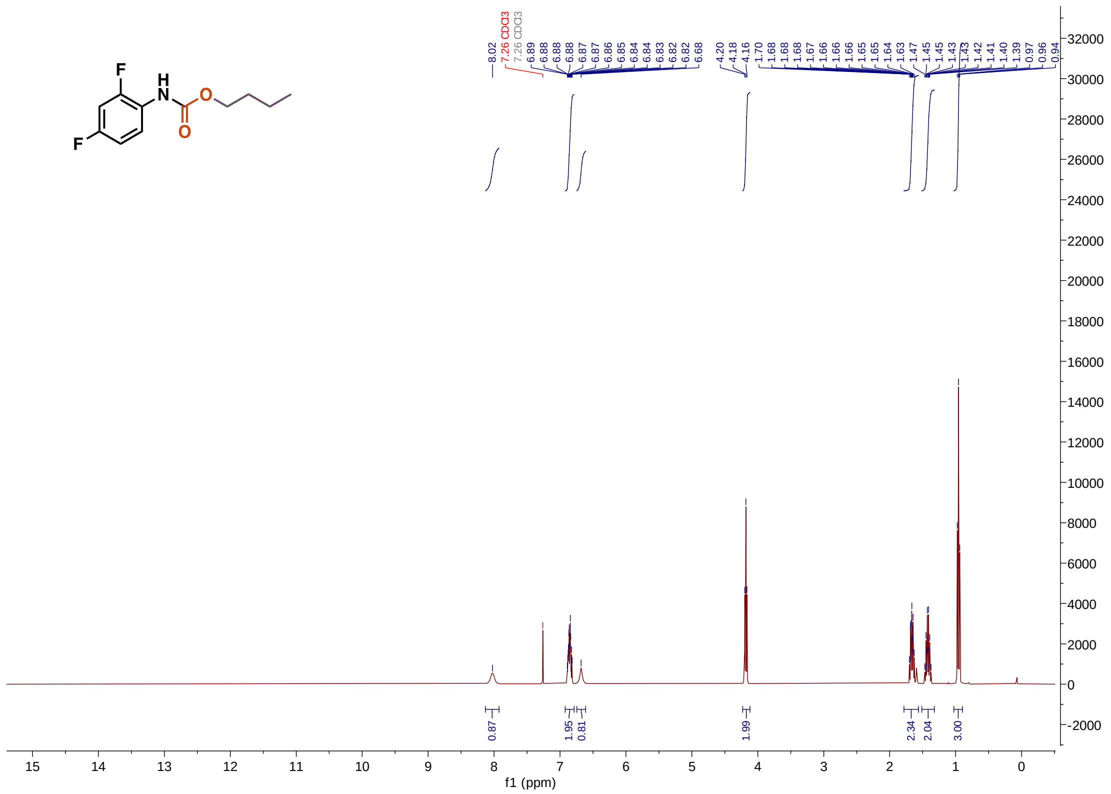

Figure S9. ^{1}H NMR of **3c**

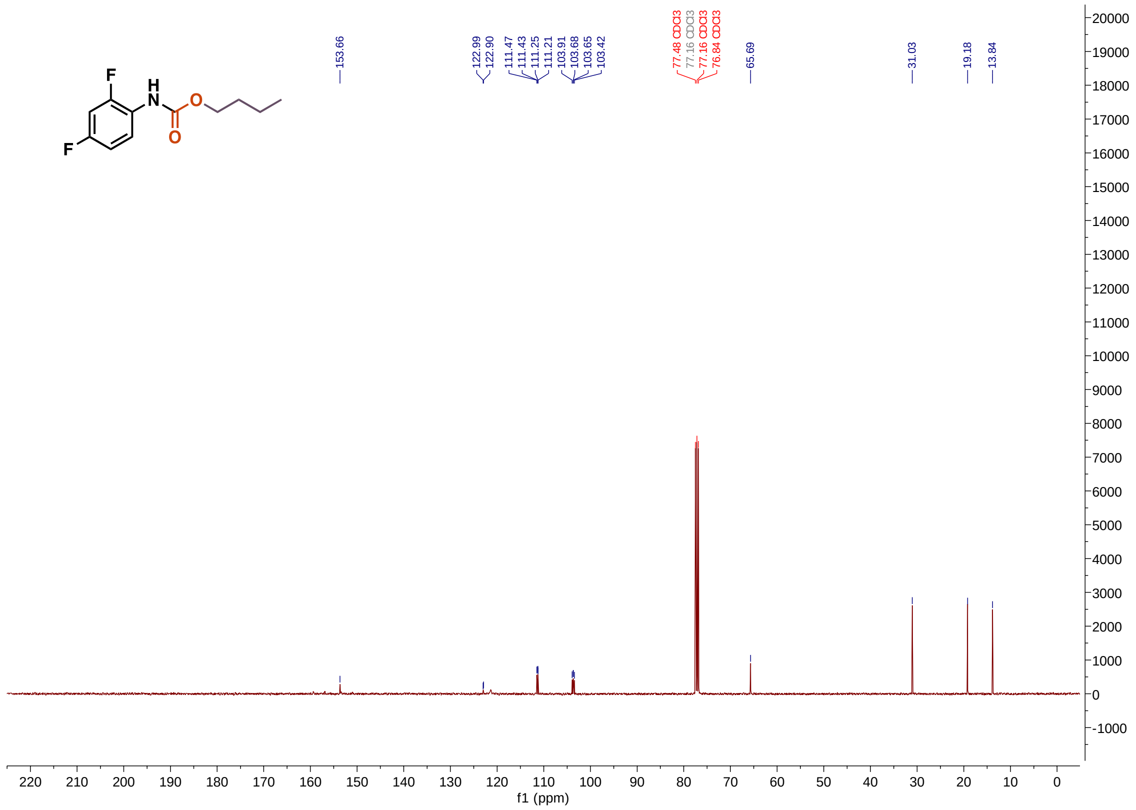

Figure S10. ^{13}C NMR of **3c**

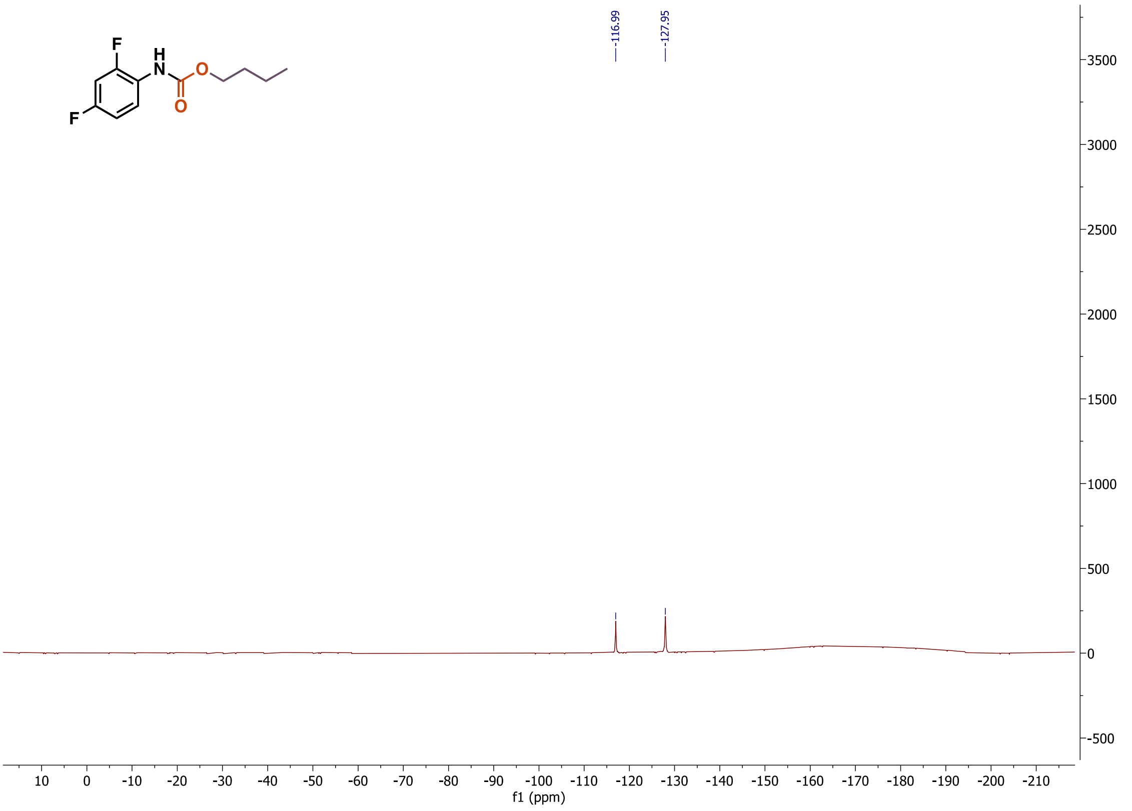

Figure S11. ^{19}F NMR of **3c**

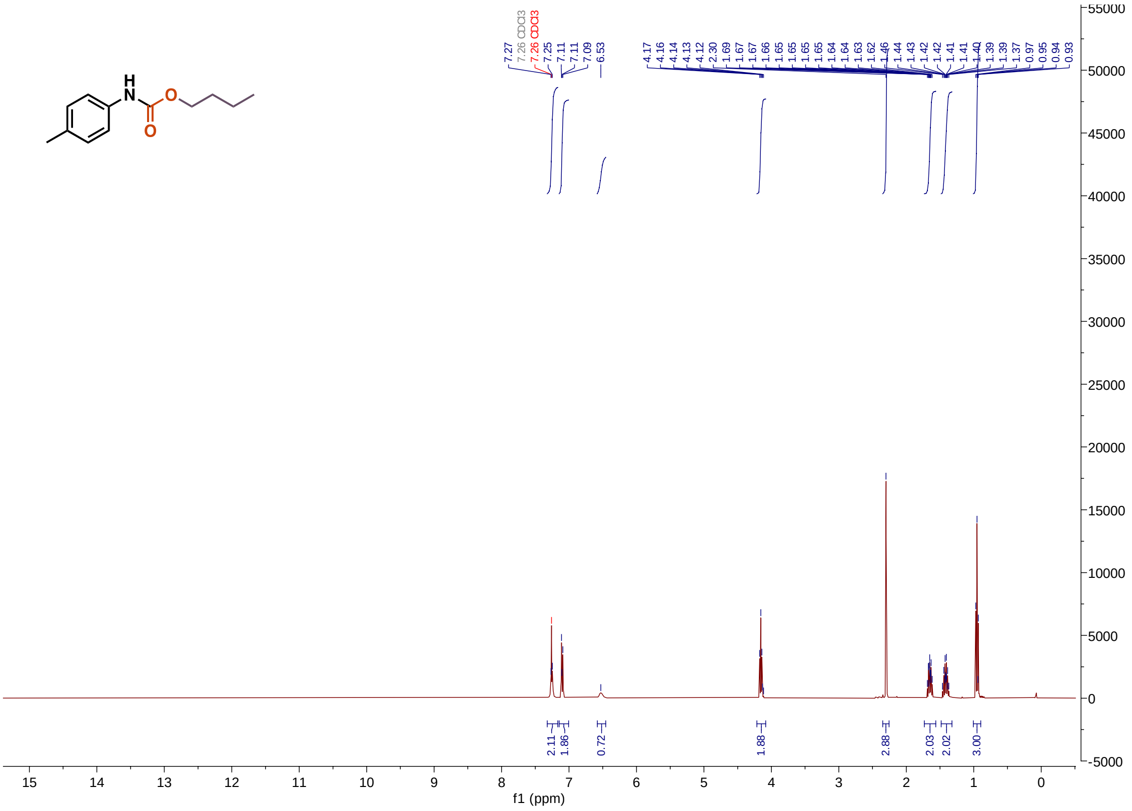

Figure S12. ^{1}H NMR of **3d**

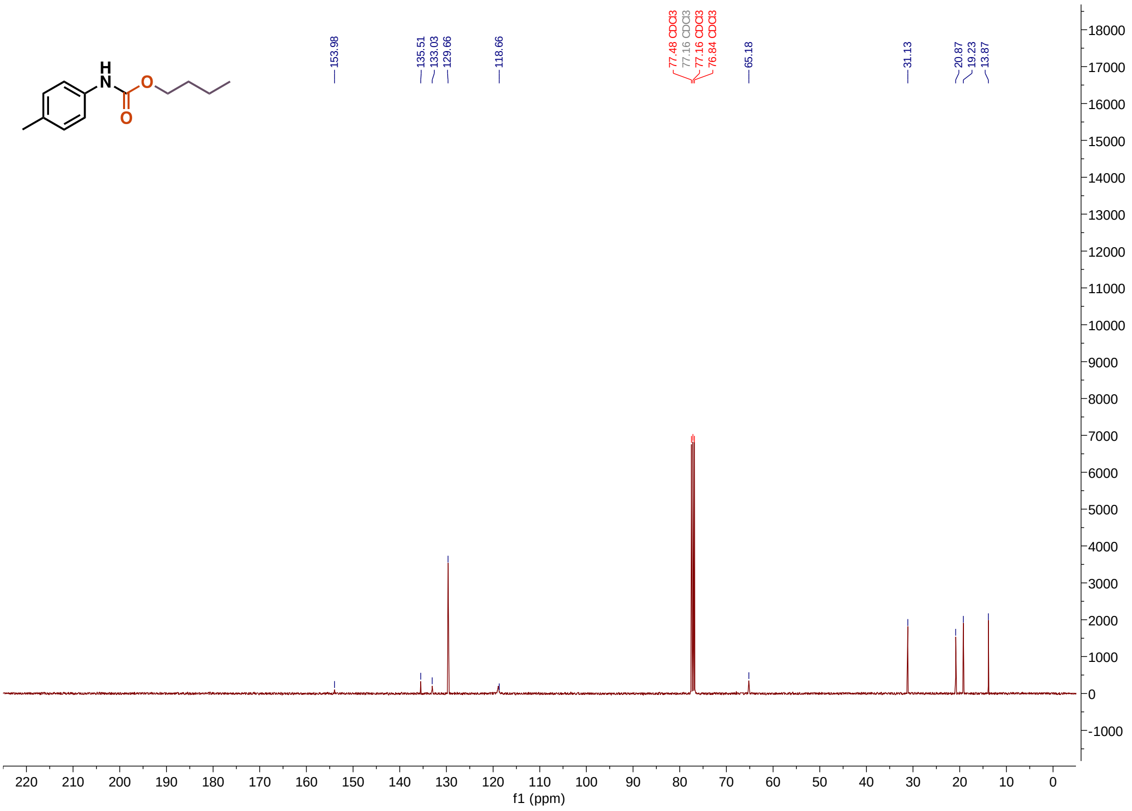

Figure S13. ^{13}C NMR of **3d**

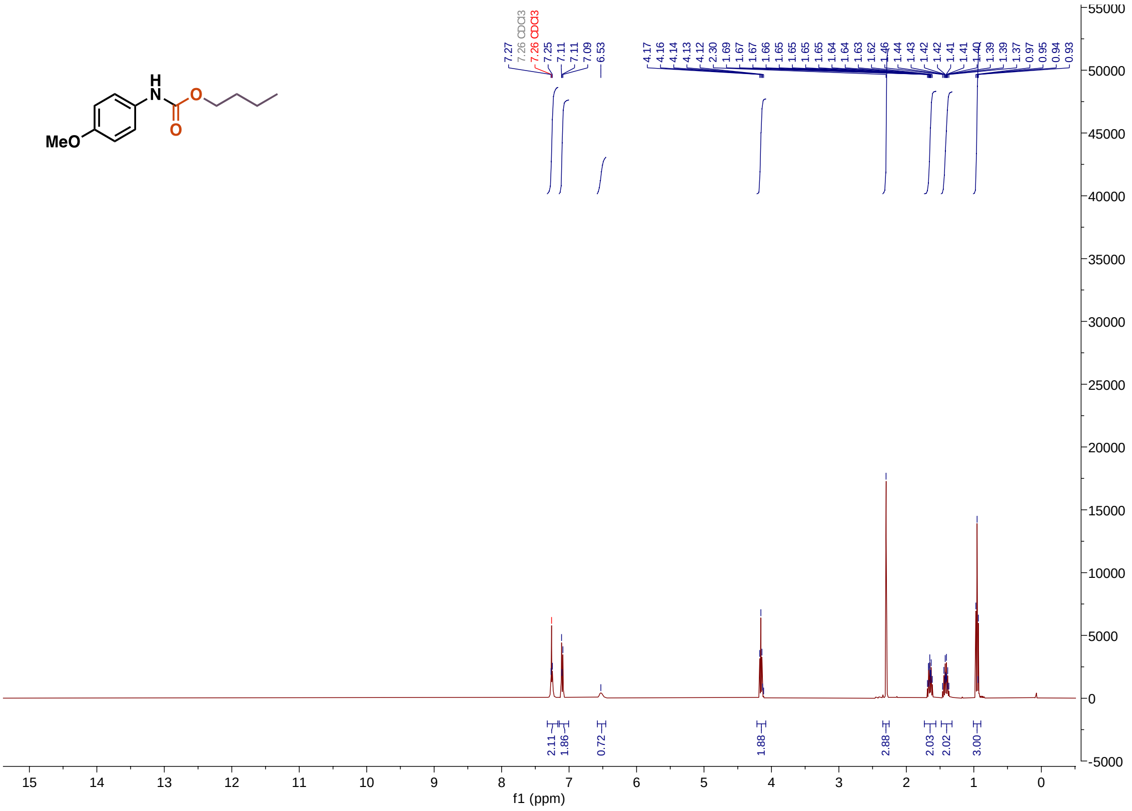

Figure S14. ^{1}H NMR of **3e**

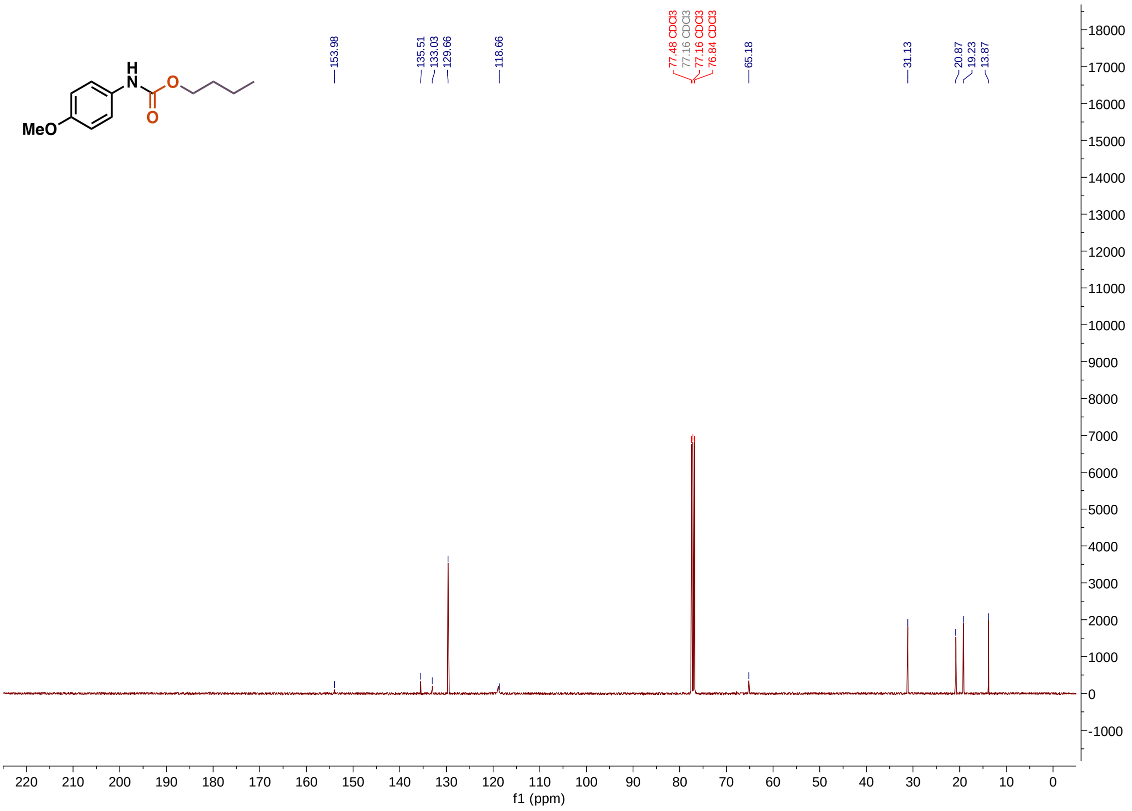

Figure S15. ^{13}C NMR of **3e**

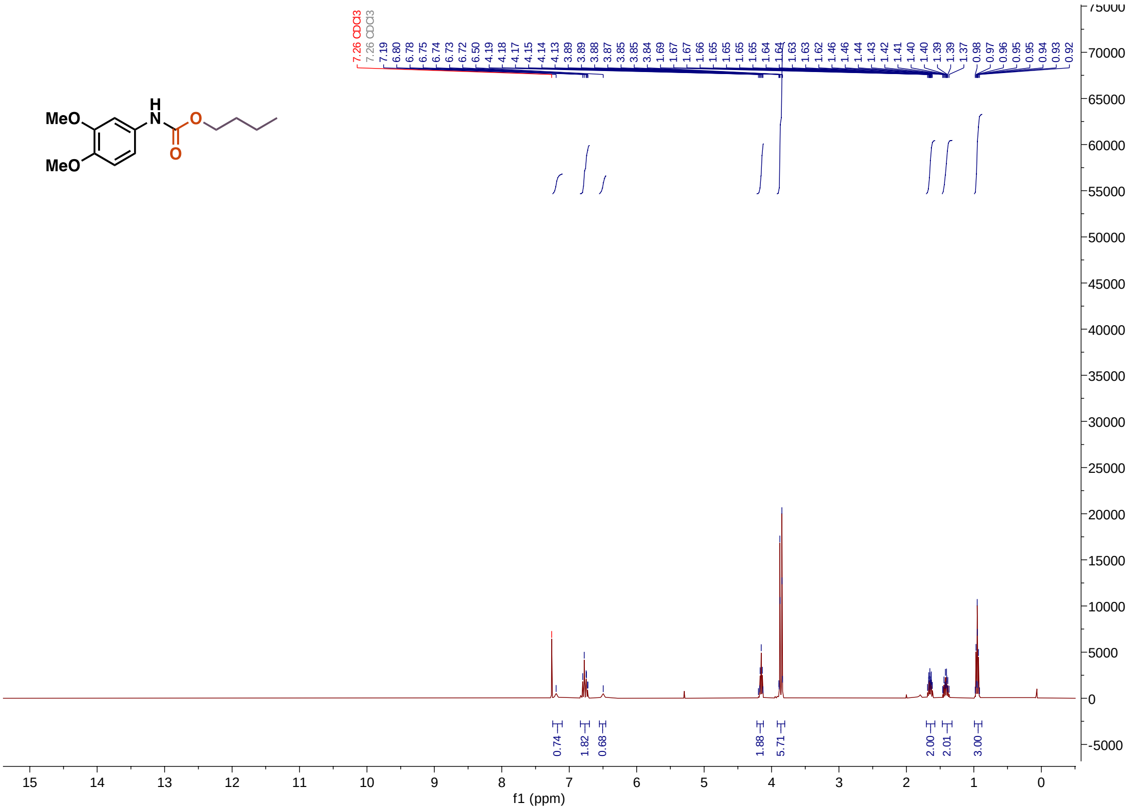

Figure S16. ^{1}H NMR of **3f**

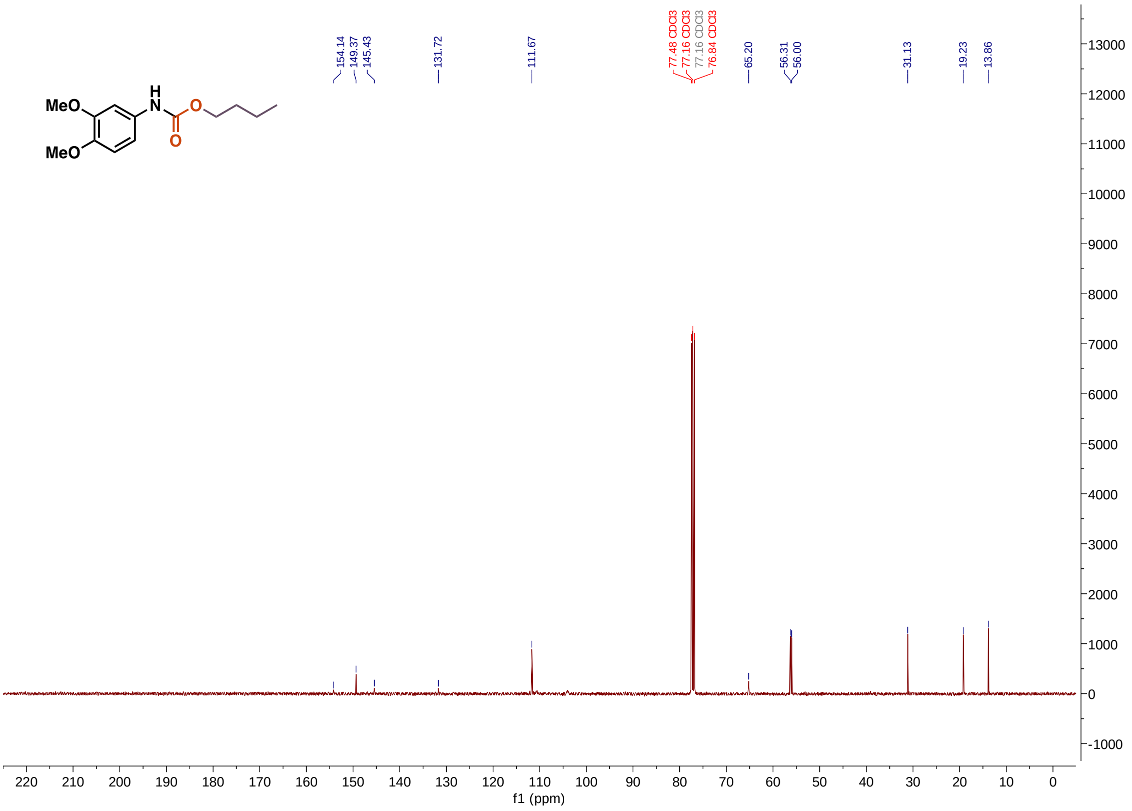

Figure S17. ^{13}C NMR of **3f**

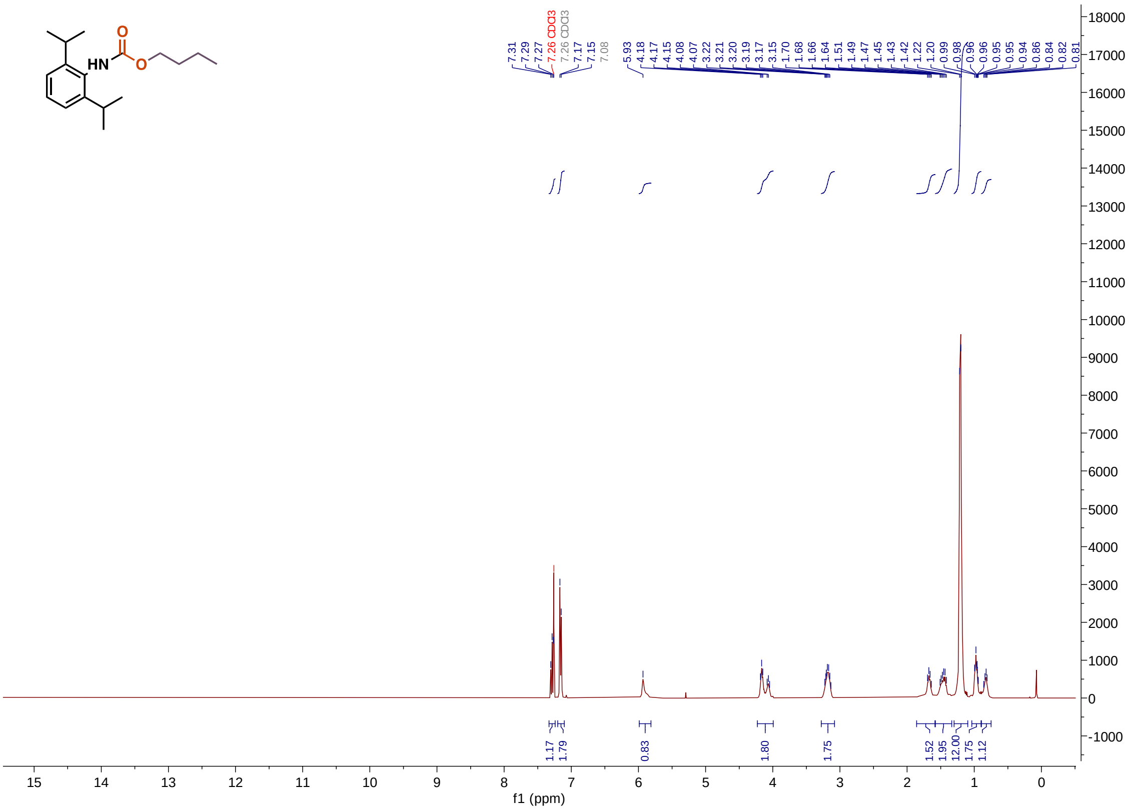

Figure S18. ^{1}H NMR of **3g**

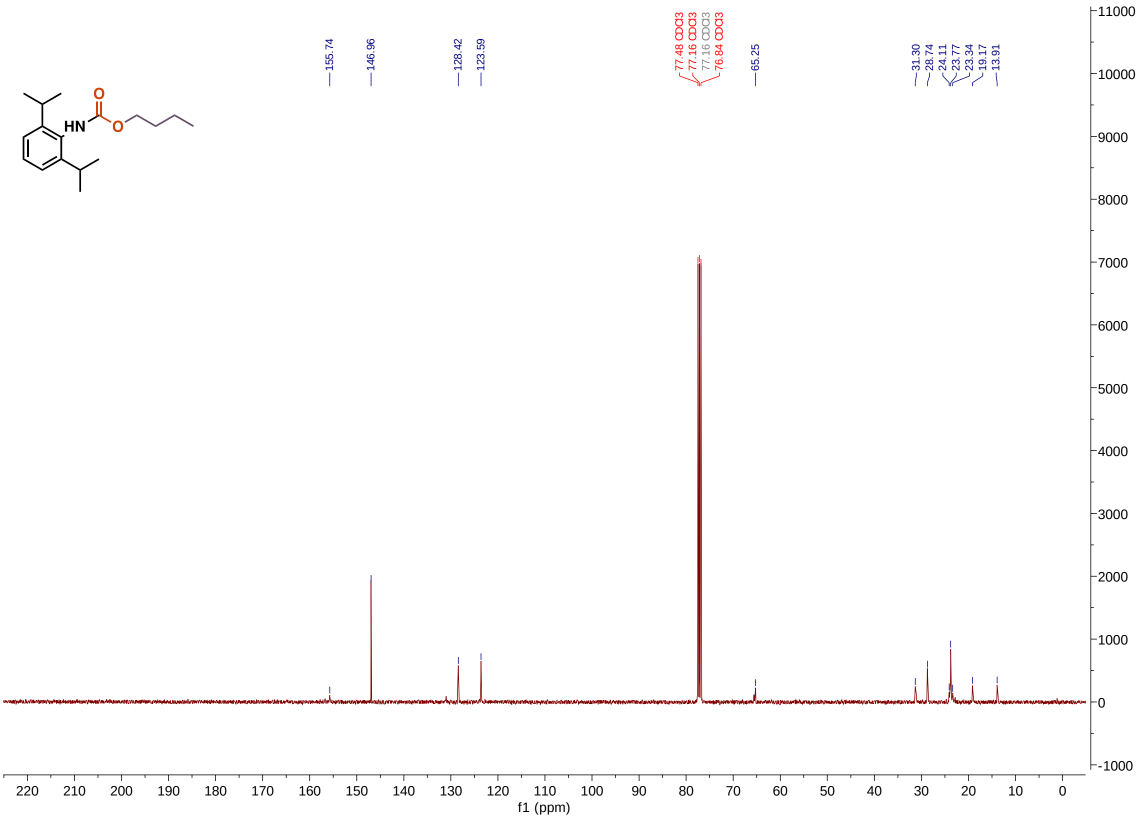

Figure S19. ^{13}C NMR of **3g**

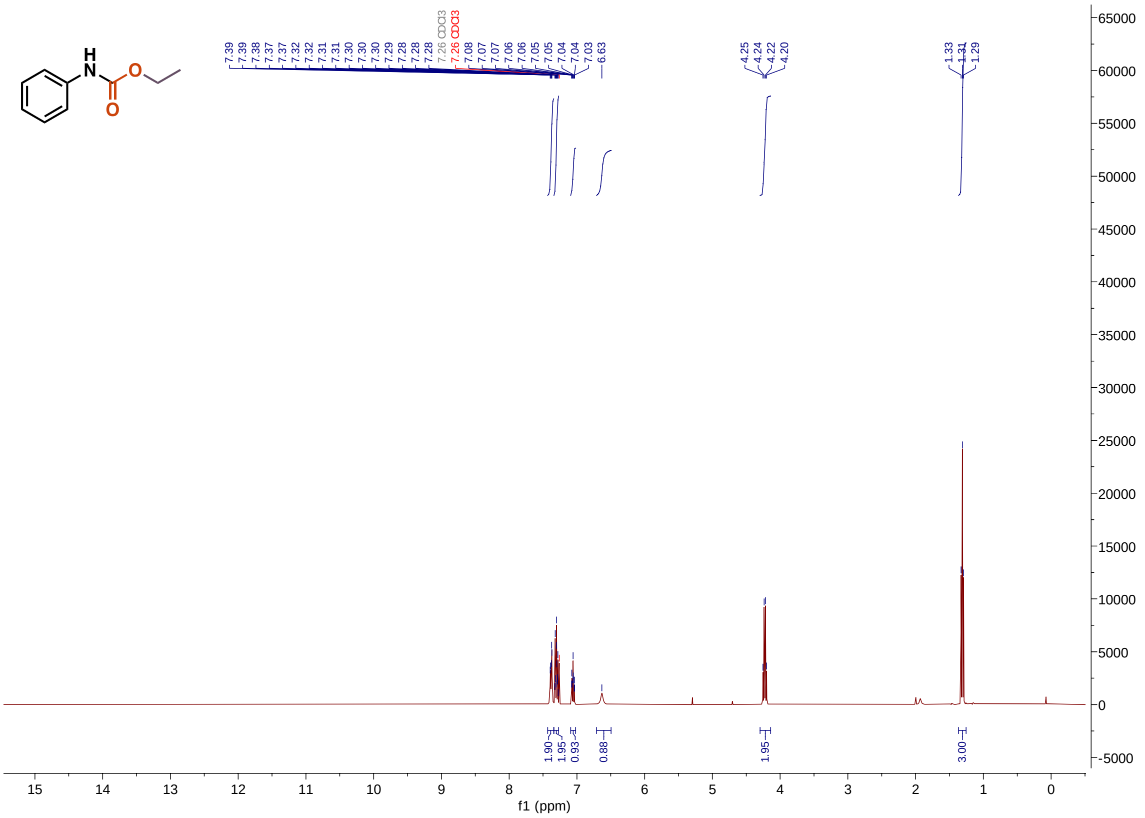

Figure S20. ^{1}H NMR of **3h**

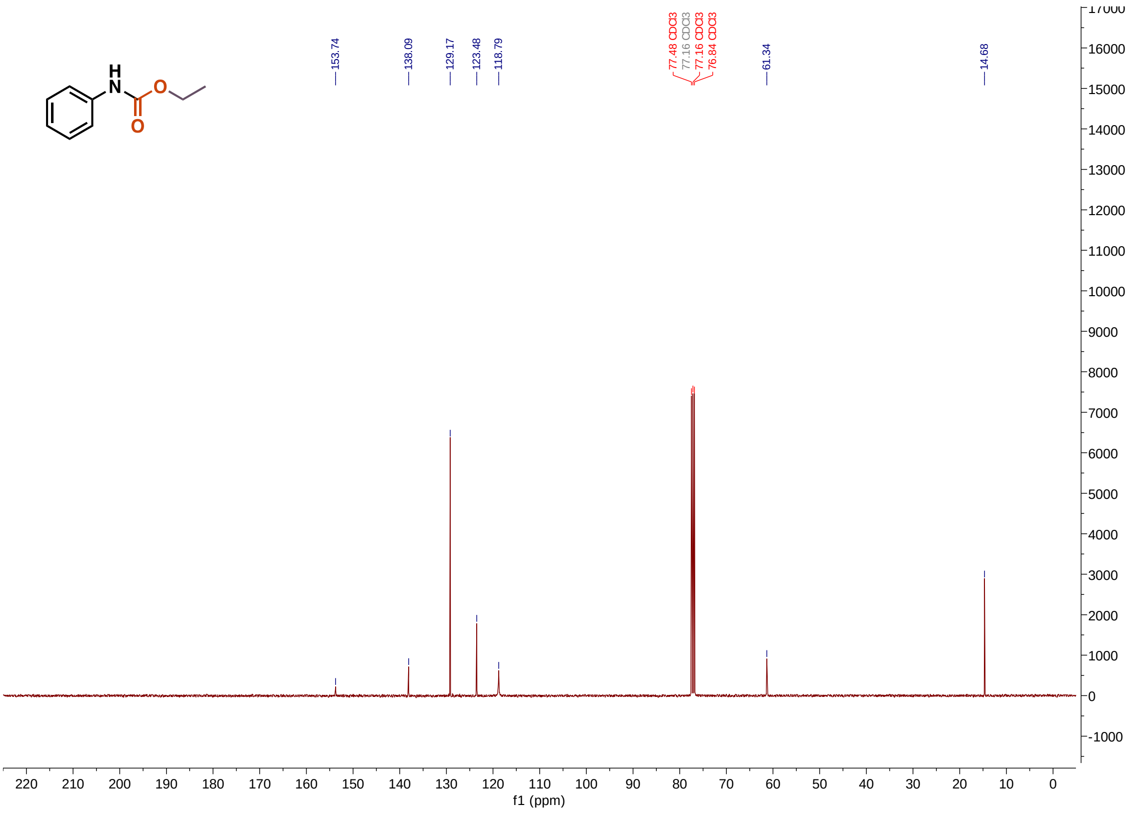

Figure S21. ^{13}C NMR of **3h**

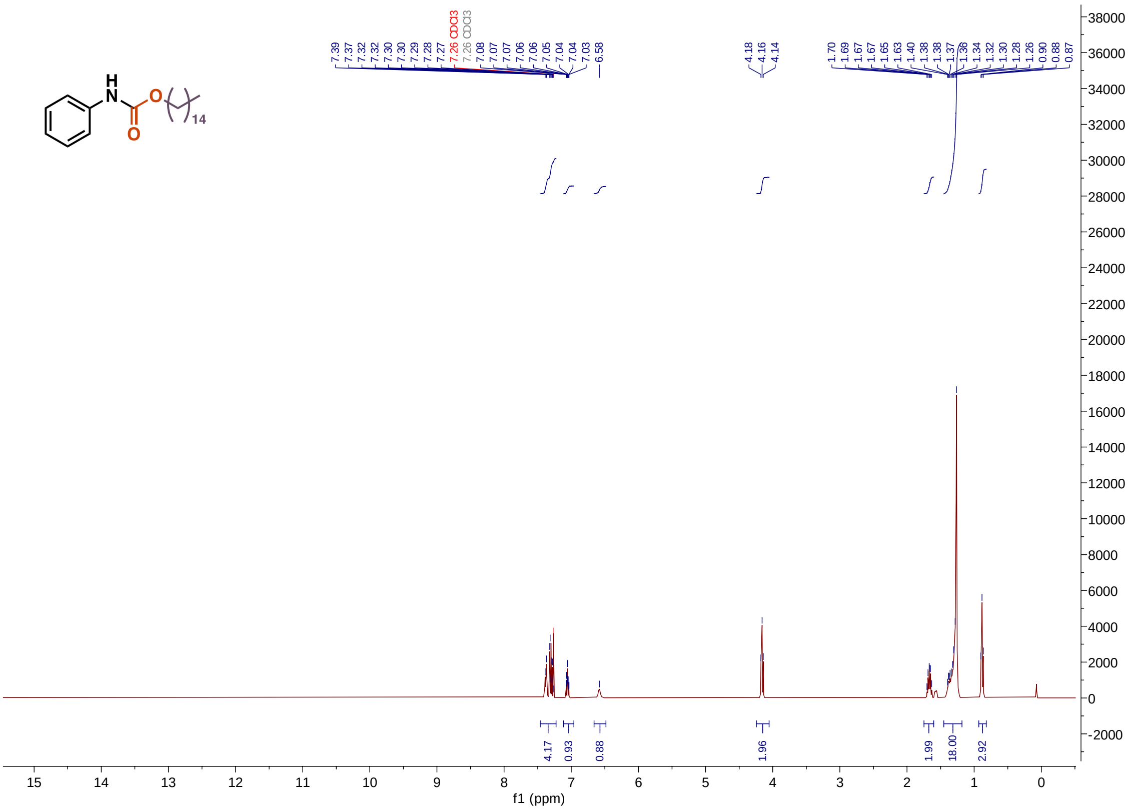

Figure S22. ^{1}H NMR of **3i**

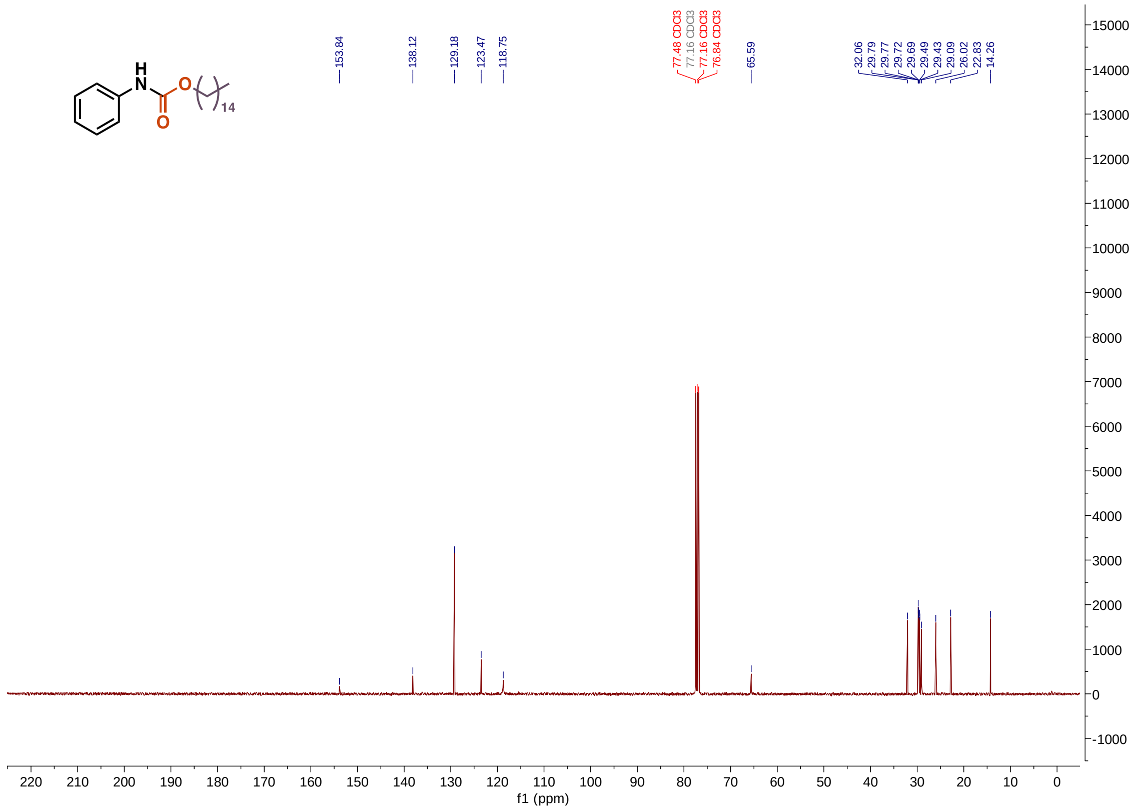

Figure S23. ^{13}C NMR of **3i**

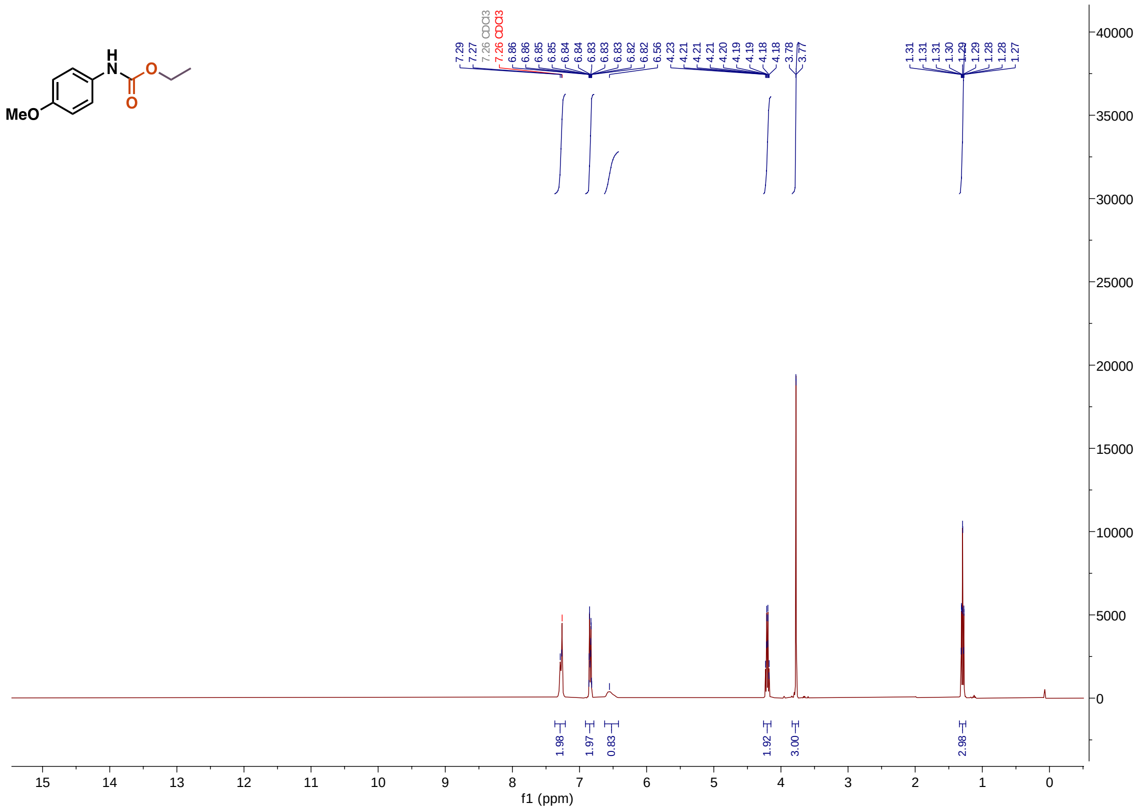

Figure S24. ^{1}H NMR of **3j**

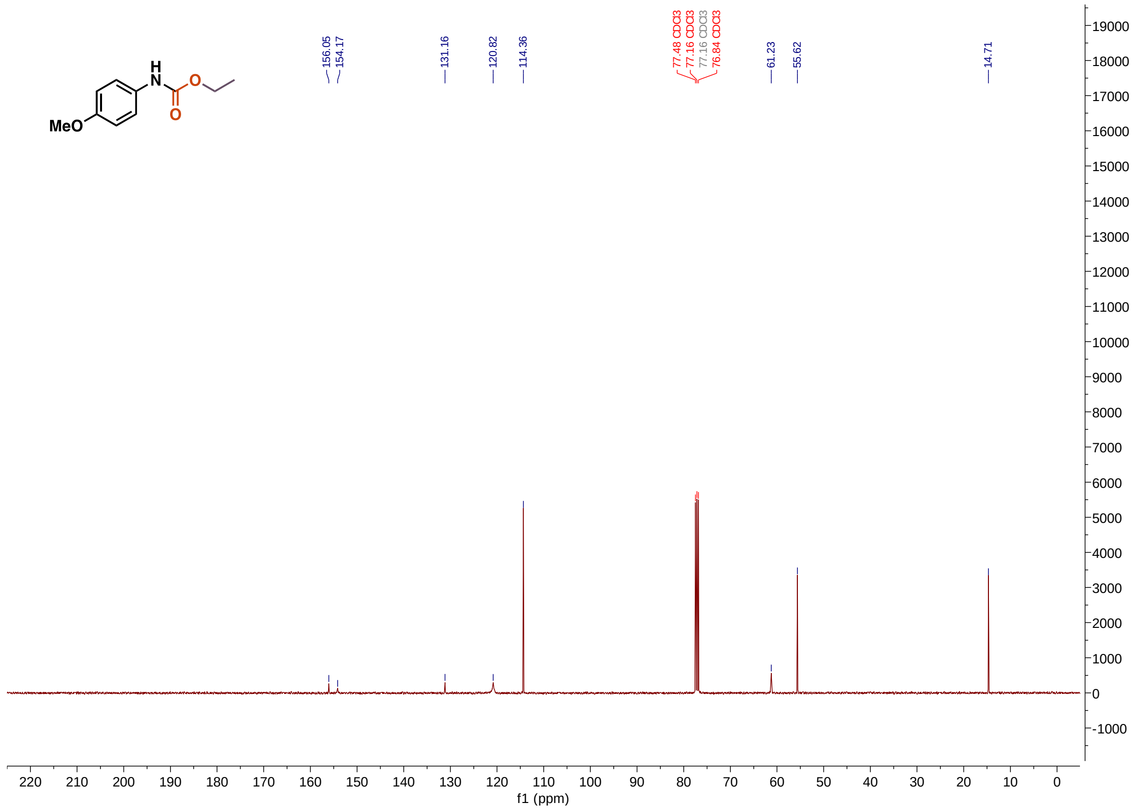

Figure S25. ^{13}C NMR of **3j**

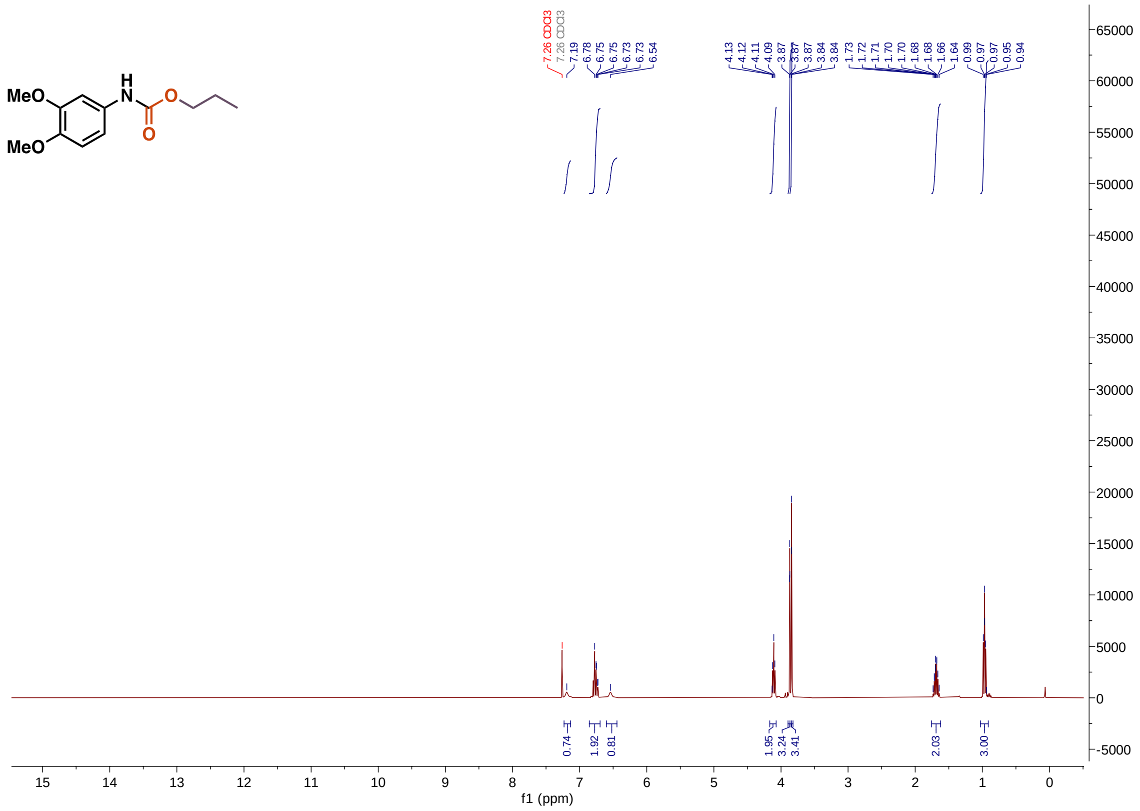

Figure S26. ^{1}H NMR of **3k**

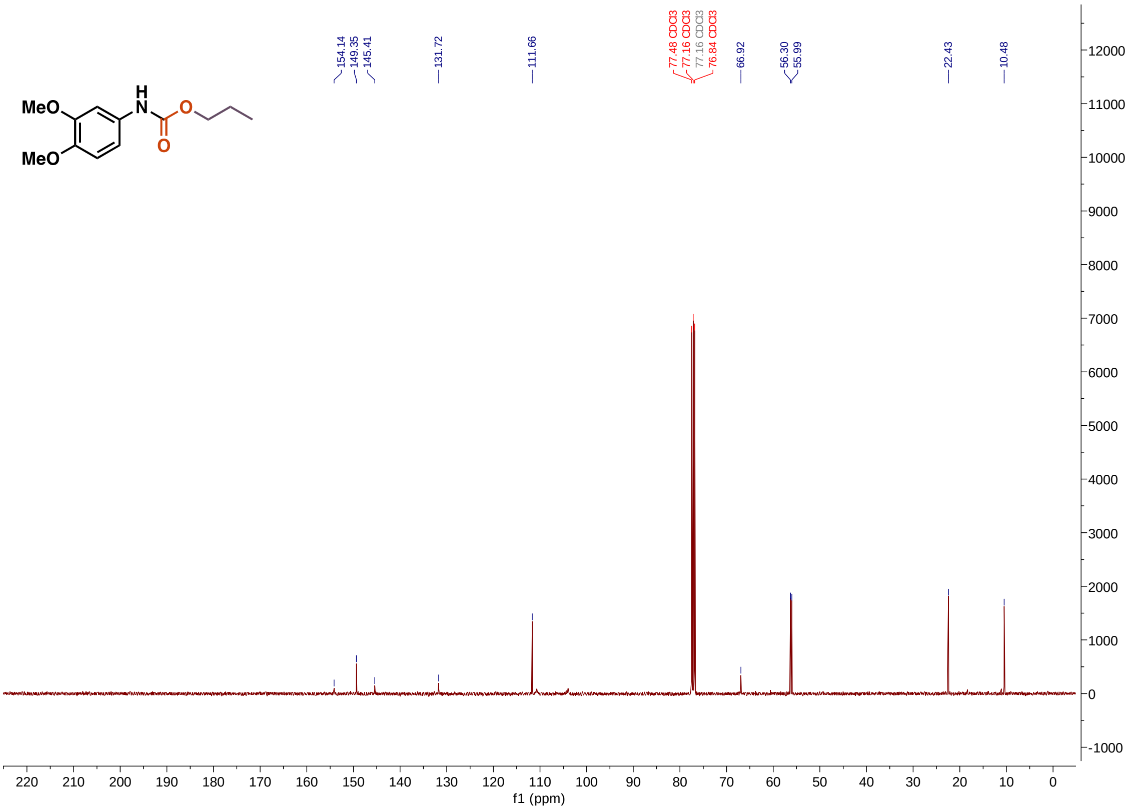

Figure S27. ^{13}C NMR of **3k**

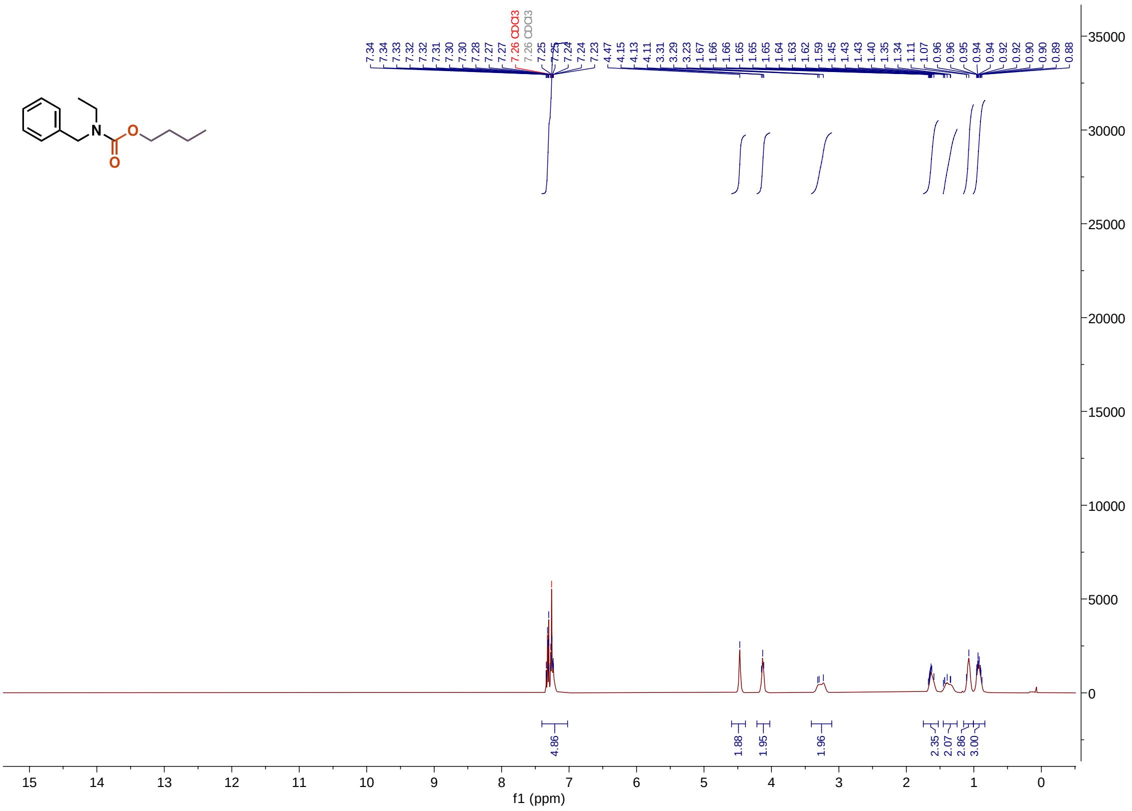

Figure S28. ^{1}H NMR of **3l**

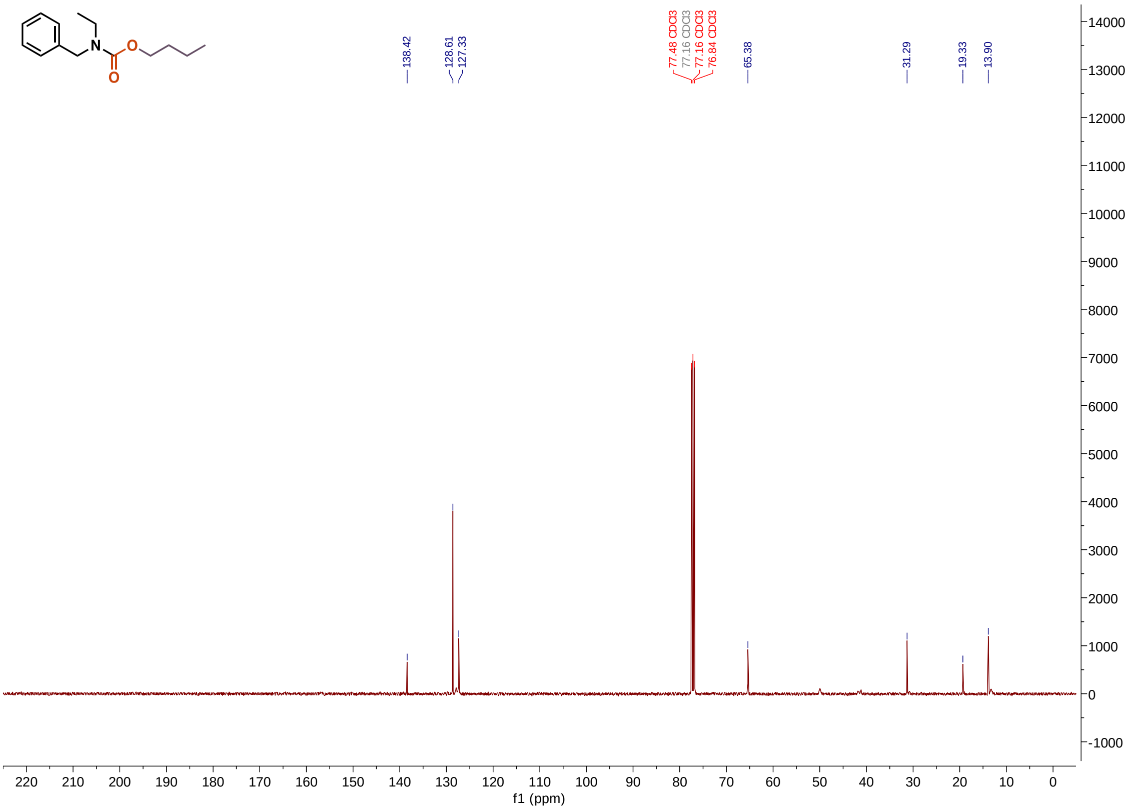

Figure S29. ^{13}C NMR of **3l**

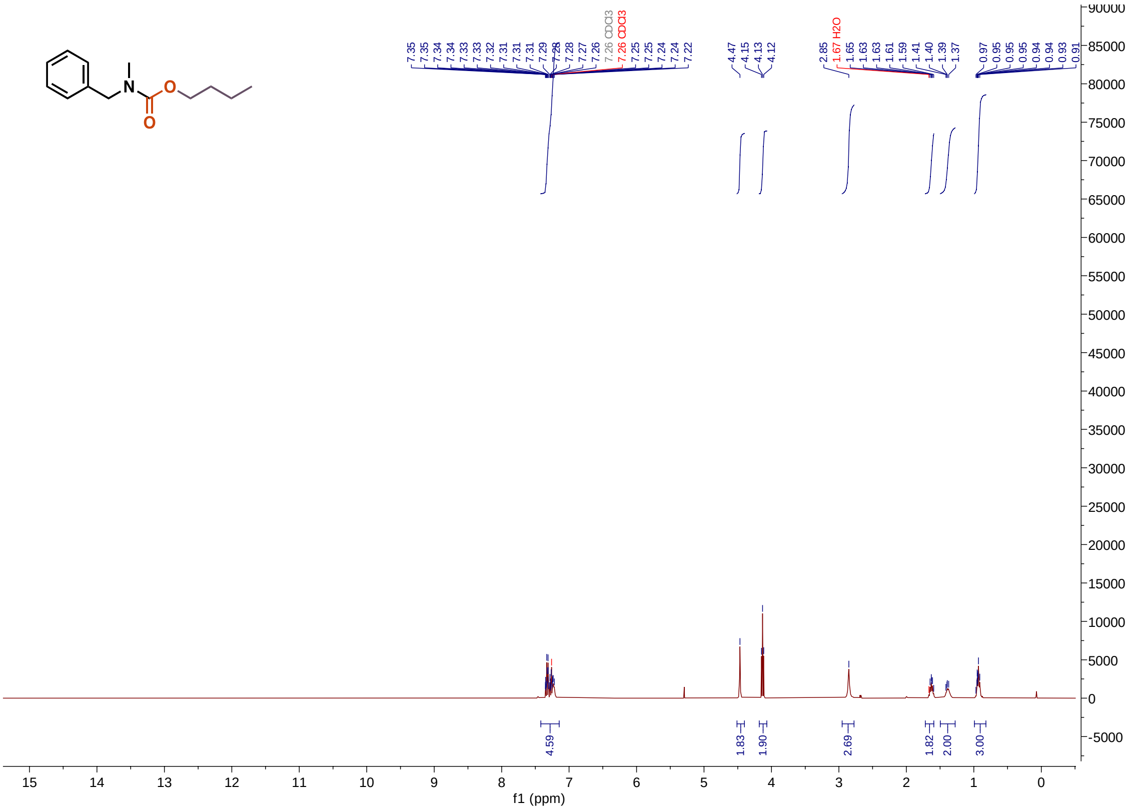

Figure S30. ^{1}H NMR of **3m**

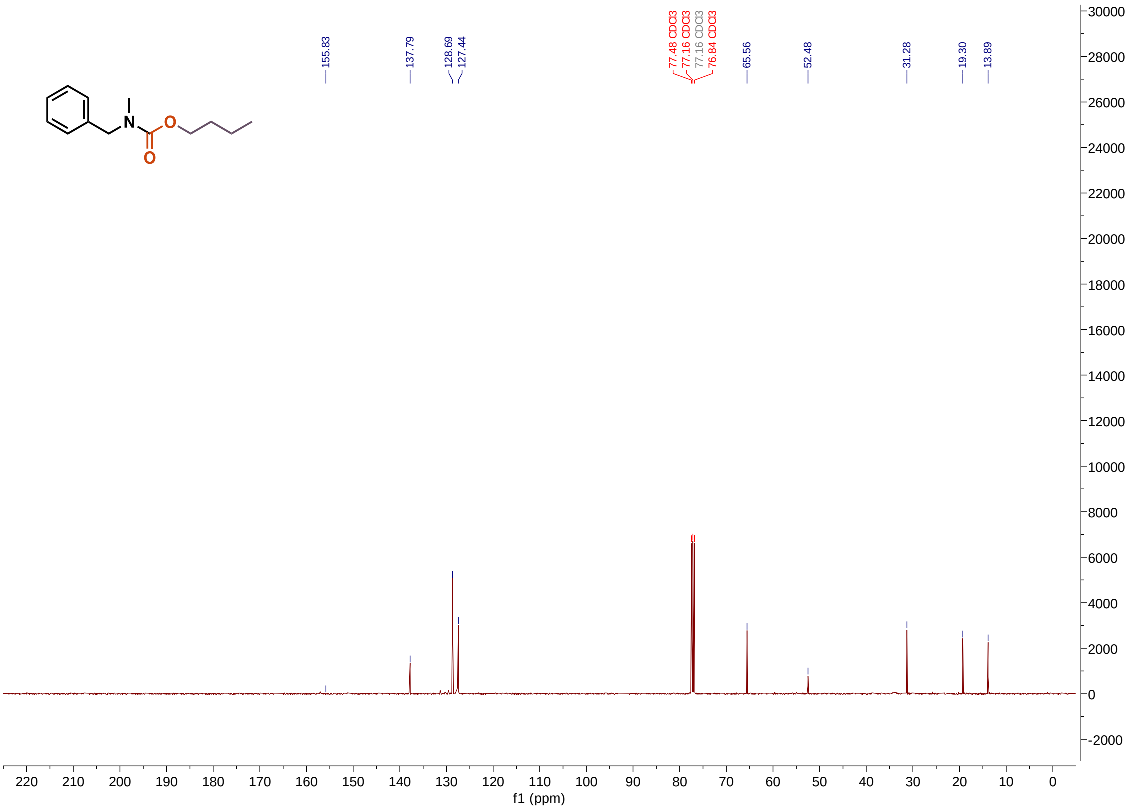

Figure S31. ^{13}C NMR of **3m**

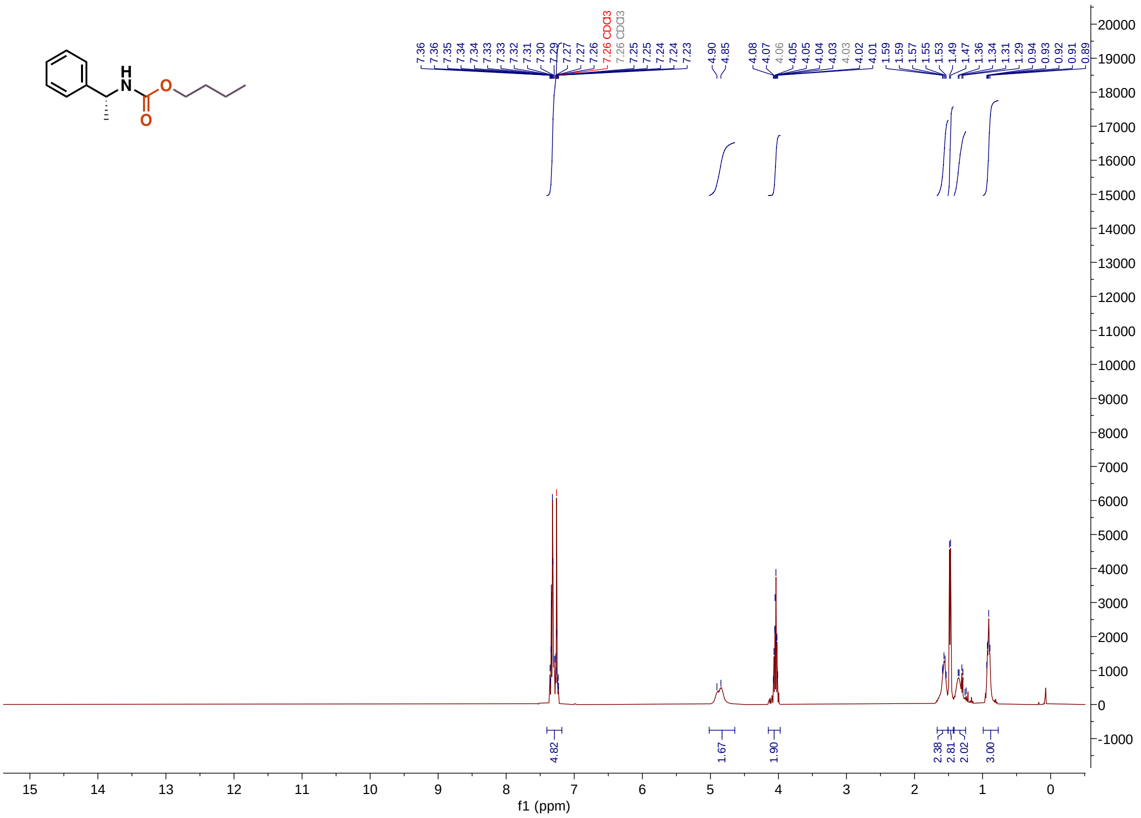

Figure S32. ^{1}H NMR of **3n**

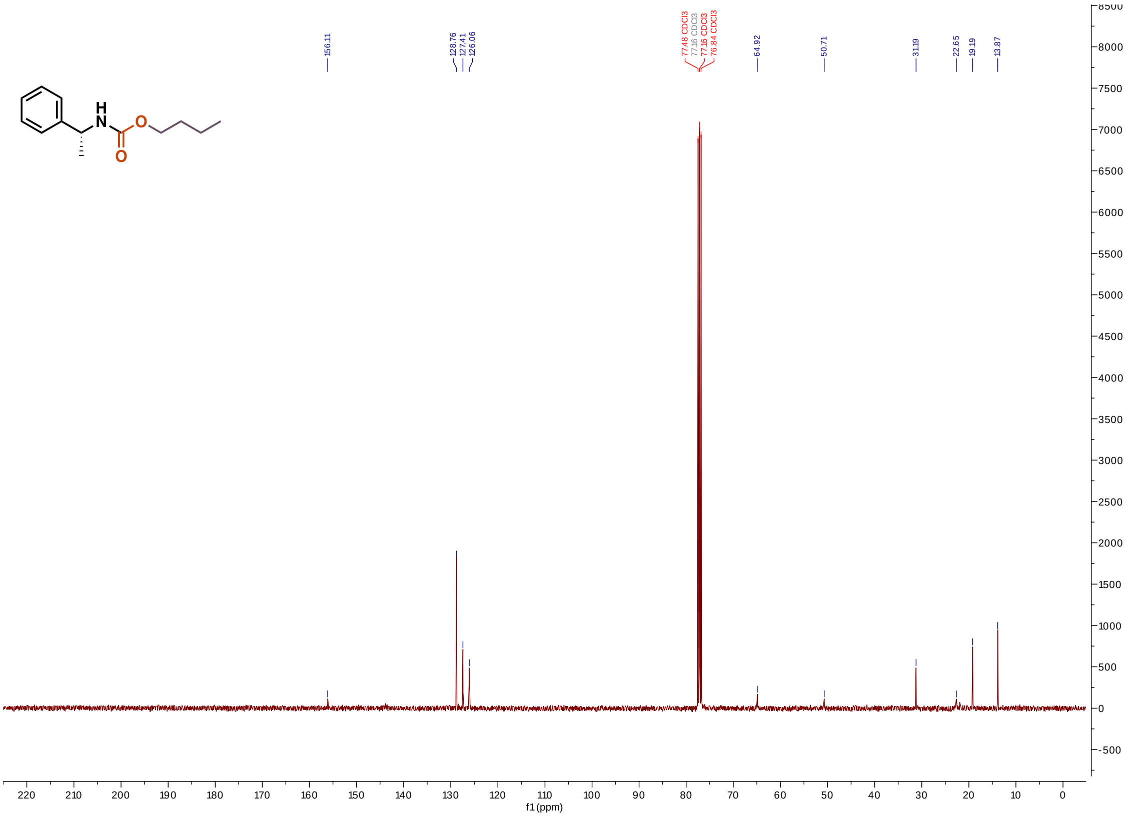

Figure S33. ^{13}C NMR of **3n**

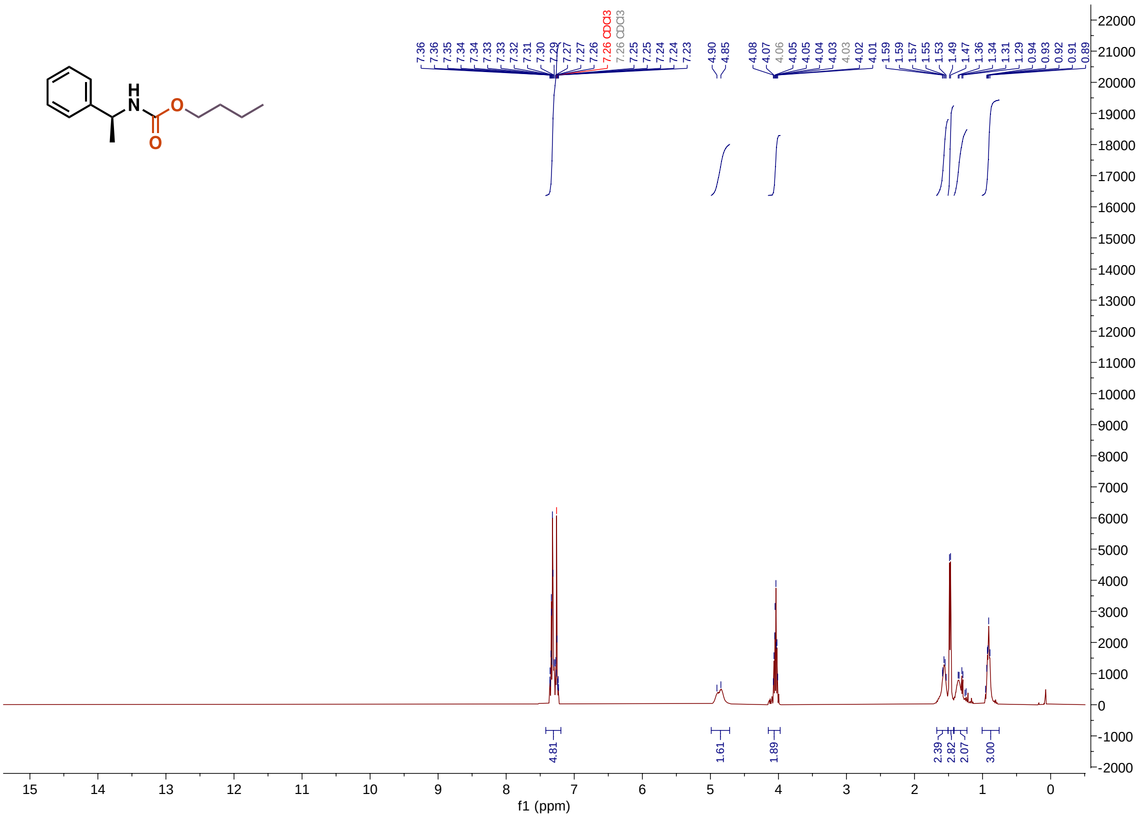

Figure S34. ^{1}H NMR of **3o**

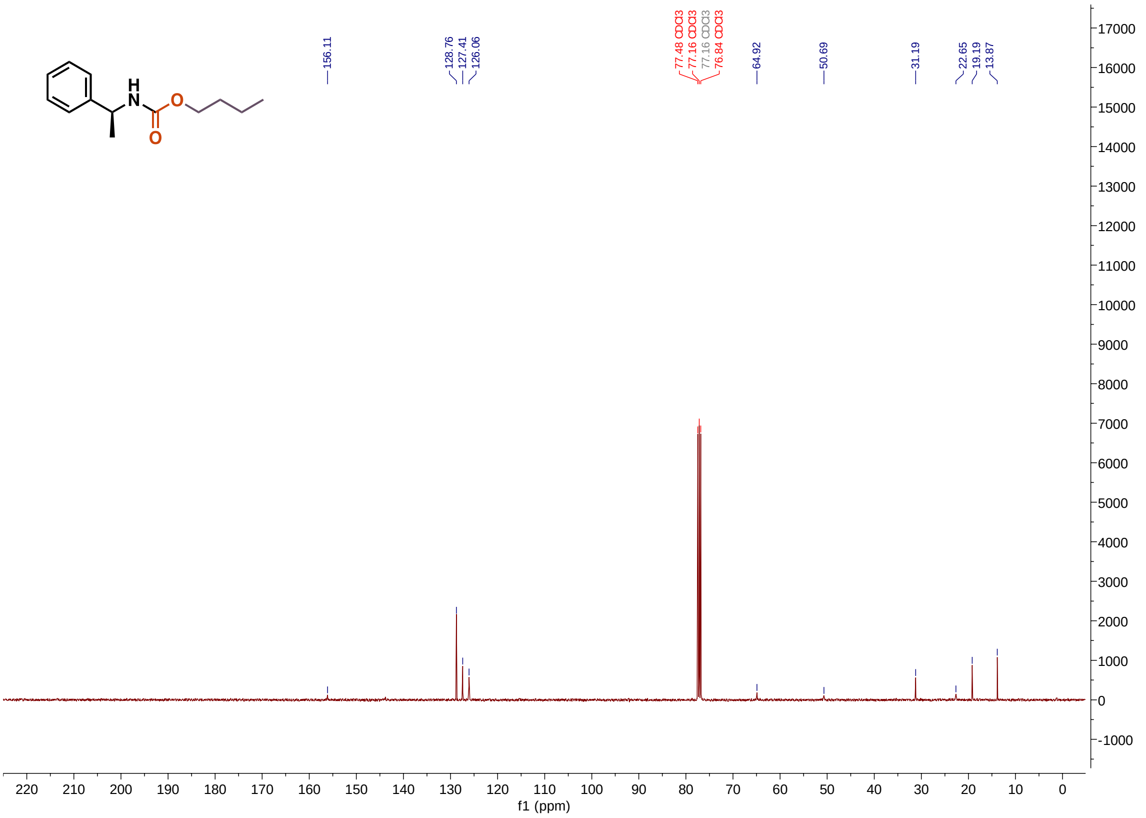

Figure S35. ^{13}C NMR of **3o**

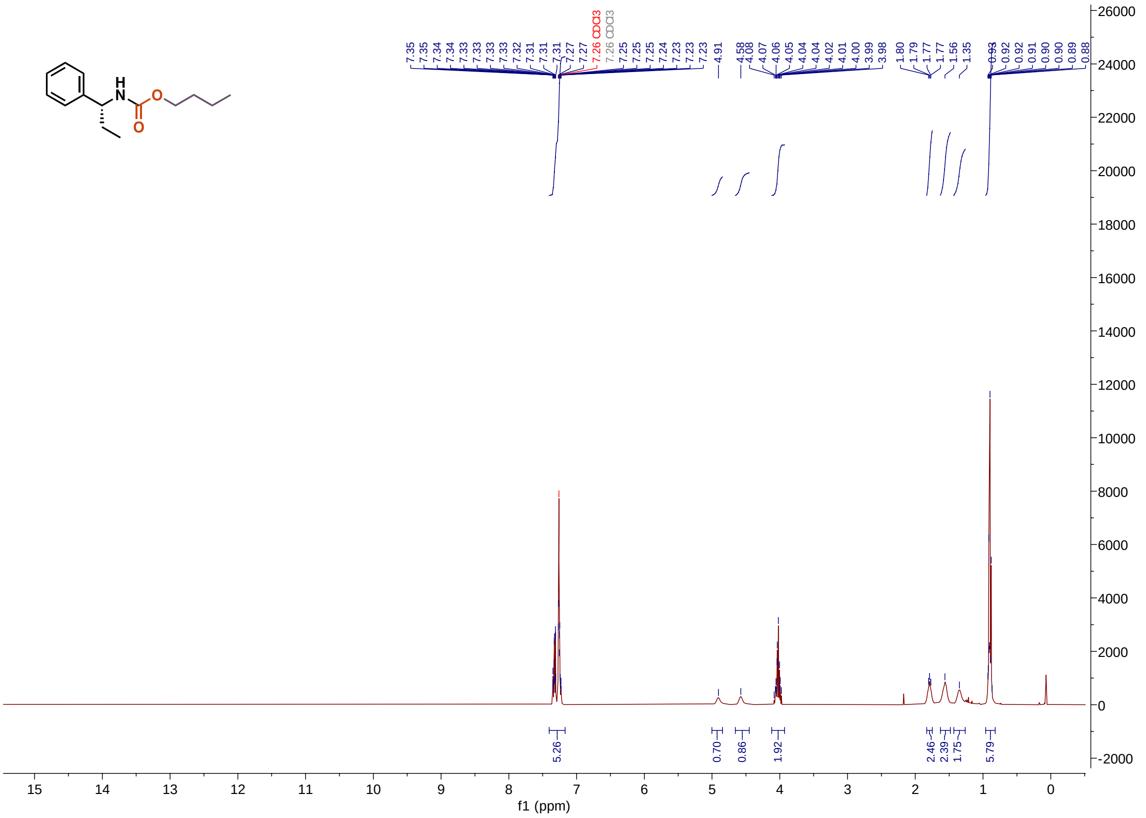

Figure S36. ^{1}H NMR of **3p**

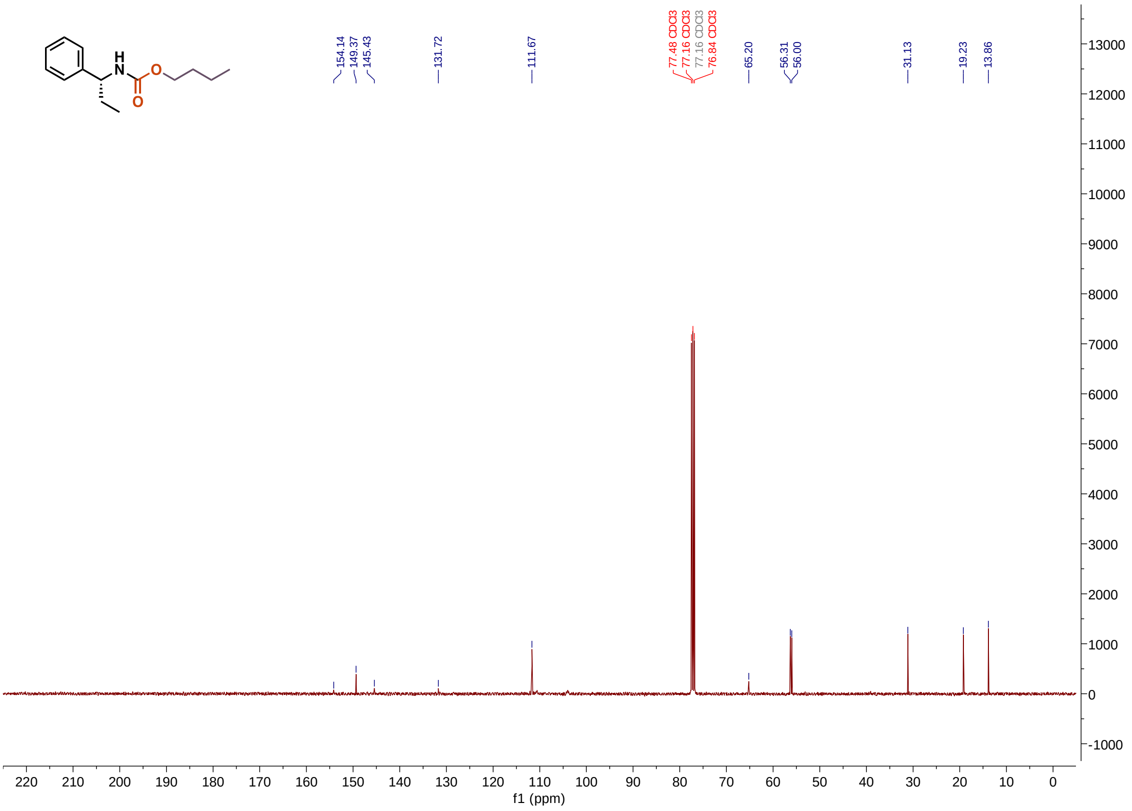

Figure S37. ^{13}C NMR of **3p**

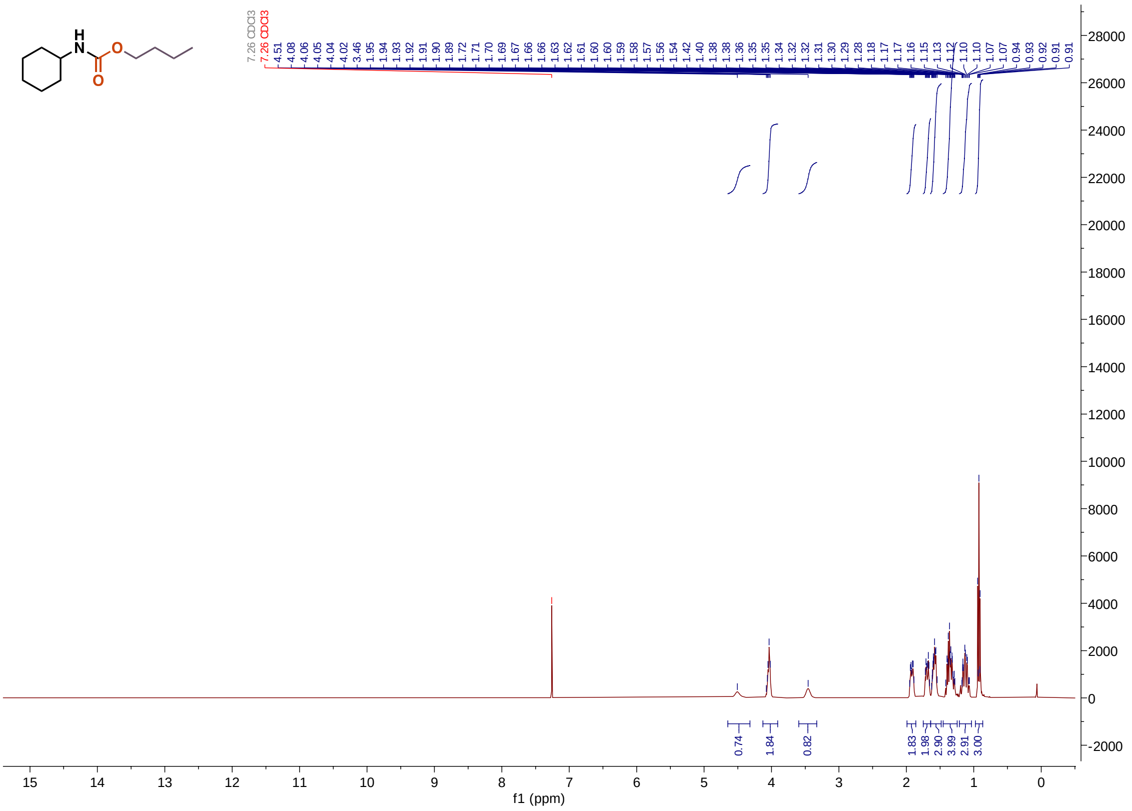

Figure S38. ^{1}H NMR of **3q**

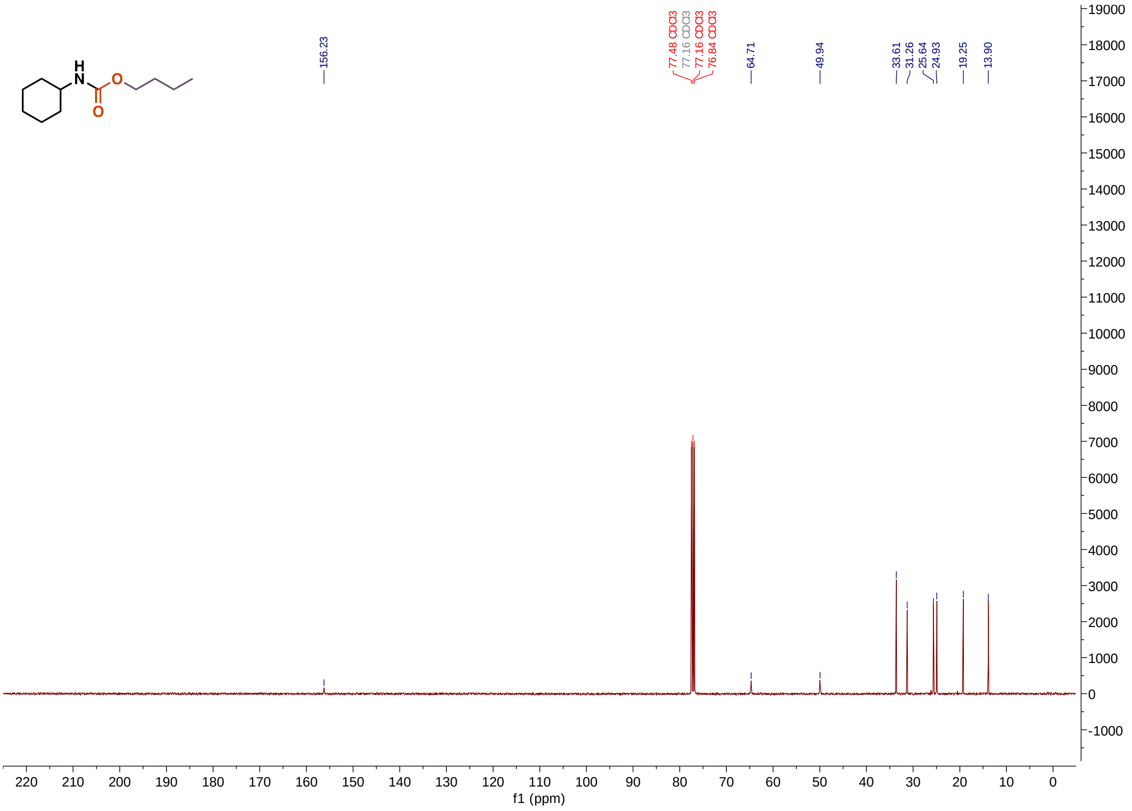

Figure S39. ^{13}C NMR of **3q**

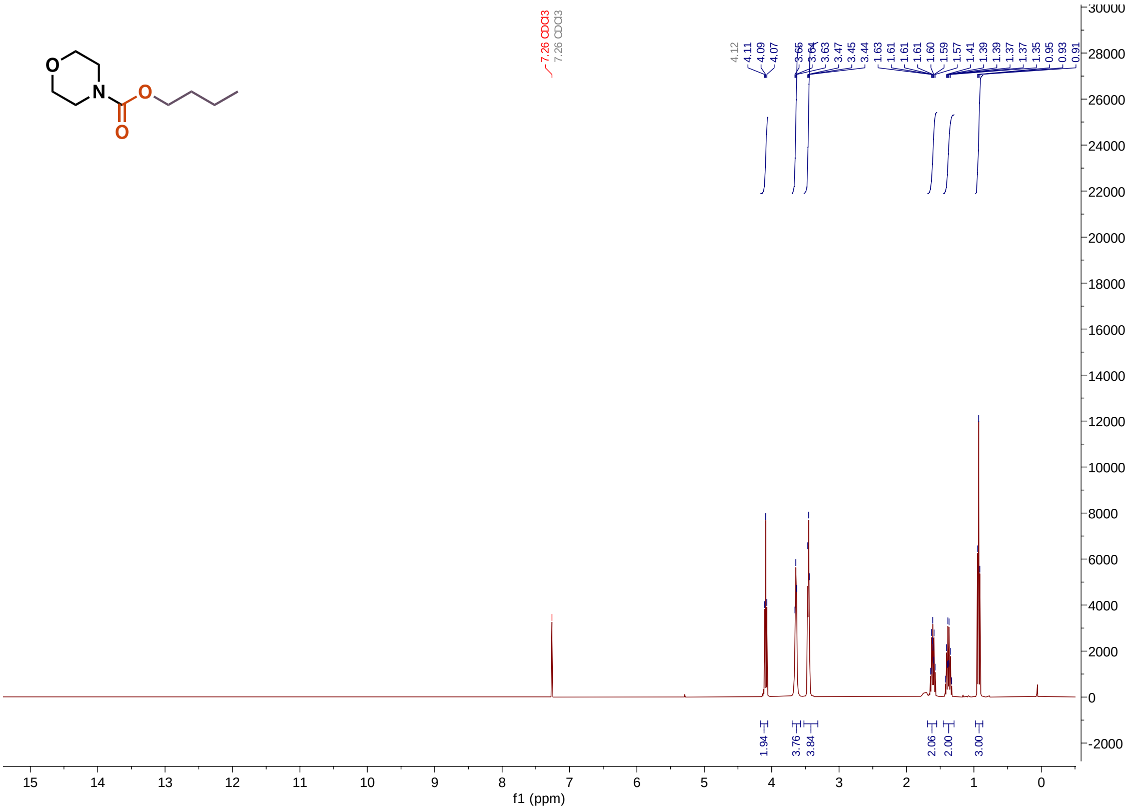

Figure S40. ^{1}H NMR of **3r**

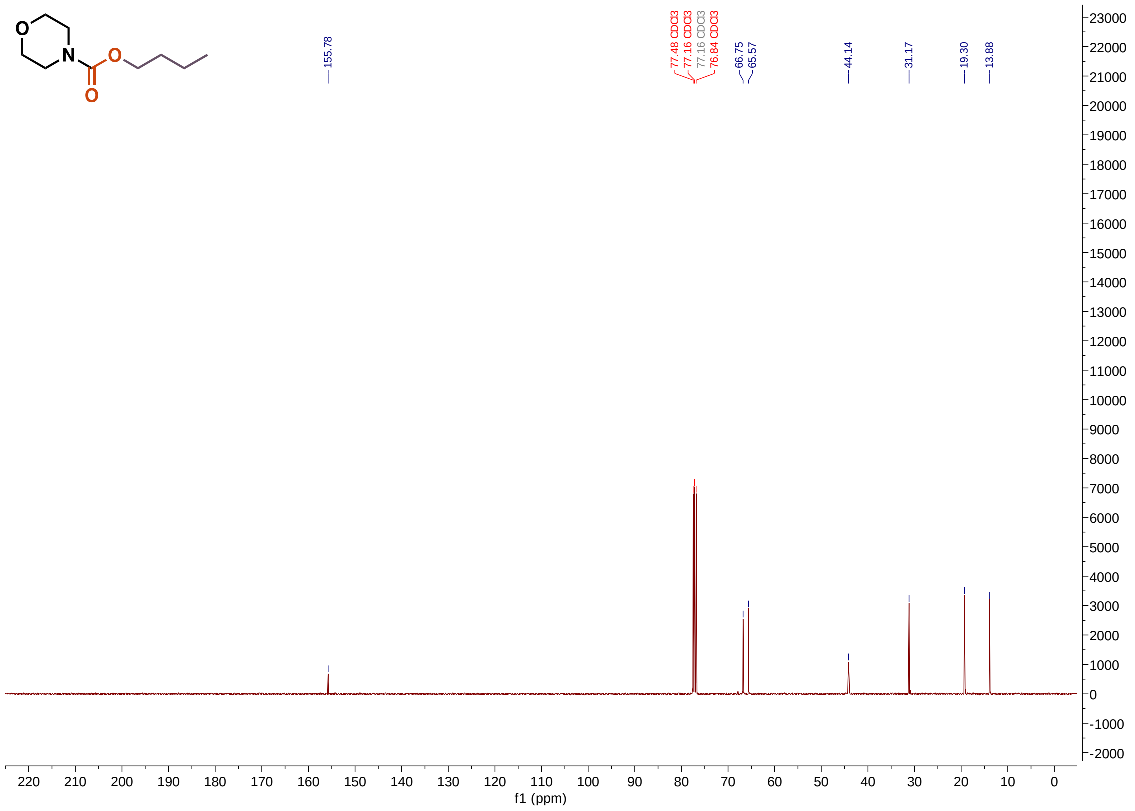

Figure S41. ^{13}C NMR of **3r**

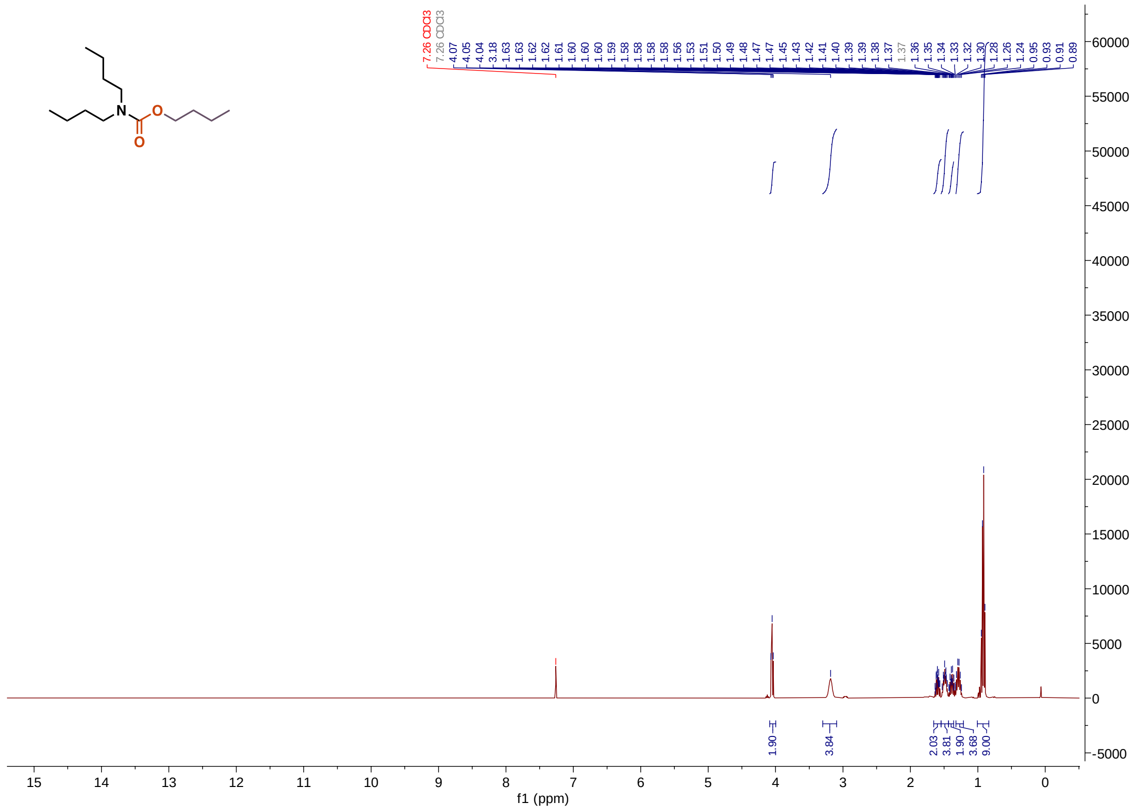

Figure S42. ^{1}H NMR of **3s**

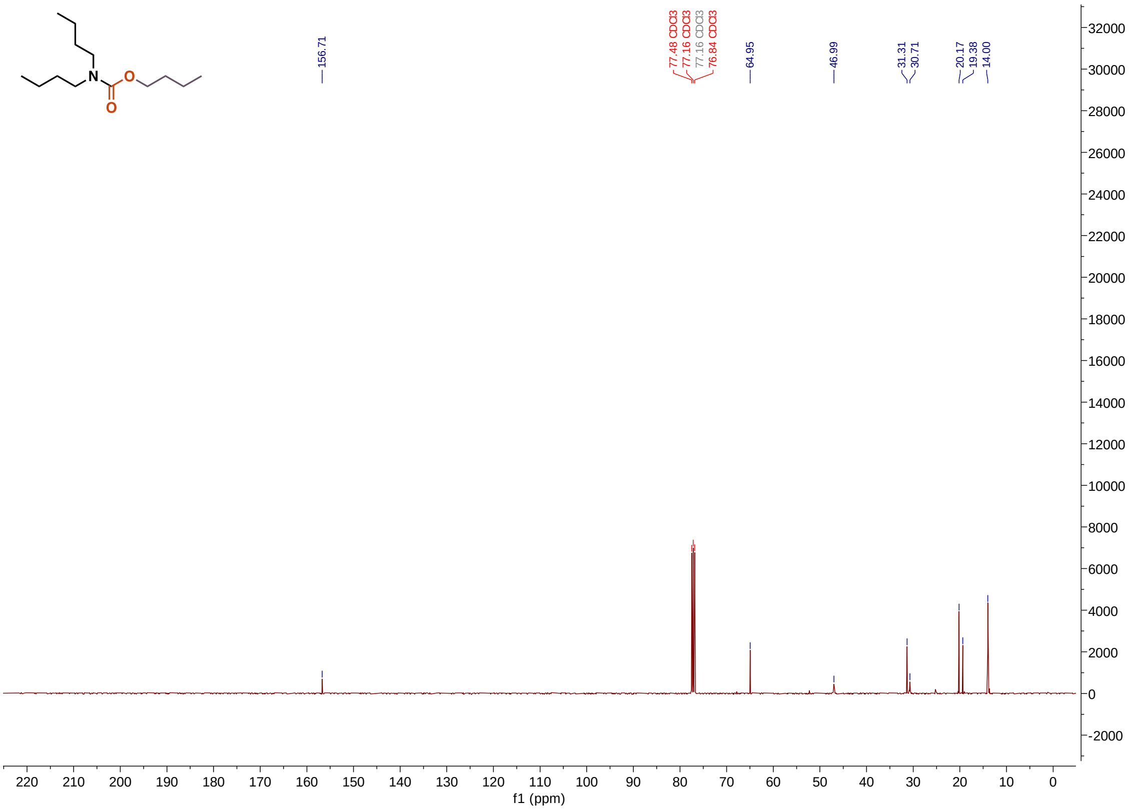

Figure S43. ^{13}C NMR of **3s**

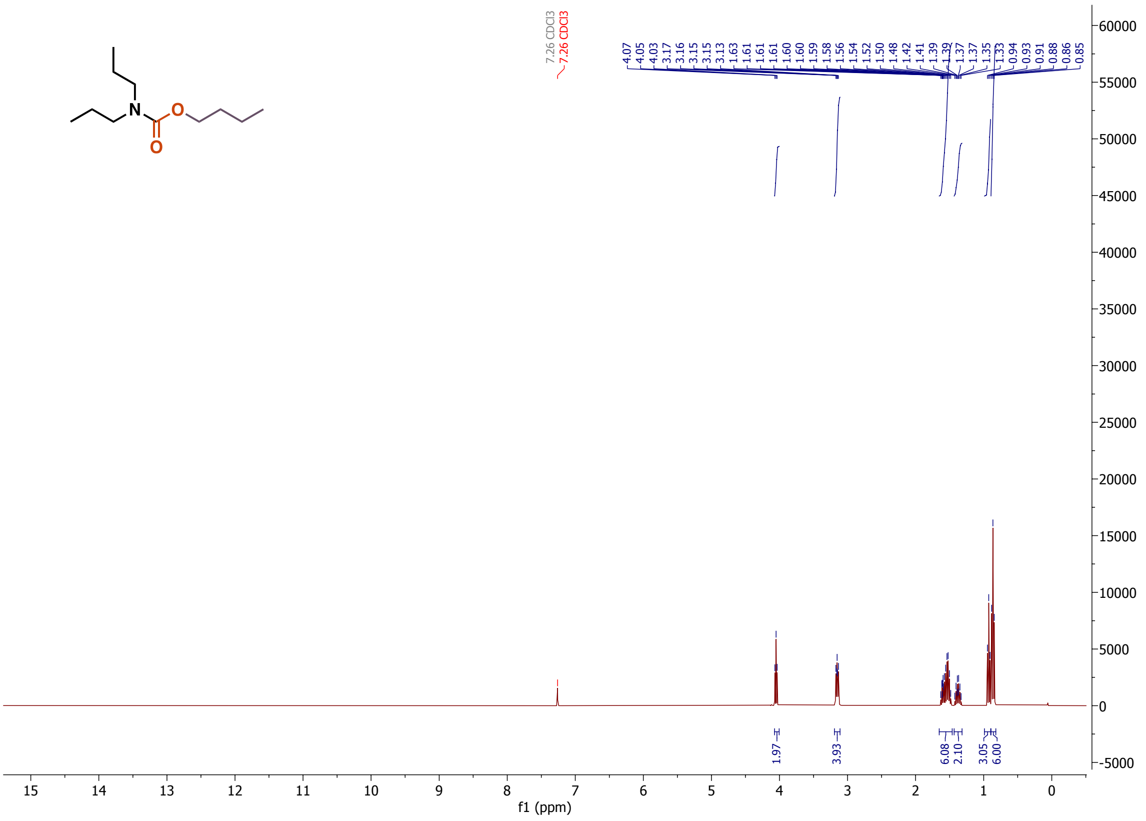

Figure S44. ^{1}H NMR of **3t**

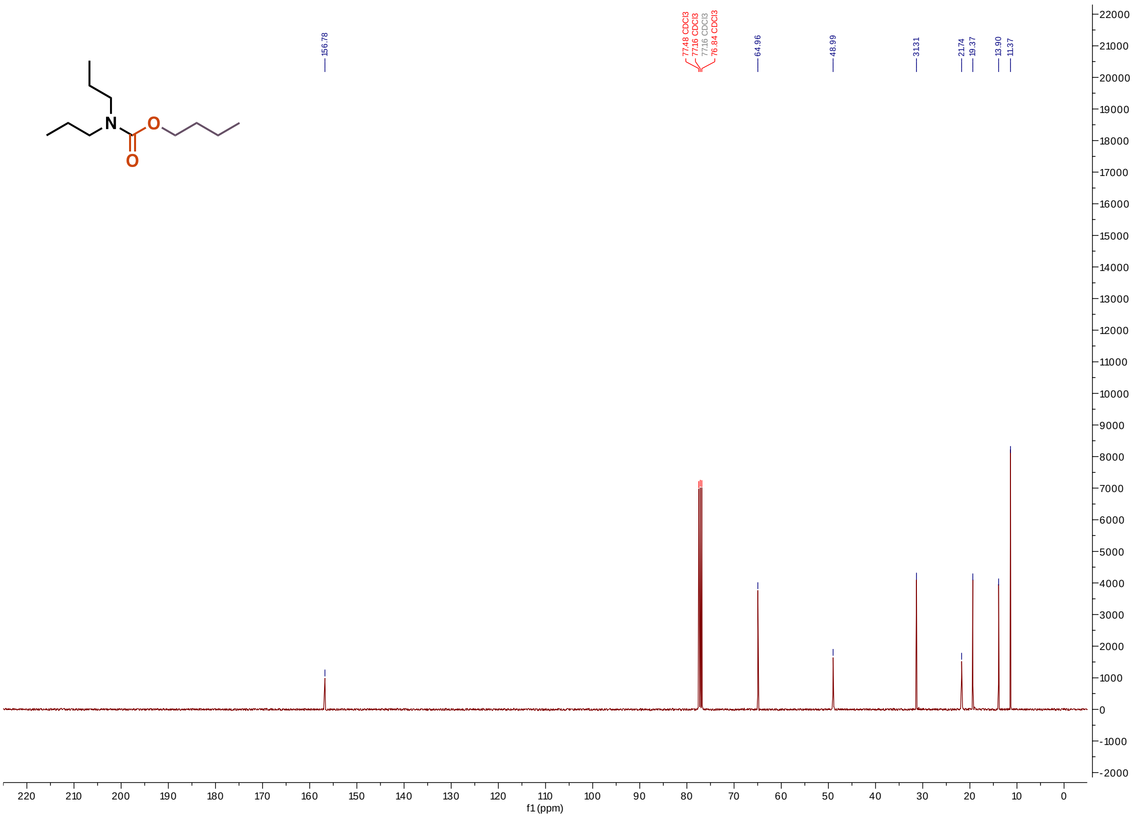

Figure S45. ^{13}C NMR of **3t**

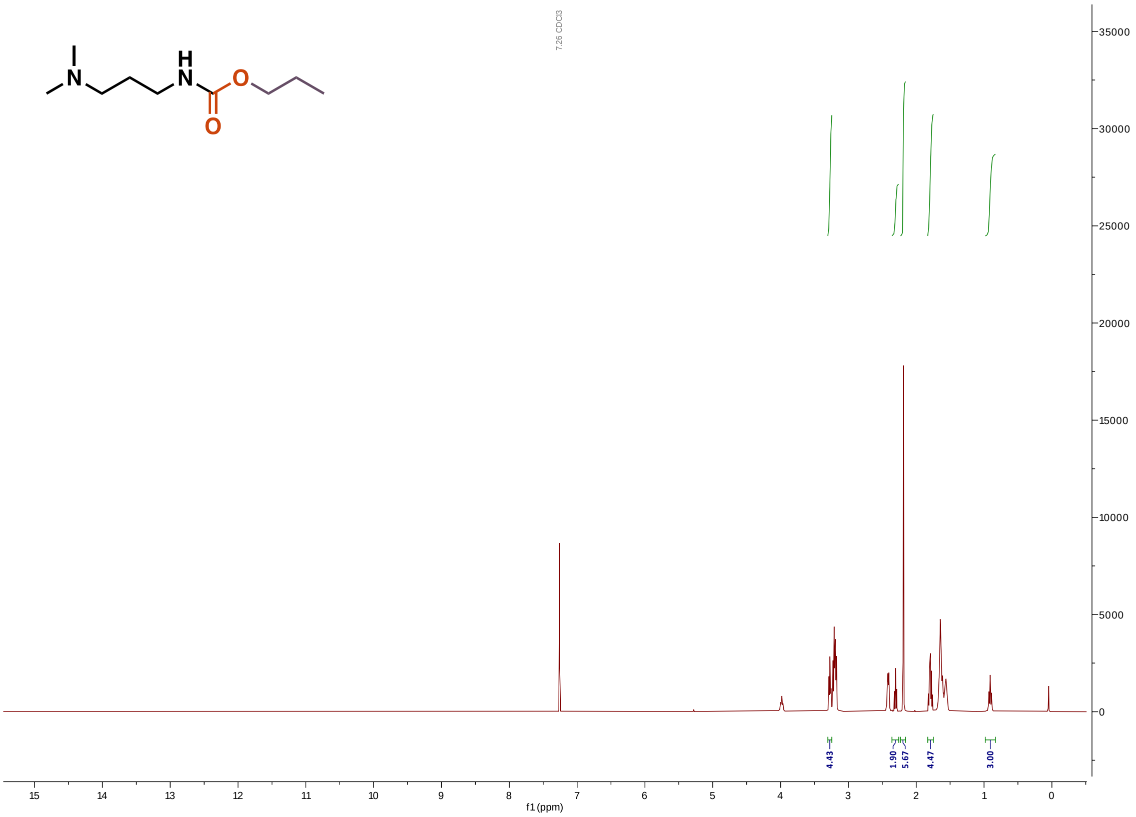

Figure S46. ^{1}H NMR of **3u**

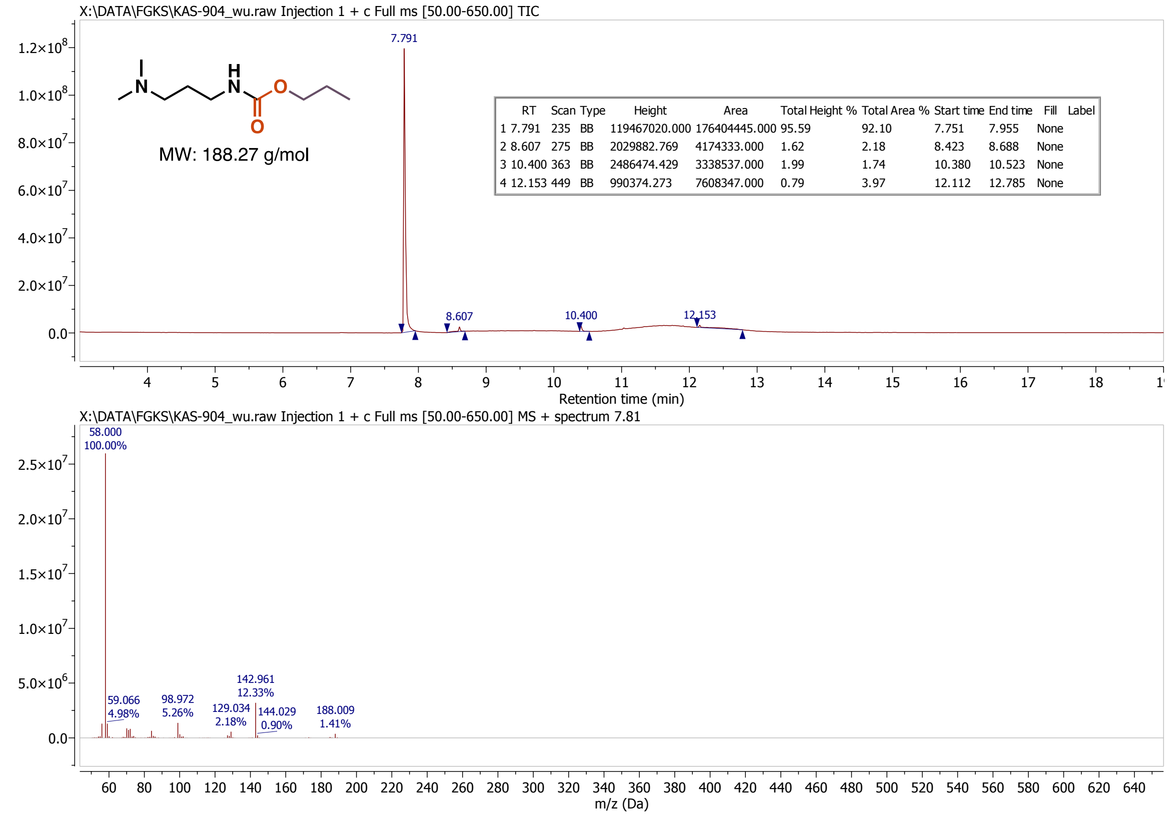

Figure S47. GC-MS chromatogram of **3u**

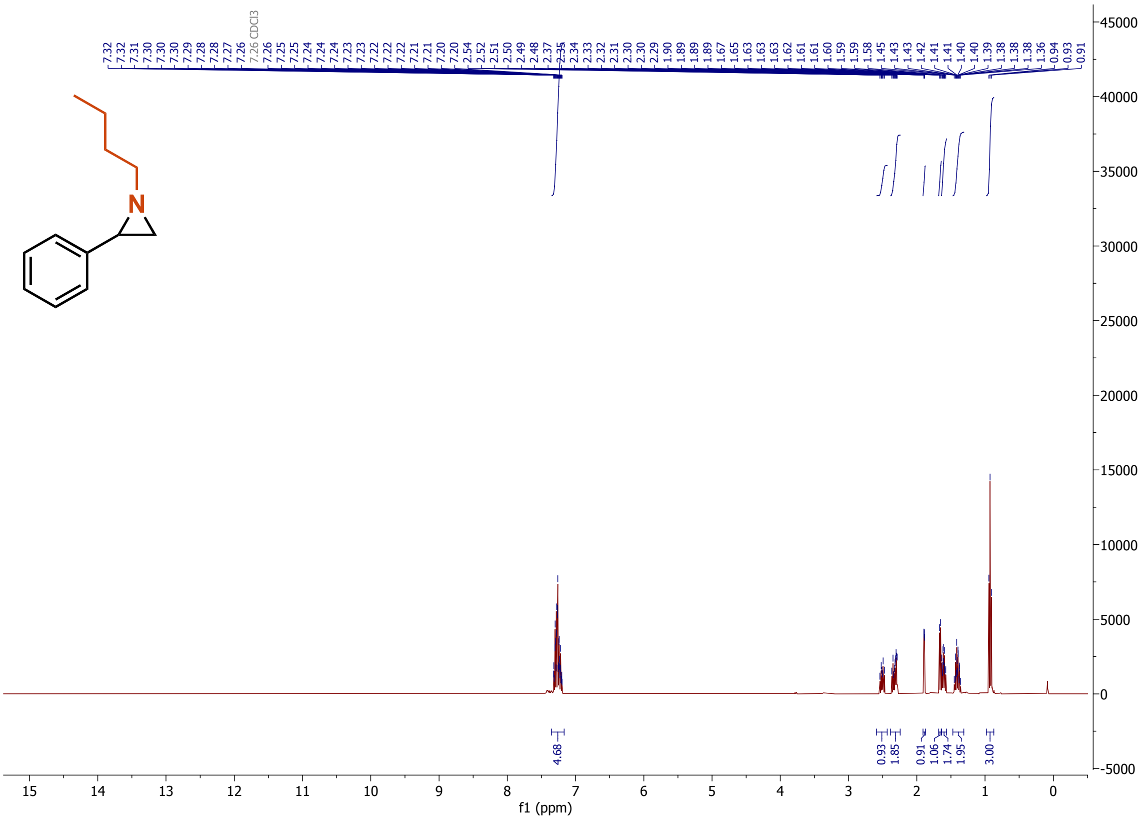

Figure S48. ^{1}H NMR of **4a**

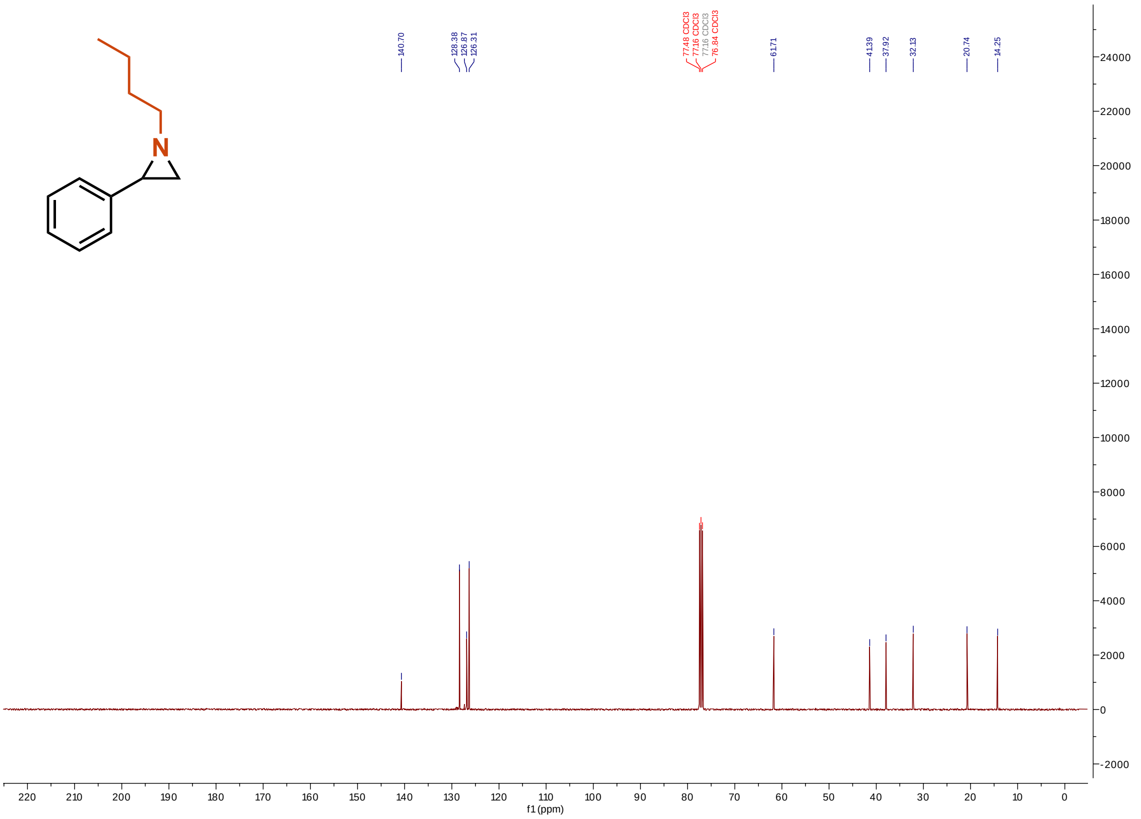

Figure S49. ^{13}C NMR of **4a**

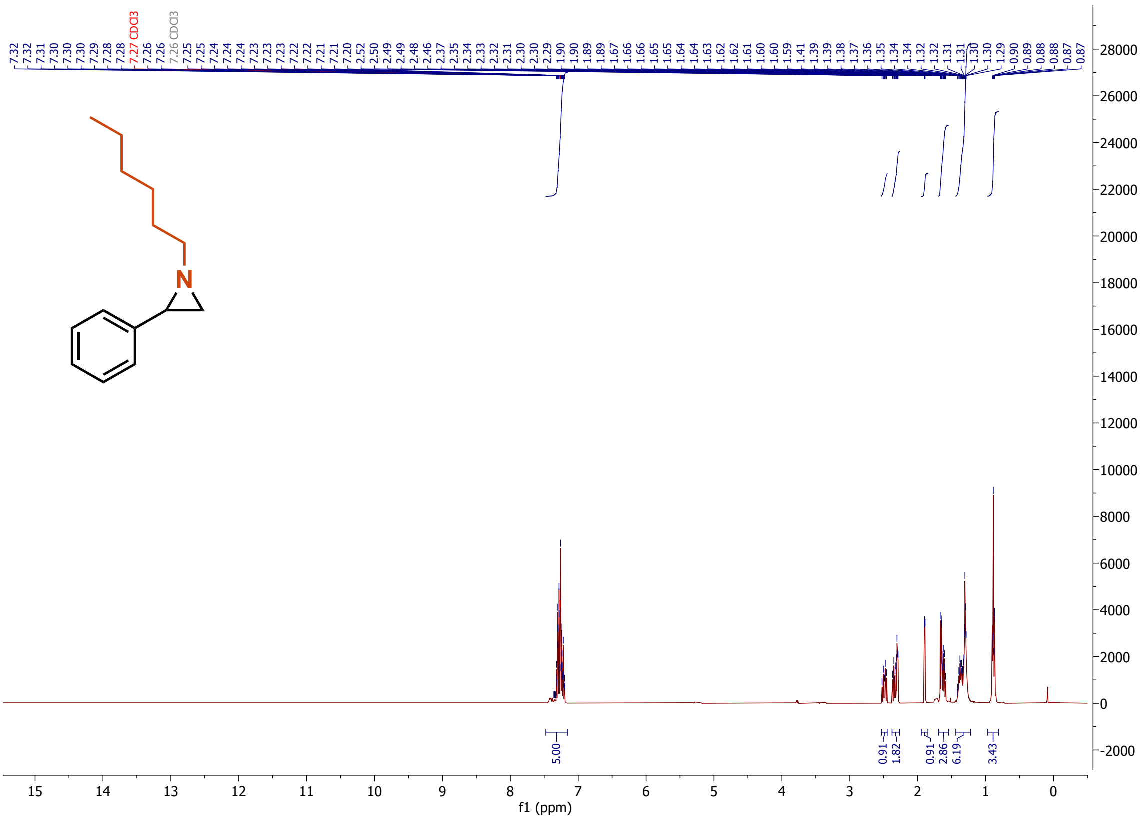

Figure S50. ^{1}H NMR of **4b**

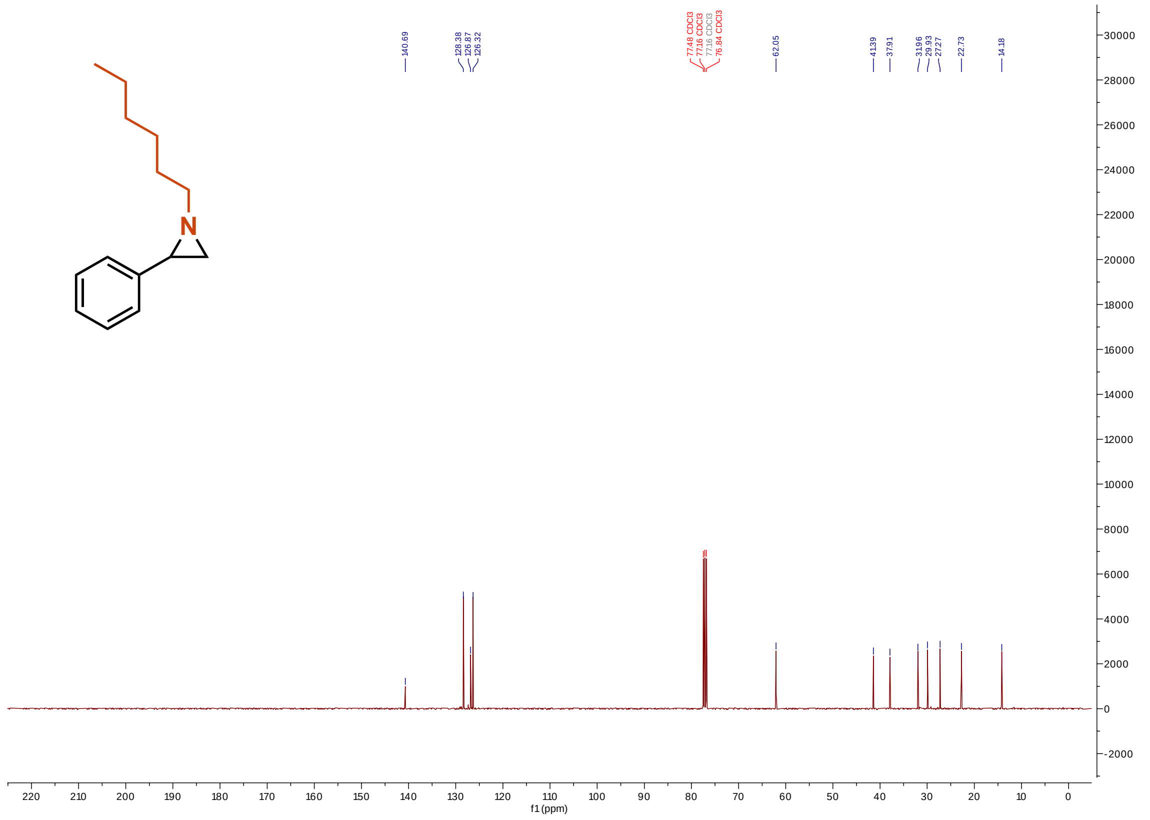

Figure S51. ^{13}C NMR of **4b**

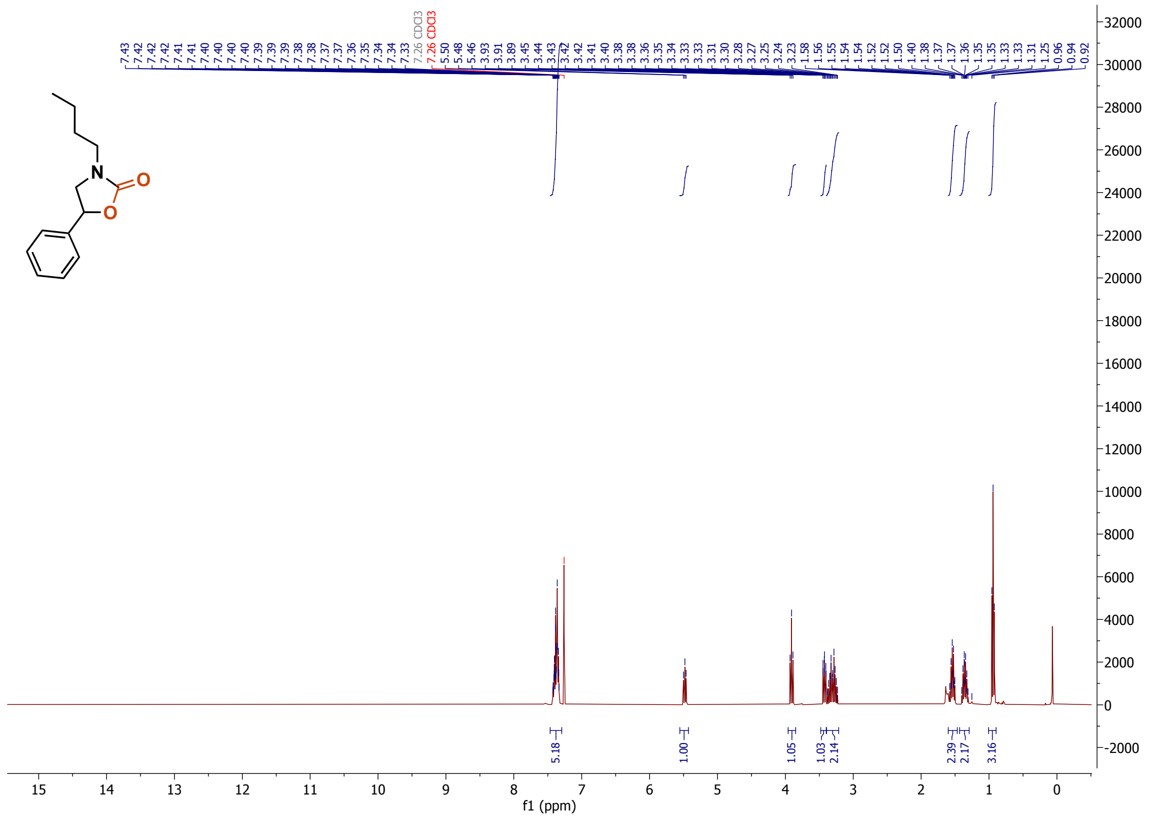

Figure S52. ^{1}H NMR of **5a**

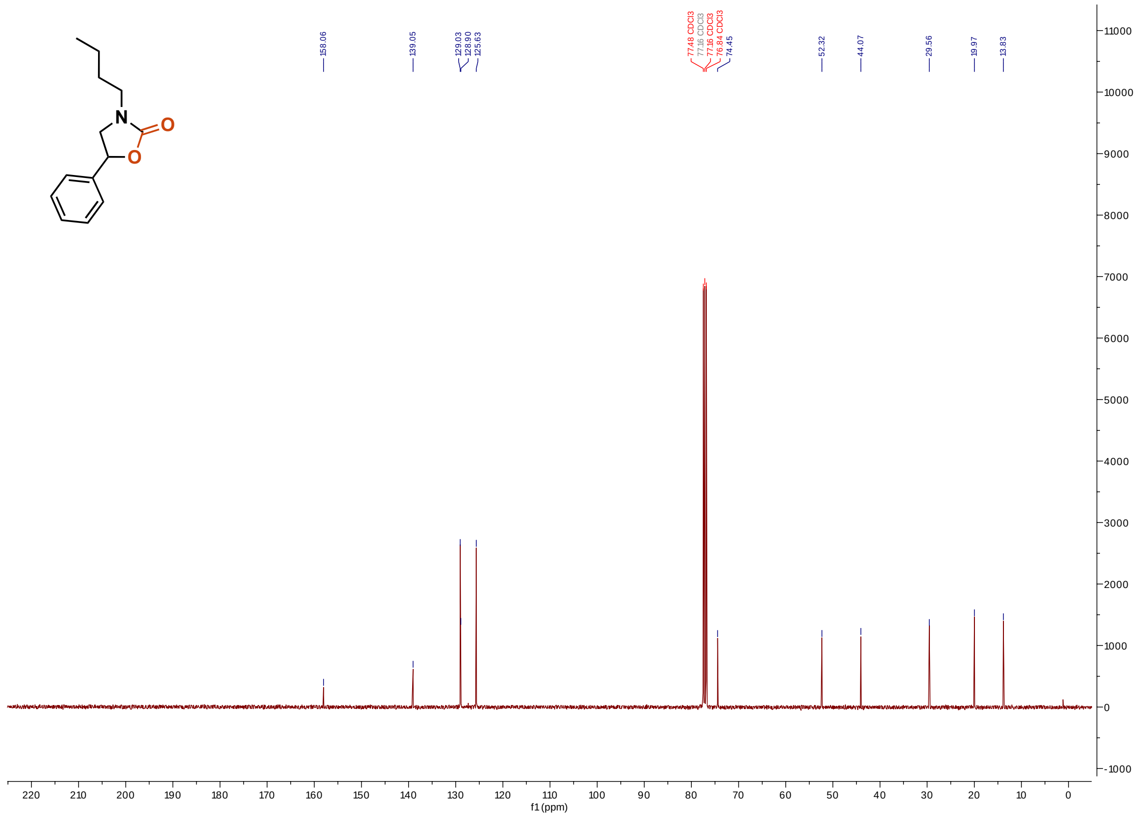

Figure S53. ^{13}C NMR of **5a**

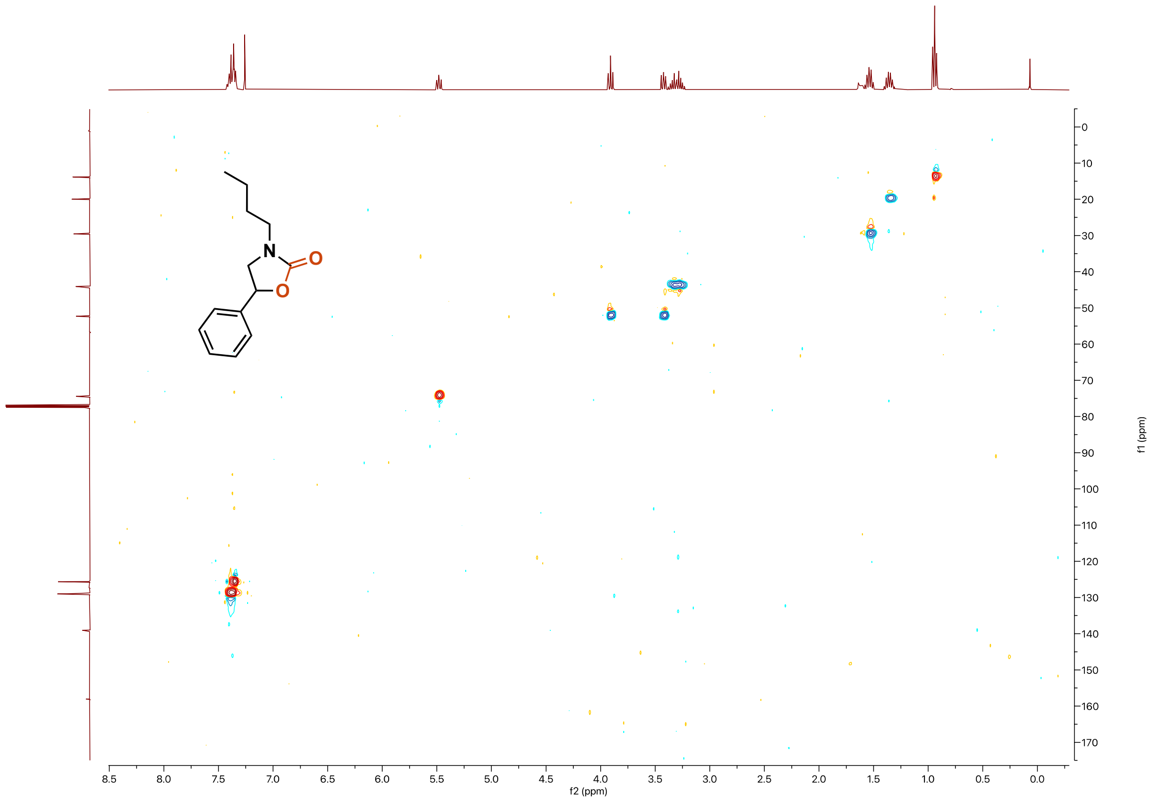

Figure S54. HSQC NMR of **5a**

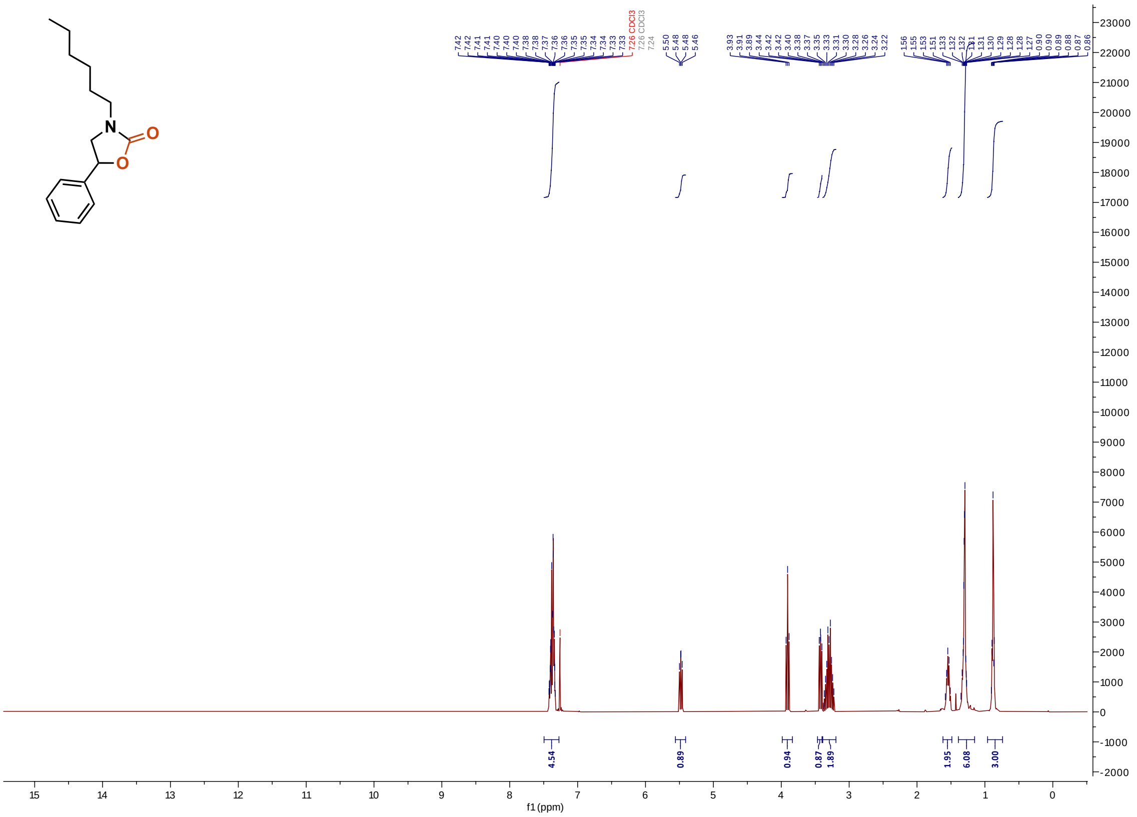

Figure S55. ^{1}H NMR of **5b**

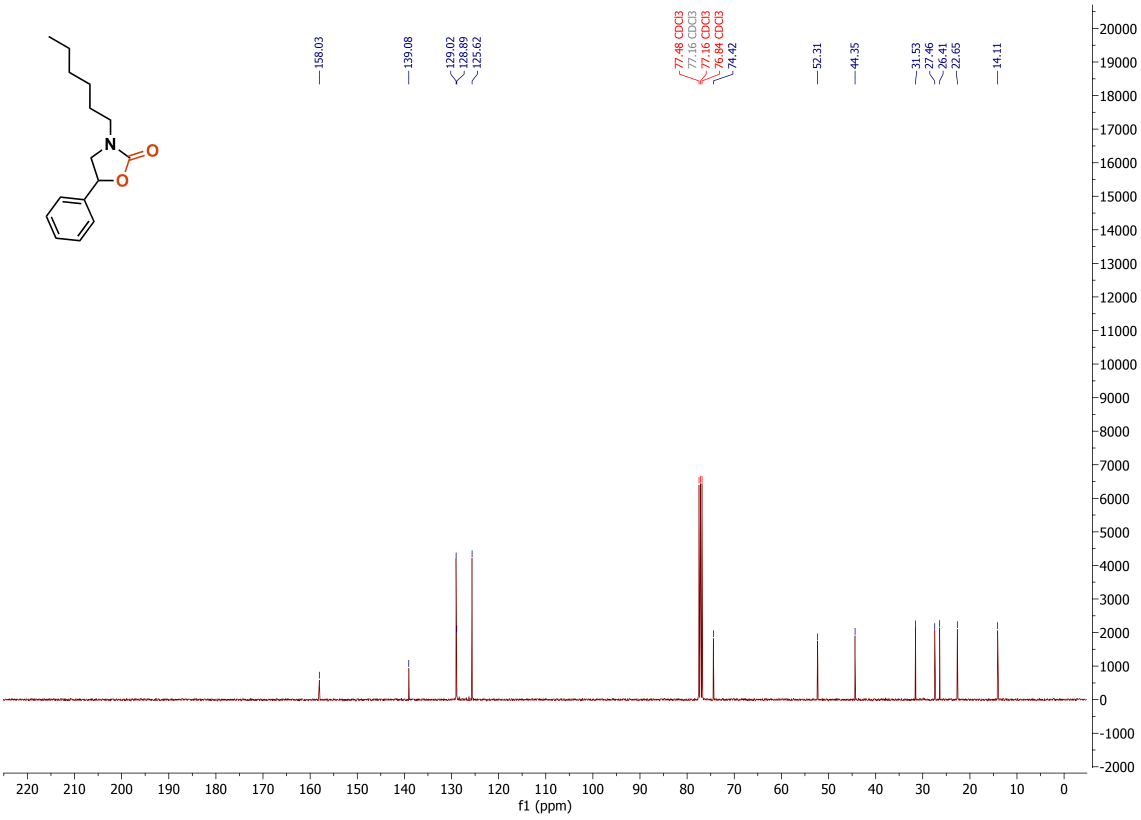

Figure S56. ^{13}C NMR of **5b**

7. Chiral HPLC traces

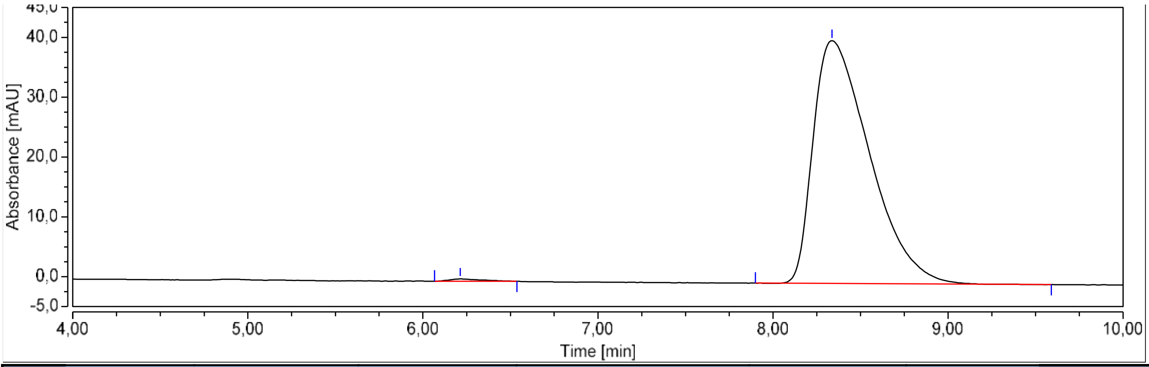

Integration Results							
No.	Peak Name	Retention Time min	Area mAU*min	Height mAU	Relative Area %	Relative Height %	Amount n.a.
1		6,213	0,079	0,390	0,52	0,95	n.a.
2		8,337	15,118	40,599	99,48	99,05	n.a.
**Total:**			**15,197**	**40,989**	**100,00**	**100,00**	

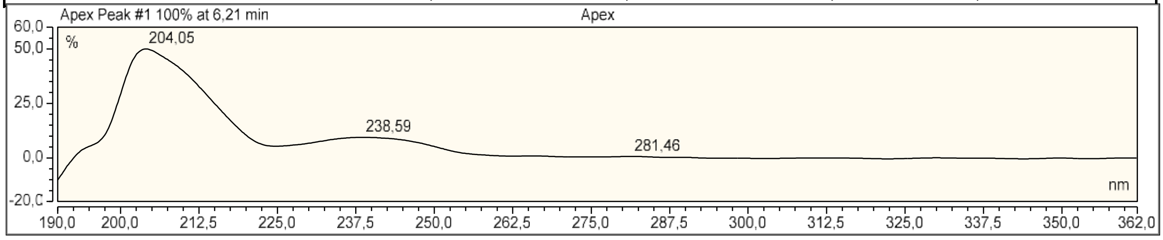

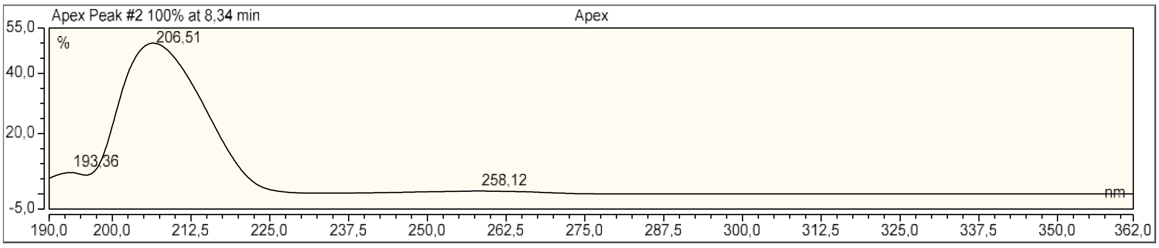

Figure S57. Chiral HPLC chromatogram of **3n**

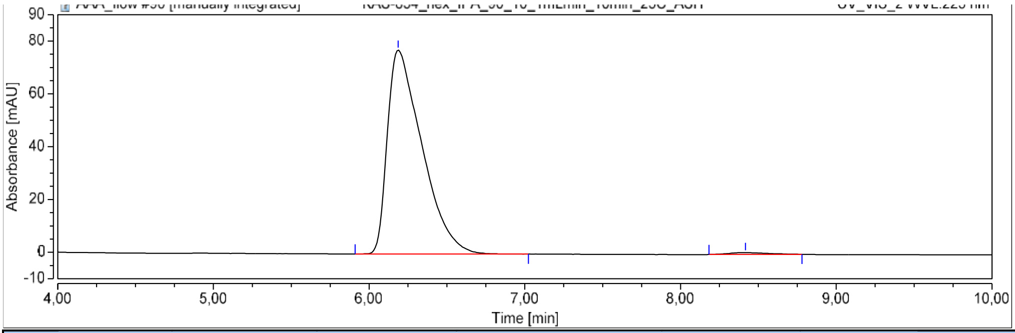

Integration Results							
No.	Peak Name	Retention Time min	Area mAU*min	Height mAU	Relative Area %	Relative Height %	Amount n.a.
1		6,187	19,855	77,232	99,05	99,12	n.a.
2		8,417	0,191	0,686	0,95	0,88	n.a.
**Total:**			**20,046**	**77,918**	**100,00**	**100,00**	

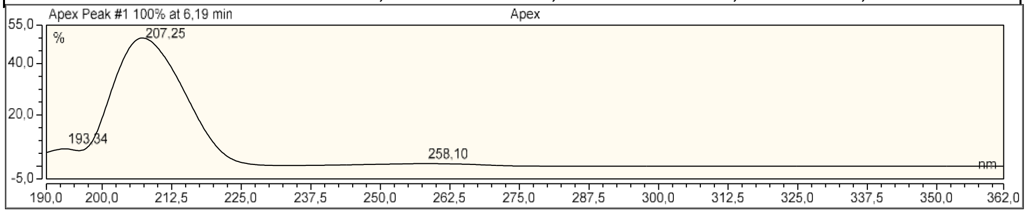

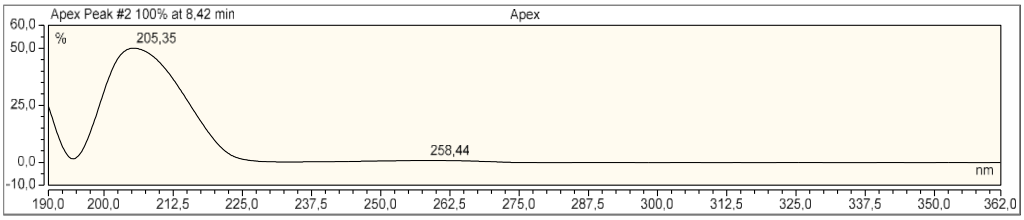

Figure S58. Chiral HPLC chromatogram of **3o**

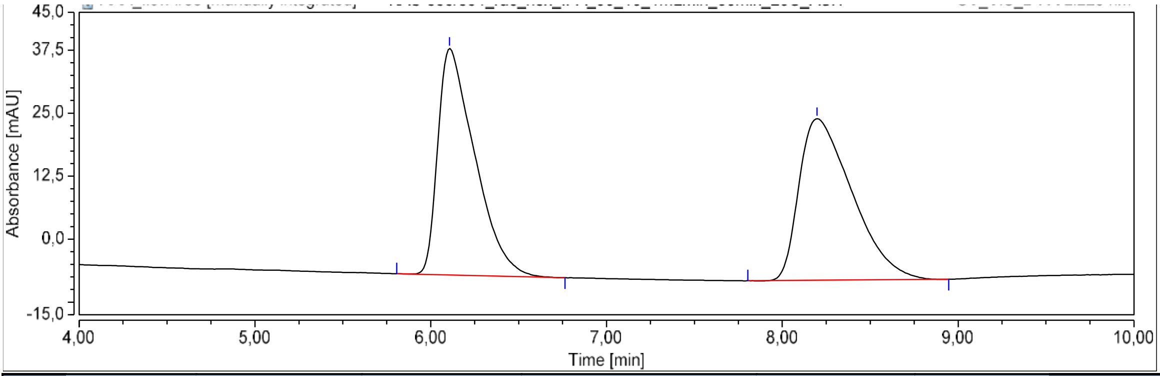

Integration Results							
No.	Peak Name	Retention Time min	Area mAU*min	Height mAU	Relative Area %	Relative Height %	Amount n.a.
1		6,107	10,932	44,953	49,37	58,36	n.a.
2		8,197	11,212	32,071	50,63	41,64	n.a.
**Total:**			**22,144**	**77,024**	**100,00**	**100,00**	

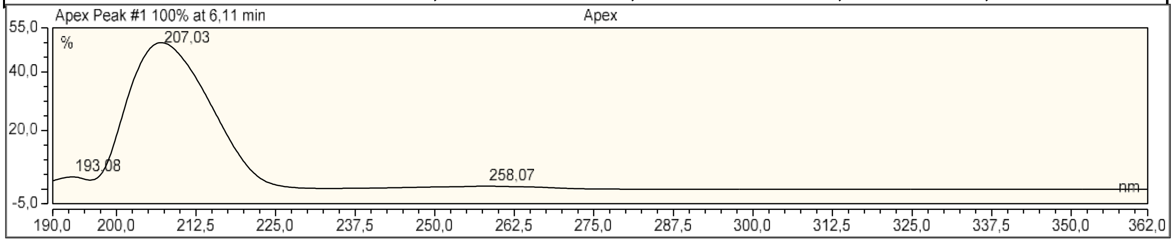

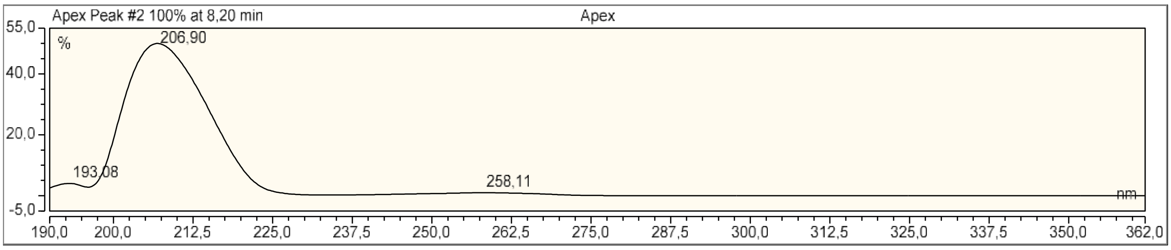

Figure S59. Chiral HPLC chromatogram of **3n-3o** racemic

8. References

(1) Takeuchi, K.; Chen, M.-Y.; Yuan, H.-Y.; Koizumi, H.; Matsumoto, K.; Fukaya, N.; Choe, Y.-K.; Shigeyasu, S.; Matsumoto, S.; Hamura, S.; Choi, J.-C., *N*-Aryl and *N*-Alkyl Carbamates from 1 Atmosphere of CO_2. *Chem. Eur. J.* **2021,** *27* (72), 18066-18073.

(2) Yang, X.; Zhang, Y.; Ma, D., Synthesis of Aryl Carbamates Via Copper-Catalyzed Coupling of Aryl Halides with Potassium Cyanate. *Adv. Synth. Catal.* **2012,** *354* (13), 2443-2446.

(3) Helberg, J.; Ampßler, T.; Zipse, H., Pyridinyl Amide Ion Pairs as Lewis Base Organocatalysts. *J. Org. Chem.* **2020,** *85* (8), 5390-5402.

(4) Kumar, S. V.; Ma, D., Synthesis of *N*-(Hetero)Aryl Carbamates Via Cui/Mnao Catalyzed Cross-Coupling of (Hetero)Aryl Halides with Potassium Cyanate in Alcohols. *J. Org. Chem.* **2018,** *83* (5), 2706-2713.

(5) Zhang, B.; Deng, W.; Xu, Z.-Y., Palladium-Catalyzed Carbonylation of Amines with Mo $(CO)_6$ as the Carbonyl Source. *Organometallics* **2023,** *42* (7), 588-596.

(6) Roy, S.; Majumdar, K. K., A Practical Organotin(IV) Catalyst for Urethan and Polyurethan Technology. *Synth. Commun.* **1994,** *24* (3), 333-340.

(7) Ghiazza, C.; Wagner, L.; Fernández, S.; Leutzsch, M.; Cornella, J., Bio-Inspired Deaminative Hydroxylation of Aminoheterocycles and Electron-Deficient Anilines. *Angew. Chem. Int. Ed.* **2023,** *62* (2), e202212219.

(8) de Castro, M. S.; Domínguez, P.; Sinisterra, J. V., Enzymatic Amidation and Alkoxycarbonylation of Amines Using Native and Immobilised Lipases with Different Origins: A Comparative Study. *Tetrahedron* **2000,** *56* (10), 1387-1391.

(9) Ogbu, I. M.; Lusseau, J.; Kurtay, G.; Robert, F.; Landais, Y., Urethanes Synthesis from Oxamic Acids under Electrochemical Conditions. *Chem. Commun.* **2020,** *56* (81), 12226-12229.

(10) Gómez-Parra, V.; Sánchez, F.; Torres, T., Carbamates from Secondary Amines and Alkyl Chlorides under Phase-Transfer Conditions. *Synthesis (Stuttgart)* **1985,** (3), 282-285.

(11) Kong, D.-L.; He, L.-N.; Wang, J.-Q., Polyethylene Glycol–Enhanced Chemoselective Synthesis of Organic Carbamates from Amines, CO_2, and Alkyl Halides. *Synth. Commun.* **2011,** *41* (22), 3298-3307.

(12) Matlock, J. V.; Fritz, S. P.; Harrison, S. A.; Coe, D. M.; McGarrigle, E. M.; Aggarwal, V. K., Synthesis of A-Substituted Vinylsulfonium Salts and Their Application as Annulation Reagents in the Formation of Epoxide- and Cyclopropane-Fused Heterocycles. *J. Org. Chem.* **2014,** *79* (21), 10226-10239.

(13) Saptal, V. B.; Bhanage, B. M., *N*-Heterocyclic Olefins as Robust Organocatalyst for the Chemical Conversion of Carbon Dioxide to Value-Added Chemicals. *ChemSusChem* **2016,** *9* (15), 1980-1985.

(14) Yang, Z.-Z.; Li, Y.-N.; Wei, Y.-Y.; He, L.-N., Protic Onium Salts-Catalyzed Synthesis of 5-Aryl-2-Oxazolidinones from Aziridines and CO_2 under Mild Conditions. *Green Chem.* **2011,** *13* (9), 2351-2353.

(15) Shi, Y.; Tang, B.; Jiang, X.-L.; Jiao, Y.-E.; Xu, H.; Zhao, B., Highly Effective CS_2 Conversion with Aziridines Catalyzed by Novel [Dy24] Nano-Cages in MOFs under Mild Conditions. *Journal of Materials Chemistry A* **2022,** *10* (9), 4889-4894.

(16) Watile, R. A.; Bagal, D. B.; Patil, Y. P.; Bhanage, B. M., Regioselective Synthesis of 5-Aryl-2-Oxazolidinones from Carbon Dioxide and Aziridines Using Br−Ph3+Ppeg600p+Ph3br− as an Efficient, Homogenous Recyclable Catalyst at Ambient Conditions. *Tetrahedron Lett.* **2011,** *52* (48), 6383-6387.
